# Ex vivo bone marrow subniches influence the fate of human antibody-secreting cells

**DOI:** 10.1126/sciadv.adz3976

**Published:** 2026-09-11

**Authors:** Liana Kramer, Zhonghao Dai, Jenna Corbin, Rachel Ringquist, Eshant Bhatia, Ingrid Petersen, Delta Ghoshal, Ritika Jain, Valeria M. Juarez, Zhe Zhong, Savi Agarwal, F. Eun-Hyung Lee, Steven Goudy, Ankur Singh, Krishnendu Roy

**Affiliations:** ^1^ Coulter Department of Biomedical Engineering, Georgia Institute of Technology and Emory University, Atlanta, GA, USA.; ^2^ School of Chemical and Biomolecular Engineering, Georgia Institute of Technology, Atlanta, GA, USA.; ^3^ Woodruff School of Mechanical Engineering, Georgia Institute of Technology, Atlanta, GA, USA.; ^4^ Department of Medicine, Division of Pulmonary, Allergy, Critical Care, and Sleep Medicine, Emory University, Atlanta, GA, USA.; ^5^ Department of Medicine, Lowance Center for Human Immunology, Emory University, Atlanta, GA, USA.; ^6^ Department of Otolaryngology, Emory University, Atlanta, GA, USA.; ^7^ Department of Pediatric Otolaryngology, Children’s Healthcare of Atlanta, Atlanta, GA, USA.; ^8^ Parker H. Petit Institute for Bioengineering and Bioscience, Georgia Institute of Technology, Atlanta, GA, USA.; ^9^ Marcus Center for Therapeutic Cell Characterization and Manufacturing (MC3M), Georgia Institute of Technology, Atlanta, GA, USA.; ^10^ Department of Biomedical Engineering, Vanderbilt University, Nashville, TN, USA.; ^11^ Department of Pathology, Microbiology, and Immunology, Vanderbilt University Medical Center, Nashville, TN, USA.; ^12^ Department of Chemical and Biomolecular Engineering, Vanderbilt University, Nashville, TN, USA.

## Abstract

Long-term humoral immunity relies on long-lived plasma cells in the bone marrow (BM). However, the processes governing plasma cell transport, positioning, and longevity within the BM niche remain poorly understood, especially in humans. Most existing knowledge comes from mouse studies or limited human-based models, which makes translating findings to human biology challenging. Here, we introduce a physiologically relevant human bone marrow-on-a-chip (hBMOC) model to investigate the behavior and interactions of human immune organoid-derived antibody-secreting cells (ASCs) within the human BM microenvironment. The hBMOC model is microvascular and perfusable and incorporates both endosteal and perivascular niches. We demonstrate that human ASCs migrate through blood vessels, accumulating and clustering in perivascular areas where they are closely associated with critical survival factors. In addition, we found that the presence of the endosteal niche substantially affects human ASC survival, movement, and retention, underscoring the dynamic interactions among human BM subniches that regulate ASC activity. We observed that a subset of human ASCs exhibits a dynamic stop-and-go migration pattern partially regulated by CXCR4-CXCL12 signaling. These findings provide direct insight into human ASC biology that has remained poorly defined, including their niche-specific localization, survival, and migratory dynamics within a three-dimensional human bone marrow microenvironment. Our results emphasize the distinct and cooperative roles of perivascular and endosteal compartments in supporting human ASC fate, offering previously inaccessible mechanistic insights into how human BM niches regulate plasma cells. This work lays the groundwork for studying plasma cell aging, vaccine durability, and disease-related dysfunction in human ASC maintenance and persistence.

## INTRODUCTION

The humoral immune response is crucial for maintaining immunity and plays a fundamental role in the effectiveness of antiviral responses and vaccinations. This long-term protection is facilitated by antibody-secreting cells (ASCs), particularly plasma cells (PC), which are found in the bone marrow (BM). However, despite the essential role of sustaining elevated antibody levels, the mechanisms that regulate human ASC and PC localization in the human BM, as well as their functions, remain poorly understood (*1*).

PCs develop from B cells located in secondary lymphoid organs, such as lymph nodes, the spleen, and tonsils. Here, a naïve B cell interacts with an antigen and undergoes maturation through the germinal center (GC) process, resulting in the formation of end-stage ASCs and memory B cells (*2*, *3*). ASCs, traditionally categorized as plasmablasts and PCs, differentiate from activated B cells in lymph nodes and mucosal sites into short-lived PCs (*4*). However, through an unclear process, a subset of these cells migrates to various locations, such as the BM, where they can persist as long-lived producers of antibodies (*5*). This unique property is crucial for long-term immune protection after vaccination or infection (*6*). Despite our understanding of the BM microenvironment (*7*–*11*), the role of individual components in the human BM niche in the programming of human ASCs remains elusive.

**Fig. 1.**
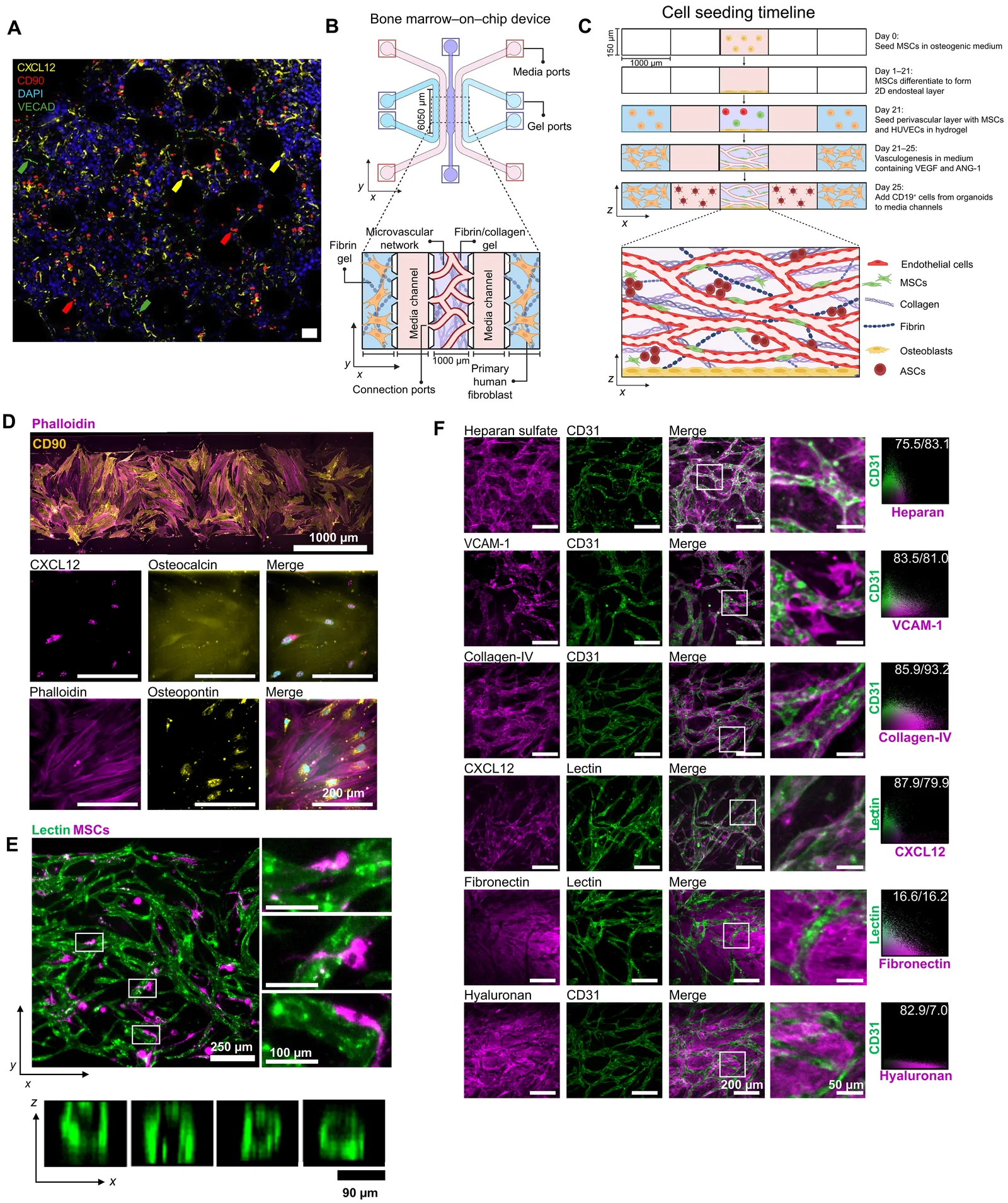
BM-on-a-chip recapitulates the human BM microenvironment. (**A**) The spatial architecture of human BM features CD90^+^ mesenchymal stem cells (MSCs), VECAD + sinusoids, and CXCL12 + stroma. DAPI, 4′,6-diamidino-2-phenylindole. (**B**) An illustrative representation of the human BM-on-a-chip (hBMOC) platform is shown. The schematic depicts a unit from a 4 × 2 grid of five-channel devices placed on a bottomless 96-well plate. (**C**) The timeline for cell seeding in the hBMOC. On day 0, MSCs are introduced into the central channel. From days 1 to 21, these MSCs undergo differentiation in osteogenic medium. On day 21, human umbilical vein endothelial cells (HUVECs) and MSCs are placed in the central channel within a collagen-fibrin gel, whereas normal human lung fibroblasts (NHLFs) are added to the outer channels. The devices are supplied with vasculogenic medium through parallel channels, facilitating the formation of a vascular network. On day 29, ASCs are introduced into the media channels. Created in BioRender. L.K. (2024); https://BioRender.com/0s97l6a. (**D**) Confocal microscopy images of the 2D endothelial niche after 21 days of differentiation. Scale bars, 1000 μm (top) and 200 μm (bottom). (**E**) Top: MSCs (in magenta) extend along and around the vessels (in green). Bottom: Vertical cross sections of vessels, revealing open lumens. (**F**) The colocalization analysis provides values representing the percentage of voxels exhibiting fluorescence intensity for marker A (*y* axis) above the threshold while also having a corresponding fluorescence intensity for marker B (*x* axis) above this threshold, and vice versa. The vessel counterstain is shared for collagen-IV and hyaluronan.

The human BM niche that maintains ASCs is a complex environment composed of multiple interacting subniches, characterized by stromal cell types, extracellular matrices (ECMs), and biochemical and physical components (*12*). The human BM consists of the endosteal and the perivascular niches rather than a single uniform environment (*12*). The endosteal niche is situated at the interface between trabecular bone and BM and primarily consists of osteoblasts, osteoclasts, and osteocytes (*13*). The perivascular niche is rich in blood vessels and features a high concentration of endothelial cells, mesenchymal stromal cells (MSCs), and smooth muscle cells. Among these, MSCs are regarded as the primary cell type in the BM niche, providing crucial support for ASC survival via both cell-cell interactions and soluble factors (*14*). A recent study used codetection by indexing (CODEX) to spatially profile more than 1.2 million cells in human BM and integrated single-cell RNA sequencing and CODEX data to connect predicted cellular signaling with spatial proximity (*12*). These studies illustrate the structure and composition of human BM, highlighting the existence of CD90^+^ MSCs, vascular endothelial cadherin (VECAD) + sinusoids, and C-X-C motif chemokine 12 (CXCL12) + stroma [Fig. 1A, reanalyzed for selective markers from a public repository (*12*)]. However, other cell types, such as eosinophils, basophils, monocytes, osteoclasts, and osteoblasts, have also been implicated in potentially regulating ASC survival (*15*–*18*).

Our current knowledge of the role of the BM niche in regulating ASC fate primarily comes from mouse models or in vitro two-dimensional (2D) cultures of human BM cells. However, these models do not accurately reflect the complex microenvironment of human BM. Traditional methods for investigating ASCs within BM niches typically use genetically modified cells or mice and involve disrupting niche factors by assessing the expression of specific reporter genes (*19*). Time-resolved imaging techniques, including intravital imaging that involves removing tissue around the tibia and thinning the bone to expose the marrow, have demonstrated that BM-resident PCs in mice are migratory. Their migration is regulated by CXCR4 and integrin α_4_β_1_ (VLA-4), and these cells often cluster together. Mesenchymal BM cells secrete stromal cell–derived factor 1, commonly referred to as CXCL12, which interacts with CXCR4. Nonetheless, the lack of intravital imaging in human BM restricts our comprehension of the spatiotemporal characteristics of live human ASCs within their native BM niches. As a result, much of the research focused on the factors that promote the survival of human ASCs has relied on in vitro studies in which these cells are cocultured with BM mononuclear adherent cells or MSCs and subjected to various cytokine combinations (*14*, *20*–*22*). A recent study established a cell-free 2D culture that incorporates secreted factors from primary BM MSCs and a hypoxic environment to sustain circulating human early ASCs in vitro for over 50 days (*14*). This study offers important insights into the role of MSC-derived fibronectin in ASC survival. However, it does not encompass the complexities of the BM survival niche, limiting its ability to inform on ASC interactions with subniches, chemokines, vascular networks, and the complex three-dimensional (3D) ECM networks (*12*, *19*). Consequently, replicating the architectural, cellular, and molecular intricacies of the 3D human BM environment in vitro offers a unique opportunity to investigate the signals that facilitate interactions between ASCs, various cell types, and ECM components within the BM. Various tissue-on-chip models have been established to mimic the BM microenvironment for studying cancer and hematopoiesis (*23*–*27*), including our recent research on hematopoietic stem cells in the BM niche (*28*, *29*). These human BM–on–chip (hBMOC) studies have not shown the fate of the ASC compartment and its maintenance within the BM, particularly regarding subniches.

How ASCs integrate into, interact with, and are sustained by the highly specialized, multiniche architecture of the human BM microenvironment remains poorly understood. Progress in modeling human humoral immunity has been constrained by two major bottlenecks: the reliable generation of human ASCs and the lack of BM systems that allow discrete niches to be systematically deconstructed to define ASC fate. Here, we present an integrated ex vivo platform that combines two engineered human lymphoid tissues: a complex, multiniche hBMOC model representing a primary lymphoid organ and a human secondary lymphoid organoid–derived ASCs. We recently developed biomaterials-based human lymphoid organoids derived from tonsils or peripheral blood mononuclear cells (PBMCs) that recapitulate germinal center (GC) responses and support the progressive differentiation of ASCs (*30*), which were extensively benchmarked for GC and ASC signatures, including antibody generation, somatic hypermutation, clonal capacities, and B cell receptor–driven class-switching recombination. These attributes make the lymphoid organoid-derived ASCs a viable tool for the generation of a higher number of ASCs for studies in BM niches. By reconstructing and modularly integrating the endosteal and perivascular BM niches, we interrogated ASC maturation, trafficking, and niche-specific interactions within a human BM microenvironment in hBMOC. We find that organoid-derived ASCs access the BM compartment via the vascular interface, preferentially localize to perivascular regions, and form clusters adjacent to blood vessels enriched in key survival cues. In addition, we demonstrate that a bone-like endosteal niche affects ASC movement and differentiation in distinct ways, highlighting the crucial role of niche interactions in governing ASC functionalities within the BM and providing valuable insights into their potential therapeutic applications.

## RESULTS

### hBMOC recapitulates subniche microarchitecture of human BM

We sought to develop a perfusable microvascular hBMOC model that integrates both endosteal and perivascular niches, incorporating human ASCs to investigate their behavior, migration, and interactions within the BM microenvironment. Our approach uses an innovative design in which the human BM–mimicking endosteal and perivascular niches are physically unified on top of each other (*28*, *29*), in contrast to other designs where both niches remain physically separate yet connected through channels (*24*). An individual microfluidic device for hBMOC features five channels. The first is a central chamber known as the “gel channel,” which contains a mixture of collagen, thrombin, and fibrin serving as a BM niche. This central channel is flanked by two media channels on either side and two outer gel channels designated for cultivating fibroblasts that support vascularization. The media channel infused nutrients and growth factors into the gel channel. To enhance throughput and reproducibility, we bonded four sets of two five-channel devices to a bottomless 96-well plate using polydimethylsiloxane (PDMS) channels (Fig. 1, B and C). This configuration uses the wells of the 96-well plate as both media reservoirs and imaging windows for the central channel, facilitating efficient imaging of device replicates. The devices maintain a steady orientation on each plate, ensuring compatibility with automated imaging systems and strong reproducibility.

To create an endosteal subniche, we seeded MSCs at a density of 6 × 10^5^ cells/ml into the central channel and induced to differentiate in osteogenic medium, allowing them to develop into a mineralized endosteum–like niche. The CD90^+^ MSCs established a 2D monolayer that covered the basal surface of the central channel while expressing key markers associated with the endosteal niche, including osteopontin, osteocalcin, and CXCL12 (Fig. 1D). Osteopontin and osteocalcin are ECM proteins found in the bone matrix, located at the interface between the bone surface and marrow in human BM (*12*) and murine femurs (*31*). Although both osteocalcin and osteopontin are secreted proteins, their localization patterns differ because differences in postsecretion binding behavior and matrix association. Osteocalcin is primarily incorporated into the mineralized ECM and exhibits strong affinity for hydroxyapatite, leading to more localized, matrix-associated staining patterns. In contrast, osteopontin is a multifunctional matricellular protein that can associate with both the ECM and cell surfaces via integrin and CD44 interactions and can also remain partially soluble, resulting in broader and sometimes more diffuse distribution patterns. The staining patterns observed for these endosteal niche markers are consistent with published images of in vitro human MSC–derived endosteal cells (*32*–*34*). We have previously (*28*) shown that by day 21, a mineral layer is present, as confirmed by Alizarin red and von Kossa staining. After 21 days of osteogenic differentiation, a fresh mixture of MSCs from the same source and endothelial cells was cocultured in a fibrin-collagen hydrogel that covered the endosteal niche. Concurrently, normal human lung fibroblasts (NHLFs) populated the outer channels, releasing provasculogenic factors into the media channels. Fibroblasts play a crucial role in the formation of vascular networks in this device setup (*28*, *35*). The vascular endothelial growth factor and angiopoietin-1 (ANG-1) were supplemented into the media channels. Angiogenic medium perfuses from these channels into the central gel channel through open ports. As a result, a network of perfusable endothelial cells was established within the central chamber, showcasing open lumens (Fig. 1E), similar to what is observed in tubular vessel formation. The MSCs are closely associated with endothelial cells and vessel-like structures, with their cellular extensions surrounding the vessels (Fig. 1E), indicating a pericyte-like relationship function. These features represent recently reported human BM spatial multiplex imaging studies (*12*), which indicate that CD90^+^ MSCs exhibit close associations with VECAD^+^ vessel-like structures (Fig. 1A). To determine whether the hBMOC captured critical elements of in vivo human BM composition (*12*, *36*–*39*), we characterized the expression of key markers of the BM perivascular niche, including collagen-IV, heparan sulfate, vascular cell adhesion molecule–1 (VCAM-1), and CXCL12. We found that these markers are located near the hBMOC vasculature (Fig. 1F), which further supports the findings from human BM spatial multiplex imaging studies (*12*), where CXCL12 expression was detected close to vessel-like structures (Fig. 1A) (*4*, *40*, *41*).

Next, we quantified the colocalization of BM niche markers in hBMOC. Previous studies on murine BM architecture have shown that 91.2% of VCAM-1^+^ voxels, an endothelial marker, are also CD31^+^ (*31*). We conducted a comparable analysis to measure the colocalization of BM perivascular markers in hBMOC and found that 81% of VCAM-1^+^ voxels were also CD31^+^. Following this, we used colocalization analysis to introduce additional markers and assess whether the hBMOC reflects the selective features of BM. In vivo, type IV collagen and heparan sulfate form sheetlike structures within the endothelial basement membrane of the BM (*31*, *42*), and CXCL12 is expressed by BM arterial endothelial cells and stromal and perivascular cells that ensheathe BM sinusoid vessels (*12*, *31*, *37*, *43*–*48*). Colocalization analysis showed that 93.2% of collagen-IV^+^ voxels and 75.5% of heparan sulfate voxels were CD31^+^ (Fig. 1F). CXCL12 expression was also observed to coincide with the vascular network (Fig. 1F). Osteoblast-lineage/endosteal cells are well-established producers of CXCL12 in the BM niche, and the chemokine is actively secreted (*12*, *37*, *46*–*48*). Secreted CXCL12 does not remain freely diffusible; rather, it binds to ECM components, particularly glycosaminoglycans such as heparan sulfate. This interaction enables CXCL12 to become immobilized and concentrated within basement membrane structures. Therefore, the observed enrichment of CXCL12 near the vasculature in Fig. 1F likely reflects ECM-bound, secreted CXCL12 that has been sequestered within the perivascular basement membrane. This interpretation is further supported by the spatial overlap between CXCL12 and heparan sulfate staining. The BM parenchyma contains fibronectin and hyaluronan proteins, as MSCs and osteoblasts express CD44, a canonical receptor for hyaluronan (*49*), and MSCs are critical producers of fibronectin, a defining ECM protein (*50*, *51*). In hBMOCs, fibronectin and hyaluronan are found in the extracellular space surrounding the vessels (Fig. 1F). Notably, only 16.2% of fibronectin^+^ voxels and 7% of hyaluronan^+^ voxels also express CD31^+^. These findings, along with previous characterization of hBMOC devices, emphasize the spatial differentiation of bone matrix–associated proteins (osteocalcin and osteopontin) in the endosteal layer, vascular basement membrane proteins (collagen-IV and heparan sulfate) surrounding the vessels, and BM parenchyma proteins (fibronectin and hyaluronan) distributed throughout the hydrogel. This replication of the unique features of the BM niche’s microarchitecture and proteins highlights the physiological relevance of hBMOCs in studying ASC behavior.

### ASCs traverse the vasculature to localize in the perivascular niche of the hBMOC

**Fig. 2.**
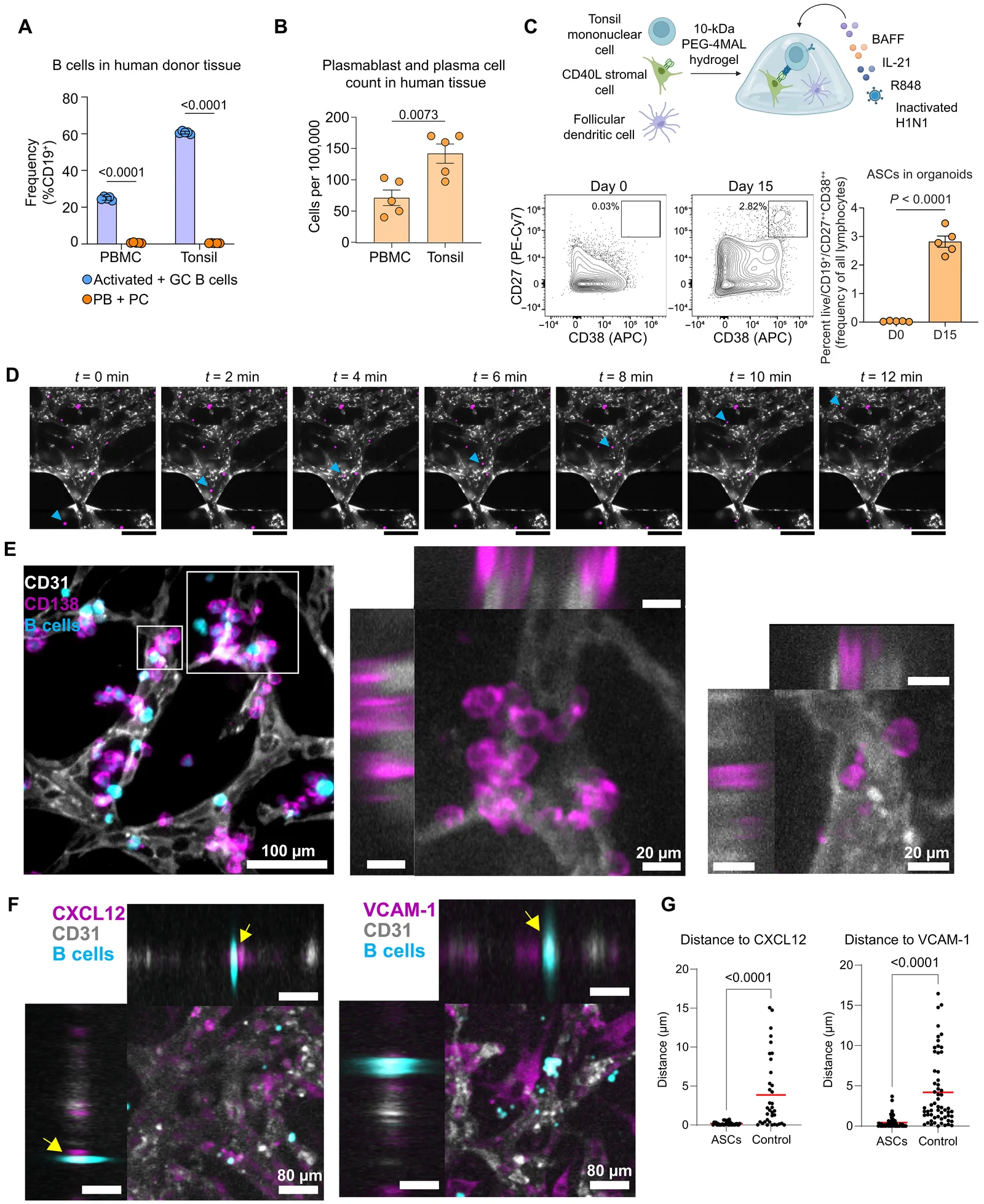
Loaded ASCs localize in the perivascular space of the BMOC. B cell subpopulations in human donor tissue are shown as percentages (**A**) and counts per 100,000 cells (**B**). (**C**) A schematic of lymphoid organoids displays the coculture of tonsil cells with CD40L and stromal cells in a PEG-4MAL synthetic hydrogel (top). Created in BioRender. L.K. (2024); https://BioRender.com/q40wo2f. Bottom: Representative ASC populations from day-0 tonsil samples and after 15 days of culture in the lymphoid organoids are illustrated. (**D**) Time-lapse microscopy captures loaded B cells (magenta) entering the central channel from the media channels and moving through the vessels (white), with a single cell tracked by a blue arrow. Scale bars, 500 μm. (**E**) Loaded B cells (cyan) aggregate near the vessels (white), with the scale bar measuring 100 μm. The zoomed areas highlight B cells outside (left) and inside (right) the vessels. (**F**) Representative Z-slices show B cell localization around niche markers: left: *YZ* view, top: *XZ* view, and center: *XY* view. Scale bars, 80 μm. Yellow arrows highlight B cells that are located near pink CXCL12 or VCAM-1 staining. (**G**) Distance of ASCs from CXCL12 and VCAM-1 compared to randomized control distribution using Welch’s unpaired *t* test.

A major challenge in studying human ASCs in hBMOC is the low abundance of these cells in blood and tonsils, whereas BM contains a higher number of differentiated PCs. ASCs make up only 0.01 to 1% of the total cell population in circulation and lymphoid tissues (*2*). Our observations indicate that although tonsil-resident ASCs outnumber circulating ASCs in PBMCs, tonsil-derived ASCs represent just 0.33 ± 0.02% of the total B cell population in freshly isolated tonsil mononuclear cells. This translates to roughly 140 cells per 100,000 freshly dissociated tonsil cells and only 70 ASCs per 100,000 PBMCs (Fig. 2, A and B). Because of the limited availability of ASCs, we used our recently established human immune organoid platform to differentiate primary B cells ex vivo, allowing for the gradual production of ASCs (*30*). Biomaterials-based lymphoid organoids, which mimic secondary lymphoid organs, provide a powerful platform to model adaptive immunity, enabling insights into lymphoid tissue development, immune responses, and disease pathogenesis in a physiologically relevant context (*30*, *52*–*55*). In our published work, we performed spatial multiomics and biophysical profiling of human lymphoid tissues to define the cellular architecture, receptor-ligand interactions, and mechanical properties that govern GC responses. Imaging mass cytometry and cyclic immunofluorescence mapped the spatial organization of B cells, T follicular helper cells (T_FH_ cells), and follicular dendritic cells (FDCs), whereas atomic force microscopy quantified physiologic tissue stiffness. We incorporated these biochemical and biophysical parameters into a synthetic hydrogels composed of maleimide (MAL)–functionalized polyethylene glycol (PEG) hydrogels (PEG-4MAL) (*30*, *52*, *53*, *56*–*58*), engineered to recapitulate the native lymphoid microenvironment. The ECM was reconstructed using collagen- and fibronectin-mimetic adhesive peptides (GFOGER and RGD), optimized to maximize GC B cell expansion and ASC generation. Matrix stiffness was tuned to ∼500 Pa, within the physiological range of human tonsil tissue, and combined with membrane-bound CD40L-expressing stromal mimics and human FDCs to reproduce key T cell–B cell and stromal interactions.

Our human immune organoids, derived from tonsils or PBMCs, show the formation of CD19^+^CD38^+^CD27^+^ GC B cells, CD19^+^CD38^hi^CD27^hi^ ASCs, and CD19^lo^CD138^+^ PCs, also displaying key characteristics of B cell programming (*30*). These organoids facilitate essential adaptive immune functions such as GC compartmentalization, somatic hypermutation, immunoglobulin class switching, and expansion of B cell clones (*30*). This platform established a controllable ex vivo human lymphoid organoid system for GC formation and high-yield ASC production.

On day 0, the organoids were exposed to inactivated H1N1 virus, serving as a model vaccine, along with Toll-like receptor 7/8 adjuvant R848, as previously described (*30*). The medium was refreshed every 2 days and supplemented with interleukin-21 (IL-21; Fig. 2C). In these immune organoids, the count of tonsil-derived CD19^+^CD38^hi^CD27^hi^ ASCs peaks at 12 days (*30*), whereas our current study identified a notable generation of ASCs on day 15 (Fig. 2C). At this stage, the organoids were digested with collagenase, and the harvested cells were enriched for CD19^+^ cells using magnetic-activated cell sorting before being transferred into the hBMOC through the vascular network.

In vivo, the BM connects to the blood circulation, necessitating that all immigrating cells traverse the BM vasculature (*59*). Consequently, we created BMOC so that the lumens of the central channel vasculature are directed outward through the gaps between the pillars, facilitating the introduction of ASCs via the media channel.

CXCL12 is the primary chemoattractant that directs PCs from the circulation to the BM and is produced by stromal cells(*59*). PCs express the CXCR4 receptor, which senses CXCL12 gradients and promotes their homing and migration to the BM (*60*). CXCR4 signaling activates VLA-4 integrins, facilitating rolling, adhesion, and arrest within the BM microvasculature (*61*). Fibronectin likely plays a crucial role in anchoring PCs to the BM ECM through its interaction with integrins, including VLA-4 (*21*, *62*). To replicate this procedure, we loaded tonsil organoid–derived ASCs into the hBMOC through the media channels. Using time-lapse imaging, we observed the ASCs entering the central channel hydrogel via the vessels (Fig. 2D). Notably, the ASCs exhibited a slowdown as they traversed through the vessels, ultimately becoming arrested. After 4 days of culture, the devices were fixed and imaged to assess ASC localization within the 3D BM niche. Our findings revealed that ASCs localized in the perivascular area, forming cell clusters around the vessels (Fig. 2E). This suggests potential extravasation of cells from the vascular network within the hBMOC device. This finding further establishes hBMOC in relation to in vivo BM conditions, as previous studies have shown that BM PCs preferentially localize to the perivascular niche, where they have opportunities to interact with various other cell types (*63*–*65*). The clustering of PCs, rather than a uniform distribution throughout the BM, is a feature that has also been observed in in vivo mouse studies (*66*).

### ASCs occupy perivascular niche in contact with hBMOC survival and adhesion ligands

The interactions between CXCL12 and CXCR4 coordinate the migration and homing of ASCs to the BM. Eliminating CXCR4 in mice leads to a decrease in PC numbers in the BM (*67*). In the BM, survival niches of ASC are organized by stromal cells that express CXCL12 (*68*). CD138^+^ ASCs in sections of mouse BM are in contact with CXCL12^+^ cells (*69*), as they exist in our human BMOC model, suggesting that CXCL12 continues to serve as a survival or retention factor (*70*–*72*). We hypothesized that the expression of these survival factors results from the perivascular positioning of ASCs, allowing them to stay close to vascular networks. To illustrate the proximity of CXCL12 to ASCs, we cultured membrane-labeled ASCs in hBMOCs and performed immunostaining for CXCL12 expression within the niche. Confocal imaging showed that the ASCs were located in the same *z* plane as the lectin-stained vessels and were adjacent to the CXCL12 signal, which was present on lectin^+^ endothelial cells (Fig. 2F).

To quantify the distance from ASCs to the CXCL12 signal, we generated maximum intensity projection images for ASCs and CXCL12. ASCs were segmented as discrete objects, and the CXCL12 signal was thresholded to generate a binary mask. Subsequently, a distance transform was applied to the CXCL12 mask, and the distance from each ASC centroid to the nearest CXCL12^+^ region was measured. These distances were converted to micrometers based on image calibration. The quantitative spatial analysis demonstrated that ASCs were positioned an average of 0.20 μm from the CXCL12^+^ signal, which was significantly less than expected under a random distribution (control), and were coplanar within *z* resolution (Fig. 2G and fig. S1).

Additional ligand pairs that promote ASC survival by keeping them within their niche include the adhesion ligand VCAM-1 and the adhesion receptor VLA-4 (*9*). In vitro studies investigating PC migration on different surface substrates revealed that cells arrest and tightly adhere to VCAM-1–coated surfaces, in contrast to intercellular adhesion molecule–1–coated surfaces (*66*). Our data indicated that VCAM-1 was present in the perivascular area of the hBMOC, with ASCs located near the VCAM-1 signal (Fig. 2F). Quantitative spatial analysis demonstrated that ASCs were located at an average distance of 0.42 μm from VCAM-1^+^ signal, again significantly closer than would be expected by random distribution (control), and they were also coplanar within the *z* resolution (Fig. 2G and fig. S1). Overall, imaging revealed that ASCs reside in the perivascular region of the BMOC, where they exist in vivo and establish strong interactions with CXCL12 and VCAM-1, key ligands crucial for the survival and retention of ASCs within their niche.

### BAFF and IL-21 promote ASC survival in the hBMOC

**Fig. 3.**
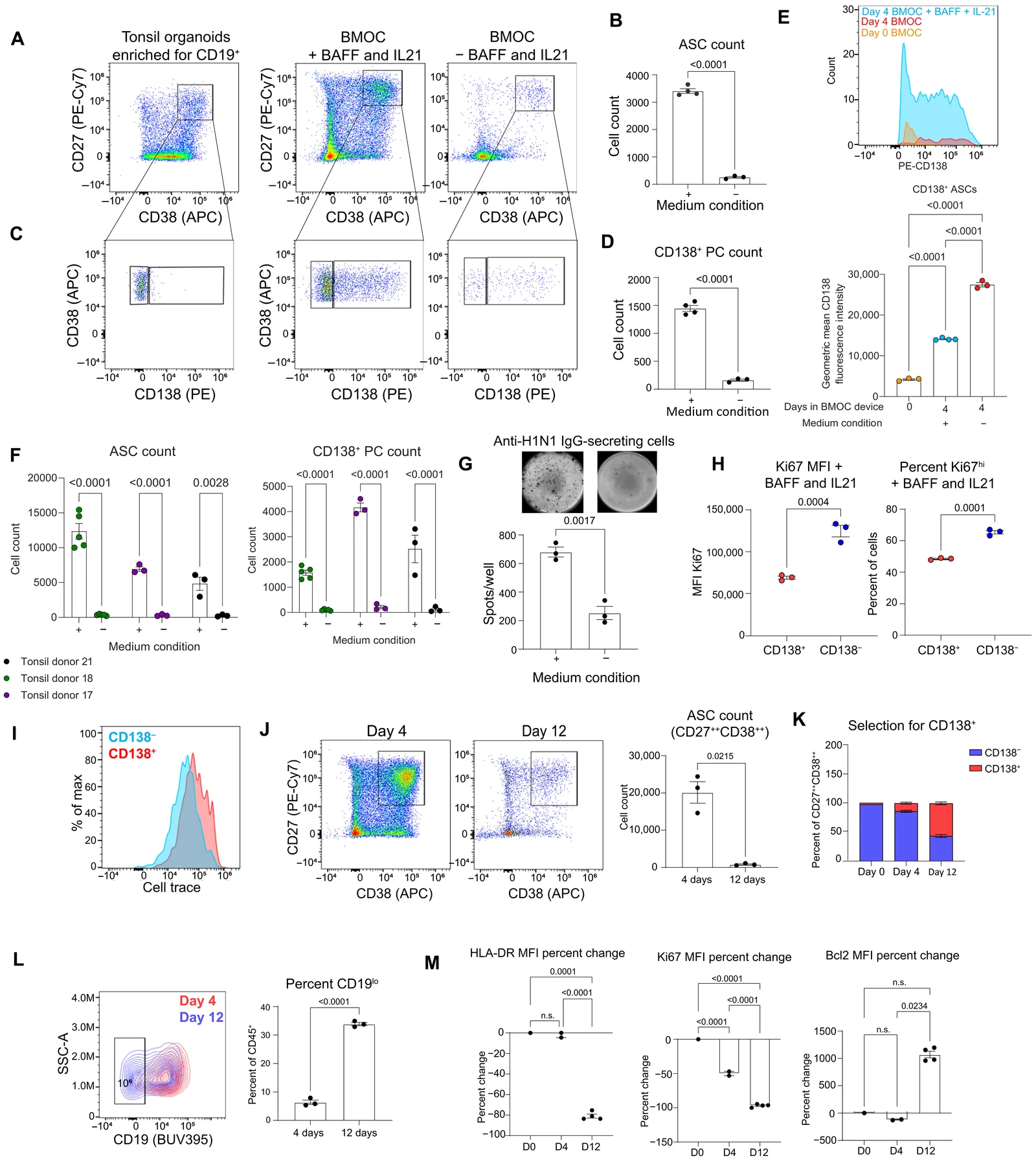
ASC survival in the hBMOC promoted by BAFF and IL-21. (**A**) Representative gating of ASCs from tonsil organoids (left) and after these cells were cultured in the hBMOC for 4 days (right). (**B**) Quantification of ASC counts in (A). (**C**) Representative gating of CD138^+^ ASCs from tonsil organoids (left) and after these cells were cultured in the hBMOC for 4 days (right). (**D**) Quantification of CD138^+^ PC counts in (C). (**E**) CD138 expression in ASCs. (**F**) ASC and CD138^+^ PC counts for various donors and cell sources. (**G**) ELISPOT quantification of anti-H1N1 immunoglobulin G (IgG)–secreting cells following 4 days of culture in the hBMOC. (**H**) Comparison of Ki67 expression in CD138^+^ and CD138^–^ ASCs. (**I**) Retention of CellTrace proliferation dye in CD138^+^ versus CD138^–^ ASCs. (**J**) Representative gating of ASCs from tonsil organoids after different culture durations in the hBMOC (left) and quantification of ASC counts (right). (**K**) Percentages of CD138^+^ and CD138^–^ ASCs at different culture lengths. (**L**) Representative gating of CD19^lo^ cells on days 4 and 12 of hBMOC culture (left) with quantification of the percentage of CD19^lo^ (right). (**M**) The expression of PC maturation markers is shown as the percent change from day-0 mean fluorescence intensity (MFI). Experiments had *N* = 3 to 4, presented as mean ± SEM; analyzed using one-way analysis of variance (ANOVA) with Tukey’s post hoc comparison or unpaired *t* test. n.s., not significant.

To assess the role of cytokines in B cell behavior within the hBMOC system, we investigated how the addition of BAFF and IL-21 affects CD19^+^ cells in the microfluidic devices. BAFF interacts with BAFF-R, BCMA, and TACI receptors on B cells and ASCs, promoting their survival and proliferation (*73*). IL-21 is linked to PC differentiation (*74*). Our study focused on a shorter time frame, as the aim was not to assess PC longevity but to investigate the initial positioning of ASC relative to human BM components. As a result, we observed the ASC response over the following 4 days. The ASCs persisted in the device for at least this period, and we recorded a 13-fold increase in ASC survival when the medium included BAFF (100 ng/ml) and IL-21 (10 ng/ml; Fig. 3, A and B, and fig. S2). This rise in ASCs could result from the generation of new ASCs (PC) from the seeded CD19^+^ cells in hBMOC or from the proliferation of existing ASCs.

To better understand this, we analyzed the expression of Syndecan-1 (CD138), a key surface marker of long-lived PCs (*14*). Previous research has shown that plasmablasts are generally CD138-negative or display low levels of CD138, whereas mature PCs are characterized by high CD138 expression (*75*). We observed that the number of tonsil-derived CD19^+^CD38^hi^CD27^hi^ ASCs that gained expression of CD138 during the 4 days of device culture was significantly higher than that of the initially loaded tonsil cells (Fig. 3C). This gain of CD138^+^ cells occurred in both media types, with and without BAFF and IL-21. However, the total count of CD138^+^ ASCs was 8.9-fold greater in the medium supplemented with BAFF and IL-21 (Fig. 3D).

In addition, we noted an increase in CD138 expression (Fig. 3E) in hBMOC devices, indicating that ASCs were undergoing further differentiation in hBMOCs. This could be linked to the transition from early minted CD138^–^ ASCs (plasmablasts) to mature CD138^+^ ASCs or PCs (*9*). The increase in ASCs and CD138^+^ cells was consistent among various tonsil donors when BAFF and IL-21 were present in the medium (Fig. 3F). We conducted an ELISPOT assay to assess ASC function by quantifying anti-H1N1 immunoglobulin G (IgG) ASCs obtained from the devices. We found that supplementing the medium with BAFF and IL-21 led to a threefold increase in anti-H1N1 IgG ASCs compared to medium without these cytokines (Fig. 3G). Furthermore, we noted that the CD138^+^ ASCs exhibited lower Ki67 expression and a reduced fraction of Ki67^hi^ cells compared to the CD138^–^ cells, indicating that the more mature CD138^+^ cells in hBMOC were less proliferative (Fig. 3, H and I), which is characteristic of mature ASCs in vivo.

### ASCs mature during extended culture in the hBMOC

To assess the impact of culturing ASCs in hBMOC for an extended period, we prolonged the culture duration in hBMOC to 12 days and retrieved ASCs from the devices. At this more extended culture point, the number of ASCs found in hBMOC was lower than on day 4 (Fig. 3J), yet the proportion of CD138^+^ increased over time, rising from 2% on day 0 to 56 ± 4% by day 12, compared to 14 ± 3% on day 4 (Fig. 3K). The decrease in ASC numbers could result from the intrinsic characteristics of ASCs, which undergo rapid apoptosis in vitro, or from inadequate conditions such as hypoxia (*14*). However, long-term survival was not the objective of our study. The simultaneous decrease in ASC count concurrently with the increase in CD138^+^ fraction suggests that, despite cellular apoptosis, there is a niche-mediated enrichment of CD138^+^ cells. CD138 plays a role in cell-matrix adhesion, anchoring the cells to the BM niche and enhancing ASC survival by attaching to prosurvival factor cytokines (*76*–*78*). Thus, expression of CD138 in mature ASC offers a selective survival benefit compared to less mature CD138^–^ cells, a phenomenon observed in mouse models (*76*) and human cell-free systems (*79*), and is replicated in the hBMOC. We also observed that the ASCs were developing in the BMOC, indicated by the emergence of a CD19^lo^ population. On day 4, the CD19^lo^ fraction was 6.4% ± 0.79, which significantly increased to 33.9 ± 0.58% by day 12 (Fig. 3L). In addition, we noted that the ASCs gradually lost HLA-DR and Ki67 expression, whereas they began to express Bcl-2 over time (Fig. 3M), all of which are indicators of PCs differentiation (*40*, *80*, *81*).

### Extravasated ASCs survive in the hBMOC

**Fig. 4.**
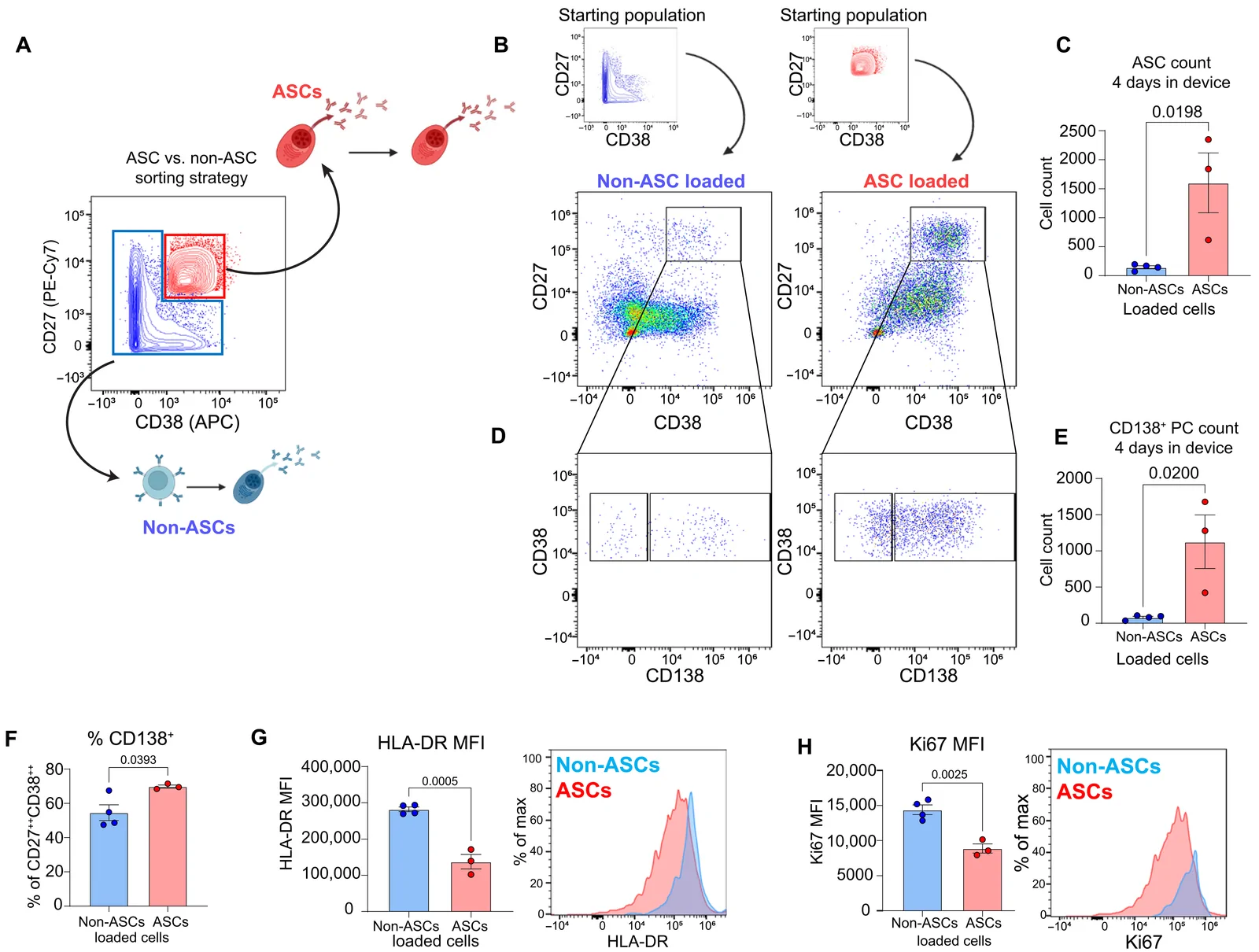
Comparison of sorted organoid subpopulations in the hBMOC. (**A**) Strategy for sorting tonsil organoid cells via FACS into ASC and non-ASC subpopulations. (**B**) Representative gating of ASCs post 4 days in the BMOC for either ASCs or non-ASCs. (**C**) Quantification of ASC counts from (B). (**D**) Representative gating of CD138^+^ ASCs following 4 days of culture in the BMOC. (**E**) Quantification of CD138^+^ PC counts from (D). (**F** to **H**) Assessment of PC maturation marker expression after BMOC culture among various initially loaded cell populations. Experiments were performed with *N* = 3 to 4, expressed as mean ± SEM; unpaired *t* test.

In previous experiments, we transferred all CD19^+^ cells from the human lymphoid organoids into the hBMOC system. Because only a subset of these organoid-derived cells are ASCs (Fig. 4A), we aimed to determine whether the observed cellular responses were due to ASCs rather than other CD19^+^ populations introduced into the device. Specifically, we explored whether the reactions resulted from the proliferation and differentiation of non-ASCs into ASCs instead of the maintenance and maturation of preexisting ASCs. These experiments were conducted in the presence of IL-21 and BAFF.

We used fluorescence-activated cell sorting (FACS) to obtain pure populations of CD27^++^CD38^++^ ASCs and non-ASCs. These purified cell populations were loaded into separate devices and analyzed by flow cytometry on day 4. Our findings suggest that more ASCs are recovered from devices initially loaded with ASCs, with a higher percentage of these cells expressing CD138^+^ (Fig. 4, B to E). In addition, the CD27^++^CD38^++^ cells from the ASC-loaded condition displayed more mature characteristics than those derived from non-ASCs, as evidenced by lower expression of HLA-DR and Ki67 (Fig. 4F). Previous research indicates that HLA class II expression distinctly differentiates early and late BM ASC subsets. As early ASCs transition into resting, long-lived PCs, they generally lose major histocompatibility complex (MHC) class II (MHCII) genes (*40*, *79*, *82*). These findings indicate that in hBMOC, the ASCs are maturing instead of non-ASCs differentiating into new ASCs.

### Endosteal and perivascular compartments of the BM niche contribute to ASC motility and abundance

**Fig. 5.**
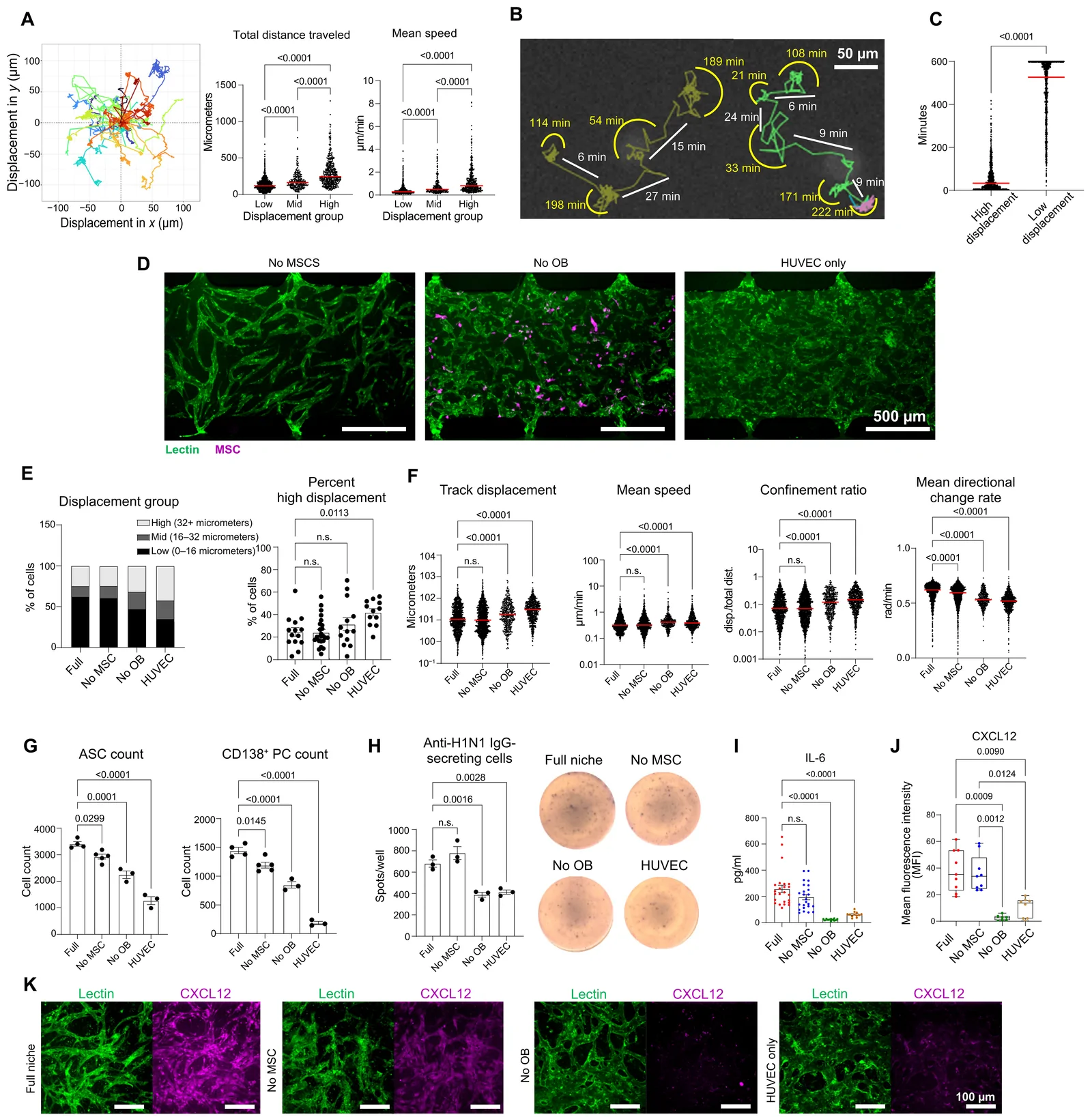
BM niche modulates motility of ASCs. (**A**) Plotted 2D tracks of ASCs with the same origin (left) and motility metrics of the cell tracks divided into categories based on track displacement (right). (**B**) Examples of ASC tracks exhibiting periods of both high and low displacement periods. Scale bar, 50 μm. (**C**) Comparison of the total time each cell spent in high versus low displacement periods. (**D**) Epifluorescence images capturing niche variations. Scale bars, 800 μm. The comparable image of the full niche can be referred to in Fig. 1E. (**E**) Percentage of cells in each motility category based on displacement (left) and the proportion of highly motile cells in each device group (right). (**F**) Track displacement, average speed, confinement ratio, and mean directional change of membrane-labeled B cells in devices with varied niche types. Kruskal-Wallis test with Dunn’s post hoc analysis; *N* = 1800 total cells collected from 14 regions of interests (ROIs) across five devices. (**G**) Counts of ASC (left) and CD138^+^ (right) after being cultured in the hBMOC for 4 days under differing niche conditions. *N* = 3. (**H**) ELISPOT analysis of anti-H1N1 IgG-secreting cells post 4 days of culture in the hBMOC under varying niche conditions with *N* = 4. (**I**) Analyzed concentrations of secreted IL-6 from the medium of the devices using one-way ANOVA with Tukey’s post hoc comparison. (**J**) Quantification of MFI of CXCL12 signal in confocal images of hBMOC. One-way ANOVA with Tukey’s post hoc analysis; *N* = 7 to 9 ROIs from across three devices. (**K**) Representative confocal projections of hBMOC stained for vasculature (green) and CXCL12 (magenta). Scale bars, 200 μm.

To gain insight into the migration dynamics of the ASCs within the BM niche, we imaged the central channel of devices containing membrane-labeled CD19^+^ cells from tonsil organoids every 3 min over a duration of 10 hours. Single-cell tracking indicated that a portion of these cells exhibited motility within the device (Fig. 5A), with 25 ± 3.9% of the cells being displaced by at least 32 μm, qualifying them as highly motile. On average, the cells traveled at a mean speed of 0.45 μm/min, which is 50% of the speed observed in previous mouse studies indicated (*66*).

Earlier studies on mouse intravital imaging suggest that PCs exhibit intermittent and heterogeneous motility in the BM (*66*). It is theorized that PC motility within the BM allows them to compete for access to limited survival niches. Previous studies using intravital imaging in mice indicate that plasma cells exhibit intermittent and heterogeneous motility within the BM (*66*, *72*, *83*). These intermittent periods of motility in the BM enable PCs to locate and occupy survival niches (*66*, *72*, *83*). Characterized by a “stop-and-go” motility pattern, the PCs predominantly remained stationary, interspersed with brief migratory phases (*66*, *72*, *83*). This movement pattern seems to be exclusive to BM PCs, as it has not been observed in lymphoid tissue or the spleen (*66*, *72*, *83*). In addition, plasmablasts are reported to move faster in the lymph node than they do in the BM (*66*, *72*, *83*). Cells in hBMOC displayed a similar movement pattern characterized by alternating periods of high and low displacement (Fig. 5B and movie S1). We identified these displacement intervals by applying a rolling average of velocity over 3-min intervals. Most cells exhibited mixed migration behaviors, showing both high and low displacement rates. On average, cells experienced high-velocity periods lasting 59 ± 2.4 min and low-velocity periods lasting 461 ± 4.8 min. This suggests that human ASCs spend more time held in a spot in the BM niche than moving to nearby neighborhoods (Fig. 5, B and C).

Next, we analyzed how the components of the BM niche affect ASC motility and survival. The hBMOC system’s modularity offers a selective advantage, allowing for the addition or removal of various cell types from the culture device. Consequently, we cultured tonsil organoid–derived CD19^+^ cells in four variations of the hBMOC niche: (i) hydrogel-encapsulated human umbilical vein endothelial cells (HUVECs) and MSCs without the underlying endosteal monolayer (no OB), (ii) devices excluding MSCs alongside the HUVECs in the hydrogel (no MSC), (iii) hydrogel containing only HUVECs (HUVEC), and (iv) the full BMOC system (full) (Fig. 5D).

Our observations indicate that the absence of the endosteal layer enhanced cell motility. The percentage of highly motile cells increased from 25 ± 3.9% (full) to 32 ± 5.7% (no OB) and 42 ± 2.6% (HUVEC) when the endosteal layer was removed (Fig. 5E). Furthermore, the presence of the endosteal niche was associated with a reduction in both the number of motile cells and their mean speed and total displacement. This indicates that, in the absence of the niche, individual cells demonstrated increased motility and migrated further from their initial positions (Fig. 5F). This is supported by an increased confinement ratio under the no OB and HUVEC-only conditions (Fig. 5F), which measures the efficiency of cells in distancing themselves from their starting point (displacement/total distance traveled).

Whereas cells migrated farther from their initial position without the endosteal layer, there was no significant difference in the total distance covered among the groups. This implies that the motile cells within the whole niche and under no MSC conditions were active but restricted to a limited back-and-forth movement. We computed the average directional change rate and found that it was higher under control and no MSC conditions, indicating that these cells were more confined and turned frequently (Fig. 5F). Overall, these data highlight the crucial role of endosteal niche components in regulating ASC motility within BM.

Alongside ASC motility, we identified how individual niche components affect ASC survival. We noted that the ASCs and CD138^+^ ASCs declined when cell components were excluded from the device (Fig. 5G). Devices lacking perivascular MSCs supported 1.2 times fewer ASCs than complete devices, whereas devices missing the endosteal layer supported 1.5 times fewer ASCs. HUVEC-only devices had the fewest recovered ASCs, with a 2.7-fold reduction. Notably, although MSC removal did not influence the number of anti-H1N1 IgG-secreting cells, the absence of the endosteal layer significantly reduced the count of spot-producing cells in the hBMOCs (Fig. 5H). We performed a cytokine analysis of media sampled from devices with varying niches and found that the IL-6 concentration decreased markedly in the absence of the endosteal layer (Fig. 5I). The impact of the endosteal layer may be linked to the combined expression of CXCL12, VCAM-1, and the release of other factors, warranting further in-depth exploration.

We hypothesized that the endosteal layer contributes factors within the BM niche that enhance ASC survival and keep the cells localized in that niche. In contrast, removing the endosteal layer reduced survival rates and allowed ASCs to migrate farther. We performed confocal imaging of four niche variations in hBMOC to assess the expression of CXCL12, a chemokine known for its roles in ASC homing and retention. Our findings indicated that CXCL12 expression was detected in both complete devices and ones without MSCs. Nevertheless, CXCL12 expression was not detectable under either condition devoid of the endosteal layer (Fig. 5, J and K, and fig. S3) (*66*). Collectively, these modifications to the components of the BM niche likely altered the dynamics of the ASCs.

### CXCL12-CXCR4 signaling controls ASC motility in human BMOC

**Fig. 6.**
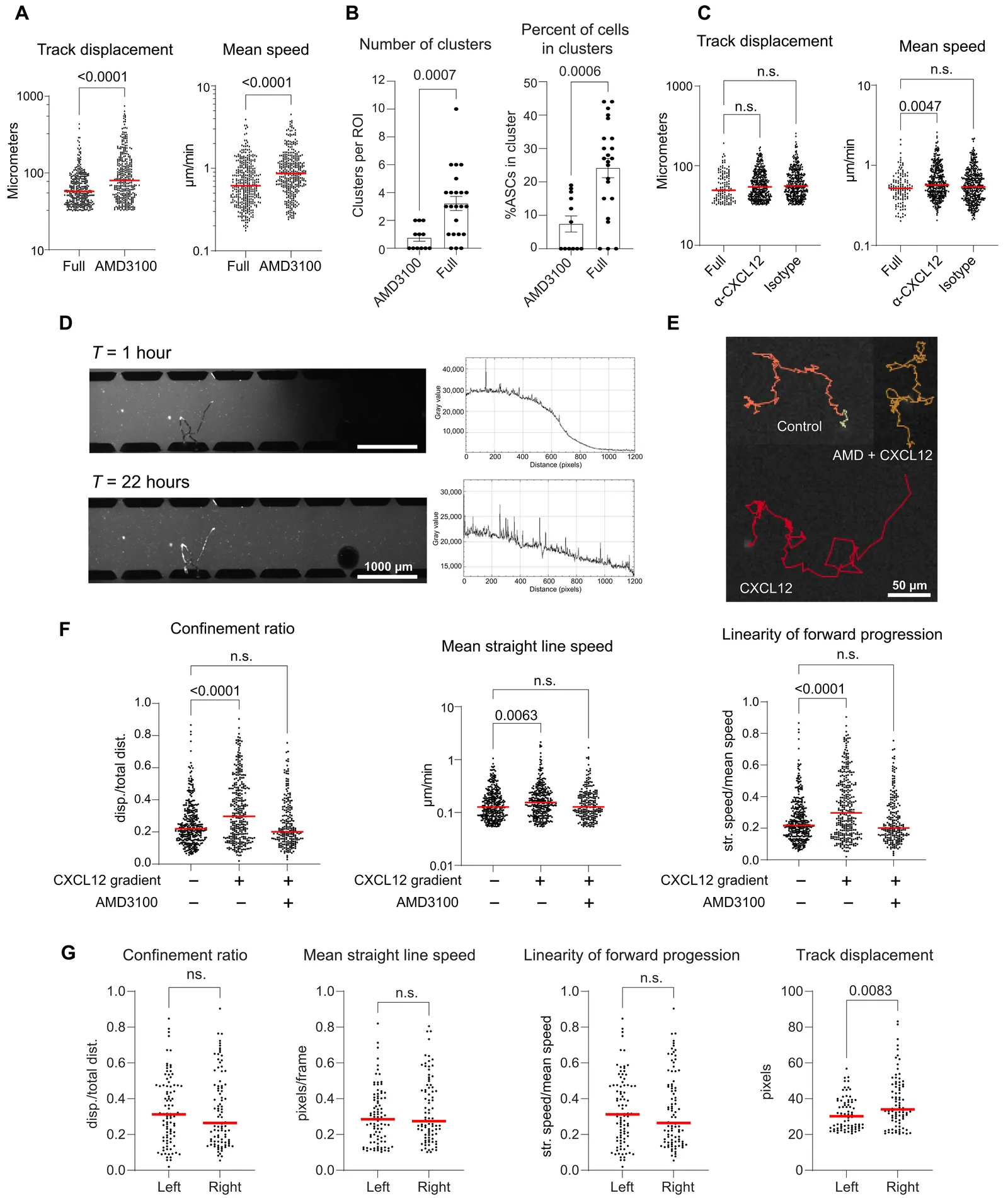
CXCL12-CXCR4 signaling controls ASC motility. (**A**) Pooled measurement of ASC motility with or without AMD3100 treatment. (**B**) Clustering analysis of ASCs within hBMOC with or without AMD3100 treatment. (**C**) Pooled measurement of ASC motility with or without α-CXCL12 treatment. (**D**) Epifluorescence images displaying a 10-kDa fluorescent dextran gradient in the hBMOC (left) and quantifying the fluorescent intensity (right). (**E**) Samples of ASC tracks under various treatment conditions. Scale bar, 50 μm. (**F**) Mobility metrics of loaded B cells in the hBMOC. The devices featured a CXCL12 gradient and were treated with the AMD3100 CXCR4 inhibitor. A Kruskal-Wallis test with Dunn’s post hoc was applied. *N* = 1800 total cells from 14 ROIs across five devices. (**G**) Comparison of ASC motility in left (high CXCL12) and right (low CXCL12) sides of hBMOC devices. *N* = 90 (left) and *N* = 87 (right) cells from seven devices.

We hypothesized that the reduction of CXCL12 expression partially contributes to motility alterations under no-endosteal conditions, owing to its role in regulating ASC homing and retention. Consequently, we disrupted CXCL12-CXCR4 signaling using AMD3100, a CXCR4 antagonist, to assess whether CXCL12 regulates ASC dynamics in the hBMOC. Following the administration of AMD3100, ASC motility increased, reflected by track displacement and average speed (Fig. 6A). Furthermore, the administration of AMD3100 resulted in a reduction of ASC clustering within the niche. Using the density-based spatial clustering of applications with noise algorithm, we identified regions of high ASC density within the hBMOC. Approximately 24 ± 2.9% of ASCs were classified as clustered, with an average of about 3.2 clusters per 1.96 mm–by–1.45 mm field of view. Conversely, devices treated with AMD3100 exhibited a significant decrease in the number of clusters per image, with only 7.4 ± 2.4% of ASCs residing within clusters (Fig. 6B and fig. S4). To confirm the effects of CXCR4 blockade on ASC motility, we evaluated its ligand, CXCL12. The devices received treatment with either a blocking anti-CXCL12 antibody or an isotype control (*84*, *85*), followed by imaging. Post treatment with anti-CXCL12, ASCs exhibited increased motility in comparison to isotype-treated controls, as indicated by average speed (Fig. 6C). These findings suggest that CXCR4-CXCL12 signaling plays a vital role in regulating ASC migration within the hBMOC. Notably, the absence of CXCR4-CXCL12 signaling correlates with increased motility of ASCs. This observation was unexpected, as CXCL12 is traditionally recognized as a chemokine that facilitates cell migration (*83*); however, our results indicate that ASCs exhibit greater motility in the absence of CXCL12 present (*83*). However, our findings are aligned with in vivo evidence that AMD3100 prompts various leukocyte subsets, such as PCs, to exit from the BM to the blood (*66*). AMD3100 treatment in mice has been shown to reduce PC and B cell subsets in the BM by two- to threefold while raising cell counts in the blood threefold (*71*, *86*, *87*).

To further investigate this phenomenon, we examined the spatial distribution of CXCL12 within the hBMOC. We observed consistent CXCL12 expression throughout the central channel of the hBMOC rather than distinct regions of high and low expression. We therefore hypothesized that when CXCL12 is uniformly expressed, it provides an anchoring effect, acting as a retention factor that keeps cells stationary rather than directional guidance. Meanwhile, blocking CXCL12 causes cells to be released from their initial locations. To test whether introducing spatial asymmetry would shift CXCL12 function toward chemotaxis, we engineered a CXCL12 gradient by introducing 100 ng/ml CXCL12α into the left media port of each media channel. This setup produced a well-defined gradient in the central channel, maintained for up to 22 hours (Fig. 6D). We analyzed the cell movement of highly motile cells in devices with and without a CXCL12 gradient and observed enhanced motility along with variations in migration patterns. The data indicated a higher confinement ratio under the CXCL12 gradient condition, implying that cells were more “efficient” in moving away from their initial positions (Fig. 6, E and F). In addition, we assessed the mean straight-line speed and linearity of forward progression (LFP), calculated as mean straight-line speed divided by mean speed. The results showed increased mean speed and LFP of motile cells in devices featuring CXCL12 gradients (Fig. 6F). To assess the impact of CXCL12 concentration on ASC motility, we performed the same analysis after splitting the imaging data into those from the left side (high concentration region) and the right side (low concentration region) of the central channel. Upon subdividing our dataset, we found that cells maintain elevated confinement ratios, mean speeds, and LFPs across regions.

However, our data reveal that ASCs on the right side of the device and therefore exposed to a lower concentration of CXCL12 exhibit increased track displacement (Fig. 6G). This trend aligns with our findings using the CXCR4-CXCL12 signaling inhibitors AMD3100 and α-CXCL12, further suggesting that CXCL12 signaling, or, in this case, increased CXCL12 concentration, is required for ASC retention within their microniche. Following this, we treated the CXCL12 gradient devices with AMD3100 and found that this treatment prevented the cells from responding to the CXCL12 gradient. As a result, the confinement ratio, mean speed, and LFP returned to baseline levels, showing no significant difference compared with control devices (Fig. 6F). Here, our findings are corroborated by previous chemotaxis studies in which ASCs migrate in vitro toward a CXCL12 gradient, and this migration is prevented with AMD3100 pretreatment (*66*, *71*).

## DISCUSSION

Using the hBMOC platform and integrating ASCs from human immune organoids, we explored the maturation, spatial organization, and motility of human ASCs within the BM niche. Our findings indicate that the B cells sourced from the tonsil cells can be activated and differentiated through a GC reaction, resulting in the formation of ASCs that can subsequently be introduced into the hBMOC. These ASCs aggregate in clusters within the perivascular area and are positioned in proximity to various adhesion molecules and cytokines known to govern ASC survival and retention within the BM, such as CXCL12 and VCAM-1. Secreted CXCL12 is immobilized in the ECM through binding to proteoglycans, namely, heparan sulfates, which are found predominantly in the basement membrane of the BMOC vascular network (*12*, *37*, *46*–*48*, *88*–*90*). These ASCs can persist in the 3D BM niche for up to 12 days, gradually maturing into a phenotype resembling that of PCs. We validated the necessity of diverse BM niche cell types in supporting the ASCs. Our study reveals that a subset of ASCs exhibits motility, displaying a movement pattern akin to the stop-and-go behavior observed in mice, with this motility being partially regulated by CXCR4-CXCL12 signaling.

The organization, survival, and migration of ASCs in the BM are currently not well understood in a human setting, largely because techniques for time-resolved imaging of human ASCs in their native environment are not practically feasible. Hence, several of our comparisons necessarily rely on murine studies; accordingly, our findings should be interpreted as establishing a human experimental framework that both contextualizes prior mouse work and enables rigorous testing of species-specific differences moving forward. Our platform provides an unprecedented opportunity to study human ASCs within the human BM niche, but this also means limited existing data for direct comparison or validation with human samples. Much of our understanding is based on mouse studies; although human and mouse PCs share many similarities, we cannot assume their behavior is identical across species. In both mice and humans, plasma cell development and function are regulated by the same transcription factors (such as BLIMP1 and interferon regulatory factor 4), and PCs down-regulate common B cell markers (such as B220, MHCII, and Bcl6). In addition, in both species, PCs migrate to the BM guided by CXCL12 and express key receptors such as CXCR4, BCMA, and IL-6 receptor for homing and survival within the BM niche. Nevertheless, important differences between the species should be noted (*4*, *40*, *41*). Human PCs exhibit greater heterogeneity, with several distinct functional subsets identified. Differences in BM architecture are also evident: Comparative studies between human and mouse BM show that mouse BM has higher cellularity, more megakaryocytes, and fewer adipocytes. These architectural differences may suggest that the signals supporting PC survival are not identical across species. Still, it is unclear whether factors such as adipogenicity influence PC biology. Ideally, future research will include direct comparisons between the hBMOC and human BM architecture (*4*, *40*, *41*).

Other limitations that can be addressed in future iterations of the platform’s development include the lack of control over hypoxia and the longevity of the model for extended-duration experiments. The BM is inherently hypoxic compared to other organs, with an oxygen tension ranging from ∼1 to 6% or less than 10 mmHg (*2*, *14*, *91*). This hypoxic environment is considered essential for the ASC survival niche, with in vitro studies showing that human ASCs survive better under hypoxic conditions (*14*). However, our experiments were conducted under standard cell culture conditions of ∼20% oxygen, and we have no evidence of hypoxia occurring within the hBMOC. Future studies might include using a modular incubator chamber that can be set to specific oxygen levels or redesigning the hBMOC hardware to integrate electrochemical sensors that monitor hypoxia levels within the chip.

Our results indicate that ASCs survive and mature for up to 12 days in hBMOC. However, we observe increasing overgrowth of microvascular networks over time, and beyond this period, the networks become unstable. The deterioration of the BM by days 10 to 12 during ASC culture restricts our capacity to use this system for studying long-lived PC maintenance or extending culture duration beyond 50 days, as demonstrated in 2D human models (*14*). The development of this hBMOC platform offers tremendous potential for both basic and applied immunology. It provides an advanced system for studying human PCs, which are essential for long-term humoral immunity but remain poorly understood. For example, the platform could help investigate why some vaccines offer prolonged protection by simulating how various vaccination methods affect the development and longevity of long-lived PCs. In addition, by incorporating patient-derived cells, it can model BM diseases such as multiple myeloma and autoimmune disorders, facilitating personalized drug testing and the development of new treatments to address therapy resistance, including in cancer immunotherapy. Its modular design also makes it ideal for drug discovery and screening, enabling testing of new compounds that target the BM environment to improve ASC survival or control immune cell movement.

## MATERIALS AND METHODS

### Ethics oversight

The research presented here adheres to the ethical standards outlined by the Emory University School of Medicine’s Institutional Review Board (IRB)–approved protocol IRB00058507 for the collection of human tonsils.

### Experimental design

This study focused on investigating the behavior of human ASCs within an ex vivo BM model. We developed human BM-on-a-chip devices by assembling the required components and culturing target cell types to replicate the BM environment. Simultaneously, human ASCs were produced using lymphoid organoids and added to the BM-on-a-chip under different culture conditions. We subsequently collected and analyzed data through immunofluorescence microscopy, flow cytometry, and cytokine analysis.

### Plate and device fabrication

The devices are fabricated using a protocol akin to what was previously outlined (*28*, *92*). The 6-inch (15.24-cm) wafer for device fabrication was sourced from MuWells Inc. A cast from the patterned, silanized wafer was created using PDMS to form a “master mold.” This PDMS was cut to size and treated with vapor phase deposition of trichloro(1*H*,1*H*,2*H*,2*H*)-perfluorooctyl silane, promoting easy release from the epoxy molding material. Subsequently, a mixture of EpoxAcast 670 HT and Epic Epoxy thinner (Reynolds Advanced Materials) was prepared at a ratio of 100 base:17.6 cross-linker:5 thinner by weight and then poured over the silanized master mold. The mixture degassed under vacuum for 15 min and was cured at room temperature for 15 hours. At this stage, the mold was detached from the PDMS master and allowed to fully cure overnight at 65°C, followed by hard baking at 120°C for 3 hours. After cooling, the mold received treatment with vapor phase deposition of trichloro(1*H*,1*H*,2*H*,2*H*)-perfluorooctyl silane to prevent PDMS adhesion.

The PDMS was mixed in a 10:1 ratio (elastomer base to curing agent) by weight, cast onto the EpoxAcast mold, and cured at 65°C. The PDMS layer featuring the designs was extracted from the mold, and loading ports were created using a 1-mm biopsy punch. The feature layer of PDMS was then attached to a bottomless 96-well plate (Greiner Bio-One) using a chemical adhesive method (*93*). In summary, the 96-well plate was soaked in methanol containing 2% (v/v) 3-mercaptopropyl trimethoxysilane (Sigma-Aldrich) to add silane functional groups to the polystyrene. The excess was rinsed thoroughly with deionized water, and the plates were completely dried. The treated plates were then plasma bonded to the PDMS features using a plasma cleaner, followed by an incubation at 65°C overnight to complete the bonding process. After washing the plates with 70% ethanol, commercially available silicone film HT-6240 (BISCO) was attached to the bottom surface to seal the PDMS layer, using the same plasma cleaning method described earlier.

Before use in cell culture, the devices were sterilized with 70% ethanol and ultraviolet light, and the central channel PDMS was treated using a 0.1% dopamine HCl solution (Sigma-Aldrich) along with 20 μg/ml of sterile collagen I (Corning) to facilitate cell adhesion.

### Maintenance of cell types outside of devices

Human BM-derived MSCs (RoosterBio; donor 183) were expanded and cultured on tissue culture polystyrene (TCPS) using αMEM from Sigma-Aldrich, supplemented with 10% fetal bovine serum (FBS; Hyclone) and 1% penicillin-streptomycin (Hyclone), and were used until passage 6. These cells were used for both the formation of the endosteal layer and within the vascular network. HUVECs from pooled donors (Lonza) were cultured in microvascular endothelial growth medium (EGM-2 MV) (Lonza) on 0.1% bovine gelatin (Sigma-Aldrich)–coated TCPS and were used until passage 8. NHLFs (Lonza) were cultured in fibroblast growth medium-2 (Lonza) on TCPS and used up to passage 8. All cells were harvested using TrypLE (Gibco).

### Osteogenesis in the hBMOC

The central channels of the devices were seeded with MSCs at a density of 6 × 10^5^ cells/ml, using 5 μl per device, which amounts to 3000 cells each. After 1.5 hours of incubation to allow the cells to adhere to the surface of the channel, 200 μl of medium was added to the central channel media ports. To encourage osteogenesis and mineralization, these cells were cultured in the devices for 21 days in 300 μl of αMEM osteogenic medium. This medium contained 10% FBS, 1% penicillin-streptomycin, 10 mM β-glycerophosphate (Sigma-Aldrich), 50 μM ascorbic acid (Sigma-Aldrich), and 100 nM dexamethasone (Sigma-Aldrich), with media changes made twice a week.

### Vasculogenesis in the hBMOC

The central gel channel successfully underwent vasculogenesis using established methods (*94*, *95*). Briefly, HUVECs (4.2 × 10^6^ cells/ml) and MSCs (6 × 10^5^ cells/ml) were suspended in EGM-2 MV supplemented with thrombin (4 U/ml) (Sigma-Aldrich). A solution of fibrinogen (8 mg/ml) (Sigma-Aldrich) and collagen I (2 mg/ml) (Corning) was mixed thoroughly with the cell solution (1:1) and loaded into the central channel on top of the endosteal layer. For this process, the medium is first removed from the central channel media ports, and residual medium within the channel is displaced by the injection of the hydrogel solution. NHLFs (6 × 10^6^ cells/ml) were also suspended in EGM-2 MV supplemented with thrombin (4 U/ml), mixed 1:1 with a fibrinogen solution (6 mg/ml), and loaded into the two outer channels. When experimentally necessary, the MSCs were first membrane labeled with CellVue Claret Far Red Fluorescent Membrane Dye (Sigma-Aldrich). The devices that leaked during the loading process were not used for experimentation.

The devices were incubated for 15 min to facilitate the formation of the fibrin gel. EGM-2 MV containing vascular endothelial growth factor (VEGF; 50 ng/ml) (PeptroTech) was introduced into reservoirs on either side of the gel channel and drawn through the media channel. On the second day, the medium was replaced with EGM-2 MV that included both VEGF (50 ng/ml) and ANG-1 (100 ng/ml) (Peprotech). If needed for experiments, the medium was enhanced with DyLight 488–tagged *Lycopersicon Esculentum* Tomato lectin (2.5 μg/ml; Vector Laboratories) to visualize the vasculature.

Devices designed with modified niches were created by omitting the MSCs and/or applying the gels onto plates that had not completed 21 days of osteogenesis. The 2D endosteal layer offers vasculogenic factors. Therefore, in devices lacking this layer, it was essential to use the HUVECs at a concentration of 13.1 × 10^6^ cells/ml to facilitate vessel formation.

### Generation and culture of human lymphoid organoids

Human lymphoid organoids were produced as described previously (*30*). In summary, organoids were generated using synthetic hydrogels composed of 10-kDa PEG-4MAL (*30*, *52*, *53*, *96*) (Laysan Bio) at a macromer concentration of 7.5% (w/v). Initially, PEG-4MAL was functionalized at pH 7.4 and 37°C with two thiolated adhesive peptides: 2.7 mM RGD (a fibronectin/vitronectin mimic) and 1 mM GFOGER (a collagen I mimic) for 30 min (both custom sourced from AAPPTec). The protease-sensitive cross-linker VPM (AAPPTec) and the nondegradable cross-linker dithiothreitol (Thermo Fisher Scientific) were mixed in a 1:1 molar ratio and pH adjusted to 6. Before mixing the polymer with the cross-linker, the cell mixtures were suspended in the cross-linker solution. Tonsil mononuclear cells were sourced from human tonsils and adhered to the ethical guidelines established by Emory University School of Medicine’s IRB-approved protocol IRB00058507. Briefly, fresh tonsil tissue was mechanically minced into small fragments, passed through a cell strainer to obtain a single-cell suspension, and subjected to red blood cell lysis, as needed, and tonsil mononuclear cells were isolated using via density gradient centrifugation using Ficoll-Paque. For the fabrication of tonsil organoids, the cell mixtures contained 200,000 tonsil mononuclear cells, 40,000 CD40LL stromal cells (*30*, *53*), and 40,000 HK FDCs (Cytion) (*30*). The PBMC organoids were composed of 400,000 PBMCs, 40,000 CD40LL cells, and 40,000 HK cells (*30*). PBMCs were obtained commercially from StemCell Technologies.

Hydrogel-based organoids were grown in RPMI 1640 (Thermo Fisher Scientific) with 10% FBS, NEAA (Thermo Fisher Scientific), sodium pyruvate (Thermo Fisher Scientific), penicillin-streptomycin (Thermo Fisher Scientific), 2-mercaptoethanol (Thermo Fisher Scientific), human BAFF (100 ng/ml; R&D Systems), and human IL-4 (20 ng/ml; Peprotech) during the first 2 days (*30*). On the second day, IL-4 was switched to human IL-21 (10 ng/ml; Peprotech) for the remainder of the culture. The medium was renewed every 2 days. On day two, the organoids received a challenge with inactivated New Caledonia 20/99 (H1N1) influenza A virus (10 μg/ml; Bio-Rad) and 0.5 μM R848 (MedChemExpress), both diluted in a culture medium (*30*).

### Culture of organoid-derived B cells in the hBMOC

After the specified culture duration, organoids were enzymatically digested with type I collagenase (125 U/ml; Worthington Biochemical) for 1 hour at 37°C. A buffer containing serum was introduced at the end of this period to halt enzymatic activity. Organoid debris was eliminated using 96-well MultiScreen Mesh Filter Plates (EMD Millipore). The resultant cells were then prepared for loading into the hBMOC.

In most experiments, the entire CD19^+^ organoid cells were used for device culture. The degraded organoids were pooled and enriched for CD19^+^ cells using the EasySep Release Human CD19 Positive Selection Kit (StemCell Technologies) with twice the concentration of CD19 Positive Selection Cocktail and Releasable RapidSpheres as recommended by the manufacturer. For experiments requiring purified cell populations, the degraded organoid cells were stained with a 1:500 dilution of Fixable Blue (Thermo Fisher Scientific) viability dye, followed by a 1:100 dilution of anti-CD19 (BD Biosciences, BUV395), anti-CD27 (BioLegend, PE-Cy7), and anti-CD38 (BioLegend, APC) for 30 min at 4°C, and sorted using a BD FACSAria II Cell Sorter (BD Biosciences).

Cells were prelabeled with CellTrace Violet proliferation dye (Thermo Fisher Scientific) before being loaded into the device. The dye was diluted in medium for flow cytometry experiments and incubated for 5 min. For imaging experiments, the dye was diluted in phosphate-buffered saline (PBS) and incubated for 30 min.

Between 40,000 and 60,000 organoid-derived B cells were loaded into the device 4 days after gel formation via the media channels. Specifically, the cells were resuspended in EGM-2 MV supplemented with VEGF and ANG-1 and loaded into one side of each media channel well. Negative pressure was applied to the opposite side of the media channel to draw the cells into the device. The cytokines included in the media were determined experimentally and comprised BAFF (100 ng/ml) (BioLegend) and IL- 21 (10 ng/ml) (PeproTech). Every 4 days, fresh medium was added to the top of each media channel to nourish the devices.

### Immunohistochemistry

Devices were fixed in 4% paraformaldehyde (Thermo Fisher Scientific) for 10 min and permeabilized using 0.1% Triton X-100 for 15 min. Primary antibodies were diluted in 10% normal goat serum (NGS) (Thermo Fisher Scientific), and the devices were stained overnight at 4°C. Following this, they were washed with 0.1% bovine serum albumin (BSA) in PBS and then stained with secondary antibodies diluted in 10% NGS for 2 hours at room temperature. After washing with a wash buffer, the devices were imaged using a spinning disk confocal microscope (PerkinElmer) at ×10 or ×20 magnification. Primary antibodies included the following: 1:100 anti-CXCL12 (Abcam, rabbit), 1:100 anti-osteocalcin (R&D Systems, AF647, Bioss, AF555), 1:100 anti-osteopontin (Bioss, AF647), 1:100 anti-fibronectin (Abcam, AF555), 1:100 anti-CD31 (BioLegend, AF594 & AF488), 1:100 anti-CD90 (BioLegend, AF647), 1:100 anti-heparan sulfate proteoglycan 2 (Abcam, rabbit), 1:120 anti–VCAM-1 (Abcam, rabbit), 1:200 anti–collagen-IV (eBioscience, AF488), 1:30 biotinylated hyaluronic acid binding protein (Amsbio), and 0.5 U per device 488 phalloidin (Biotium). Secondary antibodies were as follows: 1:200 AF555 anti-rabbit (Invitrogen) and 1:200 AF647 streptavidin (Invitrogen).

### Flow cytometry analysis

For single-cell analysis of hBMOC cells, the cells needed to be extracted from the device. This process involved removing all media and washing the channels with PBS. The channels were then filled with 0.25% trypsin (Gibco) and incubated for 30 min at 37°C. A p200 pipette was used to mix the central channel, aiding in shearing and dissociating the hydrogel to mechanically detach the cells. Subsequently, the trypsin solution was collected, and the device was flushed with an equal volume of media to gather any remaining cells. The cell suspension was washed in PBS and centrifuged at 300*g* for 5 min to pellet the cells, which were subsequently used for analysis.

Initially, cells were stained with the viability dye Fixable Blue (Thermo Fisher Scientific) in PBS for 30 min at 4°C. Following this, cells in PBS containing 0.5% (w/v) BSA, 0.1% (w/v) sodium azide, and 2 mM EDTA were stained using various combinations of antibodies: anti-CD19 (BD Biosciences, BUV395), anti-CD138 (BD Biosciences, PE), anti-CD27 (BioLegend, PE/Cy7), anti-CD38 (BioLegend, APC), anti-CD20 (BioLegend, BV605), anti-CD45 (Invitrogen, PerCP/Cy5.5), and anti-HLA-DR (Invitrogen, SB780) for 30 min at 4°C. The staining buffer did not include an Fc block. In general, 0.06 to 1 μg of antibody was applied to stain about 10^5^ to 10^7^ cells in 100 μl. For intracellular staining, samples were fixed and permeabilized in the Foxp3/Transcription Factor Staining Buffer (eBioscience) following the manufacturer’s guidelines and then stained with anti-Ki67 (eBioscience, fluorescein isothiocyanate) and anti–BCL-2 (Bioss, AF680) for 30 min at room temperature at a dilution of 1:100. All samples were analyzed using a Cytek Aurora (Cytek Biosciences), and the data were evaluated with SpectroFlo (Cytek Biosciences) and the FlowJo software (Becton Dickinson).

### Multiplexed cytokine detection

After 4 days, samples collected from devices were frozen until analysis. Before detection and analysis, samples were thawed and then assessed using a LEGENDplex human B cell panel (BioLegend), following the manufacturer’s protocol. Readings were conducted on a CytoFLEX S flow cytometer (Beckman Coulter).

### Motility experiments, live imaging, and analysis

After loading membrane-labeled B cells into the hBMOC, the devices were allowed to rest for at least 4 hours, enabling the cells to enter the central channel. Following this, the medium was supplemented with the following dilutions: AMD3100 (1000 ng/ml; Tocris), α-CXCL12 (30 μg/ml; Peprotech, rabbit, product #500-P87A-1MG), rabbit IgG isotype control (30 μg/ml; R&D Systems), or CXCL12α (100 ng/ml; Peprotech). The CXCL12α was added to only one media channel to establish a gradient. The devices were then imaged using an incubated Lionheart FX epifluorescence microscope (BioTek Instruments), with time-lapse recordings taken every 9 s for 20 min or every 3 min for 10 hours. Image analysis was performed using ImageJ, where images of cells in the central channel were combined into stacks and adjusted for movement caused by stage drift with an ImageJ plugin (Template_Matching.jar). Individual cell motility was assessed using the TrackMate plugin in ImageJ to quantify metrics such as track displacement, speed, total distance traveled, mean directional change rate, and confinement ratio. The data were filtered to exclude regions of interests (ROIs) containing filaments and cell tracks shorter than 3 hours and then analyzed with custom MATLAB and R scripts.

### Statistical analysis and reproducibility

All studies were conducted three times, yielding consistent results across independent experimental runs. Investigators remained blind during imaging analyses, flow cytometry experiments, and formulation tests. For imaging and inhibitor studies, they were unaware of group allocation during data collection and/or analysis. Blinding was implemented without disclosing the organoid types or treatment groups. However, for one experiment, investigators were not blinded regarding group allocation during data collection and/or analysis due to limited resources for effective blinding and the necessity of knowing group allocation in some cases to uphold data integrity and quality control. Sample sizes were determined on the basis of statistical power calculations and prior experience with these metrics. Data from intact hydrogels were not excluded. All statistical analyses were conducted using GraphPad Prism v.9.5.1 software. Data are typically presented as mean ± SEM unless stated otherwise, with specific statistical tests outlined in figure legends. All data underwent formal tests for normality or statistical assumptions, and necessary statistical corrections were made. Specific statistical tests are indicated in the figure captions.

## Supplementary Materials

### The PDF file includes:

Figs. S1 to S4 Legend for data file S1 All data R code

## References

[1] X. Liu, J. Yao, Y. Zhao, J. Wang, H. Qi, Heterogeneous plasma cells and long-lived subsets in response to immunization, autoantigen and microbiota. Nat. Immunol. 23, 1564–1576 (2022). 36316480 10.1038/s41590-022-01345-5

[2] D. C. Nguyen, C. J. Joyner, I. Sanz, F. E. Lee, Factors affecting early antibody secreting cell maturation into long-lived plasma cells. Front. Immunol. 10, 2138 (2019). 31572364 10.3389/fimmu.2019.02138 PMC6749102

[3] A. Singh, Eliciting B cell immunity against infectious diseases using nanovaccines. Nat. Nanotechnol. 16, 16–24 (2021). 33199883 10.1038/s41565-020-00790-3 PMC7855692

[4] S. F. Brynjolfsson, L. Persson Berg, T. Olsen Ekerhult, I. Rimkute, M. J. Wick, I. L. Mårtensson, O. Grimsholm, Long-lived plasma cells in mice and men. Front. Immunol. 9, 2673 (2018). 30505309 10.3389/fimmu.2018.02673 PMC6250827

[5] A. Radbruch, G. Muehlinghaus, E. O. Luger, A. Inamine, K. G. Smith, T. Dörner, F. Hiepe, Competence and competition: The challenge of becoming a long-lived plasma cell. Nat. Rev. Immunol. 6, 741–750 (2006). 16977339 10.1038/nri1886

[6] B. D. Simons, O. Karin, Tuning of plasma cell lifespan by competition explains the longevity and heterogeneity of antibody persistence. Immunity 57, 600–611.e6 (2024). 38447570 10.1016/j.immuni.2024.02.005

[7] D. Tarlinton, Do plasma cells contribute to the determination of their lifespan? Immunol. Cell Biol. 98, 449–455 (2020). 32353190 10.1111/imcb.12346

[8] I. J. Amanna, M. K. Slifka, Mechanisms that determine plasma cell lifespan and the duration of humoral immunity. Immunol. Rev. 236, 125–138 (2010). 20636813 10.1111/j.1600-065X.2010.00912.x PMC7165522

[9] L. Khodadadi, Q. Cheng, A. Radbruch, F. Hiepe, The maintenance of memory plasma cells. Front. Immunol. 10, 721 (2019). 31024553 10.3389/fimmu.2019.00721 PMC6464033

[10] S. M. Lightman, A. Utley, K. P. Lee, Survival of long-lived plasma cells (LLPC): Piecing together the puzzle. Front. Immunol. 10, 965 (2019). 31130955 10.3389/fimmu.2019.00965 PMC6510054

[11] K. Kometani, T. Kurosaki, Differentiation and maintenance of long-lived plasma cells. Curr. Opin. Immunol. 33, 64–69 (2015). 25677584 10.1016/j.coi.2015.01.017

[12] S. Bandyopadhyay, M. P. Duffy, K. J. Ahn, J. H. Sussman, M. Pang, D. Smith, G. Duncan, I. Zhang, J. Huang, Y. Lin, B. Xiong, T. Imtiaz, C. H. Chen, A. Thadi, C. Chen, J. Xu, M. Reichart, Z. Martinez, C. Diorio, C. Chen, V. Pillai, O. Snaith, D. Oldridge, S. Bhattacharyya, I. Maillard, M. Carroll, C. Nelson, L. Qin, K. Tan, Mapping the cellular biogeography of human bone marrow niches using single-cell transcriptomics and proteomic imaging. Cell 187, 3120–3140.e29 (2024). 38714197 10.1016/j.cell.2024.04.013 PMC11162340

[13] D. Toscani, M. Bolzoni, F. Accardi, F. Aversa, N. Giuliani, The osteoblastic niche in the context of multiple myeloma. Ann. N. Y. Acad. Sci. 1335, 45–62 (2015). 25424768 10.1111/nyas.12578

[14] D. C. Nguyen, S. Garimalla, H. Xiao, S. Kyu, I. Albizua, J. Galipeau, K.-Y. Chiang, E. K. Waller, R. Wu, G. Gibson, J. Roberson, F. E. Lund, T. D. Randall, I. Sanz, F. E.-H. Lee, Factors of the bone marrow microniche that support human plasma cell survival and immunoglobulin secretion. Nat. Commun. 9, 3698 (2018). 30209264 10.1038/s41467-018-05853-7 PMC6135805

[15] E. Belnoue, C. Tougne, A. F. Rochat, P. H. Lambert, D. D. Pinschewer, C. A. Siegrist, Homing and adhesion patterns determine the cellular composition of the bone marrow plasma cell niche. J. Immunol. 188, 1283–1291 (2012). 22262758 10.4049/jimmunol.1103169

[16] M. Rodriguez Gomez, Y. Talke, N. Goebel, F. Hermann, B. Reich, M. Mack, Basophils support the survival of plasma cells in mice. J. Immunol. 185, 7180–7185 (2010). 21068399 10.4049/jimmunol.1002319

[17] V. T. Chu, A. Fröhlich, G. Steinhauser, T. Scheel, T. Roch, S. Fillatreau, J. J. Lee, M. Löhning, C. Berek, Eosinophils are required for the maintenance of plasma cells in the bone marrow. Nat. Immunol. 12, 151–159 (2011). 21217761 10.1038/ni.1981

[18] O. Winter, K. Moser, E. Mohr, D. Zotos, H. Kaminski, M. Szyska, K. Roth, D. M. Wong, C. Dame, D. M. Tarlinton, H. Schulze, I. C. M. Mac Lennan, R. A. Manz, Megakaryocytes constitute a functional component of a plasma cell niche in the bone marrow. Blood 116, 1867–1875 (2010). 20538807 10.1182/blood-2009-12-259457

[19] V. T. Chu, C. Berek, The establishment of the plasma cell survival niche in the bone marrow. Immunol. Rev. 251, 177–188 (2013). 23278749 10.1111/imr.12011

[20] M. J. Benson, S. R. Dillon, E. Castigli, R. S. Geha, S. Xu, K.-P. Lam, R. J. Noelle, Cutting edge: The dependence of plasma cells and independence of memory B cells on BAFF and APRIL. J. Immunol. 180, 3655–3659 (2008). 18322170 10.4049/jimmunol.180.6.3655

[21] H. A. Minges Wols, G. H. Underhill, G. S. Kansas, P. L. Witte, The role of bone marrow-derived stromal cells in the maintenance of plasma cell longevity. J. Immunol. 169, 4213–4221 (2002). 12370351 10.4049/jimmunol.169.8.4213

[22] D. T. Avery, S. L. Kalled, J. I. Ellyard, C. Ambrose, S. A. Bixler, M. Thien, R. Brink, F. Mackay, P. D. Hodgkin, S. G. Tangye, BAFF selectively enhances the survival of plasmablasts generated from human memory B cells. J. Clin. Invest. 112, 286–297 (2003). 12865416 10.1172/JCI18025 PMC164292

[23] D. B. Chou, V. Frismantas, Y. Milton, R. David, P. Pop-Damkov, D. Ferguson, A. M. Donald, Ö. V. Bölükbaşı, C. E. Joyce, L. S. M. Teixeira, A. Rech, A. Jiang, E. Calamari, S. Jalili-Firoozinezhad, B. A. Furlong, L. R. O’Sullivan, C. F. Ng, Y. Choe, S. Marquez, K. C. Myers, O. K. Weinberg, R. P. Hasserjian, R. Novak, O. Levy, R. Prantil-Baun, C. D. Novina, A. Shimamura, L. Ewart, D. E. Ingber, On-chip recapitulation of clinical bone marrow toxicities and patient-specific pathophysiology. *Nat*. Biomed. Eng. 4, 394–406 (2020). 10.1038/s41551-019-0495-z PMC7160021 31988457

[24] D. E. Glaser, M. B. Curtis, P. A. Sariano, Z. A. Rollins, B. S. Shergill, A. Anand, A. M. Deely, V. S. Shirure, L. Anderson, J. M. Lowen, N. R. Ng, K. Weilbaecher, D. C. Link, S. C. George, Organ-on-a-chip model of vascularized human bone marrow niches. Biomaterials 280, 121245 (2022). 34810038 10.1016/j.biomaterials.2021.121245 PMC10658812

[25] A. Sharipol, M. L. Lesch, C. A. Soto, B. J. Frisch, Bone marrow microenvironment-on-chip for culture of functional hematopoietic stem cells. Front. Bioeng. Biotechnol. 10, 855777 (2022). 35795163 10.3389/fbioe.2022.855777 PMC9252162

[26] G. B. Thompson, V. R. Barnhouse, S. K. Bierman, B. A. C. Harley, Influence of hypoxia on a biomaterial model of the bone marrow perivascular niche. Adv. Healthc. Mater. 14, e2500858 (2025). 40285591 10.1002/adhm.202500858 PMC12118339

[27] I. Baldassarri, D. N. Tavakol, P. L. Graney, A. G. Chramiec, H. Hibshoosh, G. Vunjak-Novakovic, An engineered model of metastatic colonization of human bone marrow reveals breast cancer cell remodeling of the hematopoietic niche. Proc. Natl. Acad. Sci. U.S.A. 121, e2405257121 (2024). 39374382 10.1073/pnas.2405257121 PMC11494322

[28] M. R. Nelson, D. Ghoshal, J. C. Mejías, D. F. Rubio, E. Keith, K. Roy, A multi-niche microvascularized human bone marrow (hBM) on-a-chip elucidates key roles of the endosteal niche in hBM physiology. Biomaterials 270, 120683 (2021). 33556648 10.1016/j.biomaterials.2021.120683

[29] D. Ghoshal, I. Petersen, R. Ringquist, L. Kramer, E. Bhatia, T. Hu, A. Richard, R. Park, J. Corbin, S. Agarwal, A. Thomas, S. Ramirez, J. Tharayil, E. Downey, F. Ketchum, A. Ochal, N. Sonthi, S. Lonial, J. N. Kochenderfer, R. Tran, M. Zhu, W. A. Lam, A. F. Coskun, K. Roy, Multi-niche human bone marrow on-a-chip for studying the interactions of adoptive CAR-T cell therapies with multiple myeloma. Biomaterials 316, 123016 (2025). 39709851 10.1016/j.biomaterials.2024.123016

[30] Z. Zhong, M. Quinones-Perez, Z. Dai, V. M. Juarez, E. Bhatia, C. R. Carlson, S. B. Shah, A. Patel, Z. Fang, T. Hu, M. Allam, S. L. Hicks, M. Gupta, S. L. Gupta, E. Weeks, S. D. Vagelos, A. Molina, A. Mulero-Russe, A. Mora-Boza, D. J. Joshi, R. P. Sekaly, T. Sulchek, S. L. Goudy, J. Wrammert, K. Roy, J. M. Boss, A. F. Coskun, C. D. Scharer, A. J. Garcia, J. L. Koff, A. Singh, Human immune organoids to decode B cell response in healthy donors and patients with lymphoma. Nat. Mater. 24, 297–311 (2025). 39506098 10.1038/s41563-024-02037-1 PMC11866935

[31] D. L. Coutu, K. D. Kokkaliaris, L. Kunz, T. Schroeder, Three-dimensional map of nonhematopoietic bone and bone-marrow cells and molecules. Nat. Biotechnol. 35, 1202–1210 (2017). 29131149 10.1038/nbt.4006

[32] A. Salvadè, D. Belotti, E. Donzelli, G. D’Amico, G. Gaipa, G. Renoldi, F. Carini, M. Baldoni, E. M. Pogliani, G. Tredici, A. Biondi, E. Biagi, GMP-grade preparation of biomimetic scaffolds with osteo-differentiated autologous mesenchymal stromal cells for the treatment of alveolar bone resorption in periodontal disease. Cytotherapy 9, 427–438 (2007). 17786604 10.1080/14653240701341995

[33] R. Yao, J. Y. Wong, The effects of mechanical stimulation on controlling and maintaining marrow stromal cell differentiation into vascular smooth muscle cells. J. Biomech. Eng. 137, 020907 (2015). 25429403 10.1115/1.4029255 PMC4321111

[34] X. Huang, B. Zhu, X. Wang, R. Xiao, C. Wang, Three-dimensional co-culture of mesenchymal stromal cells and differentiated osteoblasts on human bio-derived bone scaffolds supports active multi-lineage hematopoiesis in vitro: Functional implication of the biomimetic HSC niche. Int. J. Mol. Med. 38, 1141–1151 (2016). 27571775 10.3892/ijmm.2016.2712 PMC5029969

[35] J. A. Whisler, M. B. Chen, R. D. Kamm, Control of perfusable microvascular network morphology using a multiculture microfluidic system. Tissue Eng. Part C Methods 20, 543–552 (2014). 24151838 10.1089/ten.tec.2013.0370 PMC4074745

[36] R. K. H. Yip, J. Er, L. Qin, Q. H. Nguyen, A. Motyer, J. S. Rimes, A. Light, R. D. Mishi, L. Ling, C. J. A. Anttila, E. Tsui, D. Amann-Zalcenstein, M. R. Dowling, K. L. Rogers, R. Bowden, Y. Chen, S. J. Harrison, E. D. Hawkins, Profiling the spatial architecture of multiple myeloma in human bone marrow trephine biopsy specimens with spatial transcriptomics. Blood 146, 1837–1849 (2025). 40643106 10.1182/blood.2025028896 PMC12824660

[37] K. Aoki, M. Kurashige, M. Ichii, K. Higaki, T. Sugiyama, T. Kaito, W. Ando, N. Sugano, T. Sakai, H. Shibayama, HANDAI Clinical Blood Club, A. Takaori-Kondo, E. Morii, Y. Kanakura, T. Nagasawa, Identification of CXCL12-abundant reticular cells in human adult bone marrow. Br. J. Haematol. 193, 659–668 (2021). 33837967 10.1111/bjh.17396 PMC8252541

[38] A. Sarachakov, A. Varlamova, V. Svekolkin, M. Polyakova, I. Valencia, C. Unkenholz, T. Pannellini, I. Galkin, P. Ovcharov, D. Tabakov, E. Postovalova, N. Shin, I. Sethi, A. Bagaev, T. Itkin, G. Crane, M. Kluk, J. Geyer, G. Inghirami, S. Patel, Spatial mapping of human hematopoiesis at single-cell resolution reveals aging-associated topographic remodeling. Blood 142, 2282–2295 (2023). 37774374 10.1182/blood.2023021280

[39] S. Abe-Suzuki, M. Kurata, S. Abe, I. Onishi, S. Kirimura, M. Nashimoto, T. Murayama, M. Hidaka, M. Kitagawa, CXCL12^+^ stromal cells as bone marrow niche for CD34^+^ hematopoietic cells and their association with disease progression in myelodysplastic syndromes. Lab. Invest. 94, 1212–1223 (2014). 25199050 10.1038/labinvest.2014.110

[40] M. Duan, D. C. Nguyen, C. J. Joyner, C. L. Saney, C. M. Tipton, J. Andrews, S. Lonial, C. Kim, I. Hentenaar, A. Kosters, E. Ghosn, A. Jackson, S. Knechtle, S. Maruthamuthu, S. Chandran, T. Martin, R. Rajalingam, F. Vincenti, C. Breeden, I. Sanz, G. Gibson, F. E.-H. Lee, Understanding heterogeneity of human bone marrow plasma cell maturation and survival pathways by single-cell analyses. Cell Rep. 42, 112682 (2023). 37355988 10.1016/j.celrep.2023.112682 PMC10391632

[41] B. Meza-León, D. Gratzinger, A. G. Aguilar-Navarro, F. G. Juárez-Aguilar, V. I. Rebel, E. Torlakovic, L. E. Purton, E. M. Dorantes-Acosta, A. Escobar-Sánchez, J. E. Dick, E. Flores-Figueroa, Human, mouse, and dog bone marrow show similar mesenchymal stromal cells within a distinctive microenvironment. Exp. Hematol. 100, 41–51 (2021). 34228982 10.1016/j.exphem.2021.06.006

[42] S. K. Nilsson, M. E. Debatis, M. S. Dooner, J. A. Madri, P. J. Quesenberry, P. S. Becker, Immunofluorescence characterization of key extracellular matrix proteins in murine bone marrow in situ. J. Histochem. Cytochem. 46, 371–377 (1998). 9487119 10.1177/002215549804600311

[43] C. Baccin, J. Al-Sabah, L. Velten, P. M. Helbling, F. Grünschläger, P. Hernández-Malmierca, C. Nombela-Arrieta, L. M. Steinmetz, A. Trumpp, S. Haas, Combined single-cell and spatial transcriptomics reveal the molecular, cellular and spatial bone marrow niche organization. Nat. Cell Biol. 22, 38–48 (2020). 31871321 10.1038/s41556-019-0439-6 PMC7610809

[44] C. Xu, X. Gao, Q. Wei, F. Nakahara, S. E. Zimmerman, J. Mar, P. S. Frenette, Stem cell factor is selectively secreted by arterial endothelial cells in bone marrow. Nat. Commun. 9, 2449 (2018). 29934585 10.1038/s41467-018-04726-3 PMC6015052

[45] A. Greenbaum, Y. M. Hsu, R. B. Day, L. G. Schuettpelz, M. J. Christopher, J. N. Borgerding, T. Nagasawa, D. C. Link, CXCL12 in early mesenchymal progenitors is required for haematopoietic stem-cell maintenance. Nature 495, 227–230 (2013). 23434756 10.1038/nature11926 PMC3600148

[46] C. Laguri, F. Arenzana-Seisdedos, H. Lortat-Jacob, Relationships between glycosaminoglycan and receptor binding sites in chemokines—The CXCL12 example. Carbohydr. Res. 343, 2018–2023 (2008). 18334249 10.1016/j.carres.2008.01.047

[47] N. Panitz, S. Theisgen, S. A. Samsonov, J.-P. Gehrcke, L. Baumann, K. Bellmann-Sickert, S. Köhling, M. T. Pisabarro, J. Rademann, D. Huster, A. G. Beck-Sickinger, The structural investigation of glycosaminoglycan binding to CXCL12 displays distinct interaction sites. Glycobiology 26, 1209–1221 (2016). 27496764 10.1093/glycob/cww059

[48] D. Thakar, F. Dalonneau, E. Migliorini, H. Lortat-Jacob, D. Boturyn, C. Albiges-Rizo, L. Coche-Guerente, C. Picart, R. P. Richter, Binding of the chemokine CXCL12α to its natural extracellular matrix ligand heparan sulfate enables myoblast adhesion and facilitates cell motility. Biomaterials 123, 24–38 (2017). 28152381 10.1016/j.biomaterials.2017.01.022 PMC5405871

[49] R. Tanikawa, T. Tanikawa, M. Hirashima, A. Yamauchi, Y. Tanaka, Galectin-9 induces osteoblast differentiation through the CD44/Smad signaling pathway. Biochem. Biophys. Res. Commun. 394, 317–322 (2010). 20206131 10.1016/j.bbrc.2010.02.175

[50] A. Bruce, R. Evans, R. Mezan, L. Shi, B. S. Moses, K. H. Martin, L. F. Gibson, Y. Yang, Three-dimensional microfluidic tri-culture model of the bone marrow microenvironment for study of acute lymphoblastic leukemia. PLOS ONE 10, e0140506 (2015). 26488876 10.1371/journal.pone.0140506 PMC4619215

[51] J. Jakubikova, D. Cholujova, T. Hideshima, P. Gronesova, A. Soltysova, T. Harada, J. Joo, S. Y. Kong, R. E. Szalat, P. G. Richardson, N. C. Munshi, D. M. Dorfman, K. C. Anderson, A novel 3D mesenchymal stem cell model of the multiple myeloma bone marrow niche: Biologic and clinical applications. Oncotarget 7, 77326–77341 (2016). 27764795 10.18632/oncotarget.12643 PMC5357212

[52] T. D. Moeller, S. B. Shah, K. Lai, N. Lopez-Barbosa, P. Desai, W. Wang, Z. Zhong, D. Redmond, A. Singh, M. P. DeLisa, Profiling germinal center-like B cell responses to conjugate vaccines using synthetic immune organoids. ACS Cent Sci 9, 787–804 (2023). 37122450 10.1021/acscentsci.2c01473 PMC10141597

[53] S. B. Shah, C. R. Carlson, K. Lai, Z. Zhong, G. Marsico, K. M. Lee, N. E. Felix Velez, E. B. Abeles, M. Allam, T. Hu, L. D. Walter, K. E. Martin, K. Gandhi, S. D. Butler, R. Puri, A. L. McCleary-Wheeler, W. Tam, O. Elemento, K. Takata, C. Steidl, D. W. Scott, L. Fontan, H. Ueno, B. D. Cosgrove, G. Inghirami, A. J. Garcia, A. F. Coskun, J. L. Koff, A. Melnick, A. Singh, Combinatorial treatment rescues tumour-microenvironment-mediated attenuation of MALT1 inhibitors in B-cell lymphomas. Nat. Mater. 22, 511–523 (2023). 36928381 10.1038/s41563-023-01495-3 PMC10069918

[54] S. Kim, S. B. Shah, P. L. Graney, A. Singh, Multiscale engineering of immune cells and lymphoid organs. Nat. Rev. Mater. 4, 355–378 (2019). 31903226 10.1038/s41578-019-0100-9 PMC6941786

[55] Y. S. Michaels, C. F. Buchanan, N. Gjorevski, A. Moisan, Bioengineering translational models of lymphoid tissues. Nat. Rev. Bioeng. 1, 731–748 (2023).

[56] E. A. Phelps, N. O. Enemchukwu, V. F. Fiore, J. C. Sy, N. Murthy, T. A. Sulchek, T. H. Barker, A. J. García, Maleimide cross-linked bioactive PEG hydrogel exhibits improved reaction kinetics and cross-linking for cell encapsulation and in situ delivery. Adv. Mater. 24, 64–70 (2012). 22174081 10.1002/adma.201103574 PMC3517145

[57] L. S. Britto, D. Balasubramani, S. Desai, P. Phillips, N. Trehan, E. Cesarman, J. L. Koff, A. Singh, T cells spatially regulate B cell receptor signaling in lymphomas through H3K9me3 modifications. Adv. Healthc. Mater. 14, e2401192 (2025). 38837879 10.1002/adhm.202401192 PMC11617604

[58] P. L. Graney, K. Lai, S. Post, I. Brito, J. Cyster, A. Singh, Organoid polymer functionality and mode of klebsiella pneumoniae membrane antigen presentation regulates ex vivo germinal center epigenetics in young and aged B cells. Adv. Funct. Mater. 30, 2001232 (2020). 33692664 10.1002/adfm.202001232 PMC7939142

[59] M. Goedhart, S. Gessel, R. van der Voort, E. Slot, B. Lucas, E. Gielen, M. Hoogenboezem, T. Rademakers, S. Geerman, J. D. van Buul, S. Huveneers, H. Dolstra, G. Anderson, C. Voermans, M. A. Nolte, CXCR4, but not CXCR3, drives CD8^+^ T-cell entry into and migration through the murine bone marrow. Eur. J. Immunol. 49, 576–589 (2019). 30707456 10.1002/eji.201747438

[60] D. C. Hargreaves, P. L. Hyman, T. T. Lu, V. N. Ngo, A. Bidgol, G. Suzuki, Y. R. Zou, D. R. Littman, J. G. Cyster, A coordinated change in chemokine responsiveness guides plasma cell movements. J. Exp. Med. 194, 45–56 (2001). 11435471 10.1084/jem.194.1.45 PMC2193440

[61] A. Peled, O. Kollet, T. Ponomaryov, I. Petit, S. Franitza, V. Grabovsky, M. M. Slav, A. Nagler, O. Lider, R. Alon, D. Zipori, T. Lapidot, The chemokine SDF-1 activates the integrins LFA-1, VLA-4, and VLA-5 on immature human CD34^+^ cells: Role in transendothelial/stromal migration and engraftment of NOD/SCID mice. Blood 95, 3289–3296 (2000). 10828007

[62] E. Roldán, A. García-Pardo, J. A. Brieva, VLA-4-fibronectin interaction is required for the terminal differentiation of human bone marrow cells capable of spontaneous and high rate immunoglobulin secretion. J. Exp. Med. 175, 1739–1747 (1992). 1588291 10.1084/jem.175.6.1739 PMC2119256

[63] A. G. Zaretsky, C. Konradt, F. Dépis, J. B. Wing, R. Goenka, D. G. Atria, J. S. Silver, S. Cho, A. I. Wolf, W. J. Quinn, J. B. Engiles, D. C. Brown, D. Beiting, J. Erikson, D. Allman, M. P. Cancro, S. Sakaguchi, L.-F. Lu, C. O. Benoist, C. A. Hunter, T regulatory cells support plasma cell populations in the bone marrow. Cell Rep. 18, 1906–1916 (2017). 28228257 10.1016/j.celrep.2017.01.067 PMC5361408

[64] J. R. Wilmore, D. Allman, Here, there, and anywhere? Arguments for and against the physical plasma cell survival niche. J. Immunol. 199, 839–845 (2017). 28739594 10.4049/jimmunol.1700461 PMC5651088

[65] J. R. Wilmore, B. T. Gaudette, D. Gómez Atria, R. L. Rosenthal, S. K. Reiser, W. Meng, A. M. Rosenfeld, E. T. Luning Prak, D. Allman, IgA plasma cells are long-lived residents of gut and bone marrow that express isotype- and tissue-specific gene expression patterns. Front. Immunol. 12, 791095 (2021). 35003110 10.3389/fimmu.2021.791095 PMC8739487

[66] Z. Benet, Z. Jing, D. R. Fooksman, Plasma cell dynamics in the bone marrow niche. Cell Rep. 34, 108733 (2021). 33567286 10.1016/j.celrep.2021.108733 PMC8023250

[67] N. Moore, M. Moreno Gonzales, K. Bonner, B. Smith, W. Park, M. Stegall, Impact of CXCR4/CXCL12 blockade on normal plasma cells in vivo. Am. J. Transplant. 17, 1663–1669 (2017). 28235241 10.1111/ajt.14236

[68] Q. Cheng, L. Khodadadi, A. Taddeo, J. Klotsche, B. F. Hoyer, A. Radbruch, F. Hiepe, CXCR4-CXCL12 interaction is important for plasma cell homing and survival in NZB/W mice. Eur. J. Immunol. 48, 1020–1029 (2018). 29427452 10.1002/eji.201747023

[69] K. Tokoyoda, T. Egawa, T. Sugiyama, B. I. Choi, T. Nagasawa, Cellular niches controlling B lymphocyte behavior within bone marrow during development. Immunity 20, 707–718 (2004). 15189736 10.1016/j.immuni.2004.05.001

[70] G. Cassese, S. Arce, A. E. Hauser, K. Lehnert, B. Moewes, M. Mostarac, G. Muehlinghaus, M. Szyska, A. Radbruch, R. A. Manz, Plasma cell survival is mediated by synergistic effects of cytokines and adhesion-dependent signals. J. Immunol. 171, 1684–1690 (2003). 12902466 10.4049/jimmunol.171.4.1684

[71] T. C. Beck, A. C. Gomes, J. G. Cyster, J. P. Pereira, CXCR4 and a cell-extrinsic mechanism control immature B lymphocyte egress from bone marrow. J. Exp. Med. 211, 2567–2581 (2014). 25403444 10.1084/jem.20140457 PMC4267240

[72] K. Roth, L. Oehme, S. Zehentmeier, Y. Zhang, R. Niesner, A. E. Hauser, Tracking plasma cell differentiation and survival. Cytometry A 85, 15–24 (2014). 24700574 10.1002/cyto.a.22355

[73] C. R. Smulski, H. Eibel, BAFF and BAFF-receptor in B cell selection and survival. Front. Immunol. 9, 2285 (2018). 30349534 10.3389/fimmu.2018.02285 PMC6186824

[74] L. Moens, S. G. Tangye, Cytokine-mediated regulation of plasma cell generation: IL-21 takes center stage. Front. Immunol. 5, 65 (2014). 24600453 10.3389/fimmu.2014.00065 PMC3927127

[75] G. Jego, N. Robillard, D. Puthier, M. Amiot, F. Accard, D. Pineau, J. L. Harousseau, R. Bataille, C. Pellat-Deceunynck, Reactive plasmacytoses are expansions of plasmablasts retaining the capacity to differentiate into plasma cells. Blood 94, 701–712 (1999). 10397737

[76] M. J. McCarron, P. W. Park, D. R. Fooksman, CD138 mediates selection of mature plasma cells by regulating their survival. Blood 129, 2749–2759 (2017). 28381397 10.1182/blood-2017-01-761643 PMC5437827

[77] R. C. Ridley, H. Xiao, H. Hata, J. Woodliff, J. Epstein, R. D. Sanderson, Expression of syndecan regulates human myeloma plasma cell adhesion to type I collagen. Blood 81, 767–774 (1993). 8427968

[78] D. R. Coombe, Biological implications of glycosaminoglycan interactions with haemopoietic cytokines. Immunol. Cell Biol. 86, 598–607 (2008). 18626488 10.1038/icb.2008.49

[79] S. Garimalla, D. C. Nguyen, J. L. Halliley, C. Tipton, A. F. Rosenberg, C. F. Fucile, C. L. Saney, S. Kyu, D. Kaminski, Y. Qian, R. H. Scheuermann, G. Gibson, I. Sanz, F. E.-H. Lee, Differential transcriptome and development of human peripheral plasma cell subsets. JCI Insight 4, e126732 (2019). 31045577 10.1172/jci.insight.126732 PMC6538338

[80] I. Sanz, C. Wei, S. A. Jenks, K. S. Cashman, C. Tipton, M. C. Woodruff, J. Hom, F. E.-H. Lee, Challenges and opportunities for consistent classification of human B Cell and plasma cell populations. Front. Immunol. 10, 2458 (2019). 31681331 10.3389/fimmu.2019.02458 PMC6813733

[81] H. E. Mei, T. Yoshida, W. Sime, F. Hiepe, K. Thiele, R. A. Manz, A. Radbruch, T. Dörner, Blood-borne human plasma cells in steady state are derived from mucosal immune responses. Blood 113, 2461–2469 (2009). 18987362 10.1182/blood-2008-04-153544

[82] J. L. Halliley, C. M. Tipton, J. Liesveld, A. F. Rosenberg, J. Darce, I. V. Gregoretti, L. Popova, D. Kaminiski, C. F. Fucile, I. Albizua, S. Kyu, K.-Y. Chiang, K. T. Bradley, R. Burack, M. Slifka, E. Hammarlund, H. Wu, L. Zhao, E. E. Walsh, A. R. Falsey, T. D. Randall, W. C. Cheung, I. Sanz, F. E.-H. Lee, Long-lived plasma cells are contained within the CD19^−^CD38^hi^CD138^+^ subset in human bone marrow. Immunity 43, 132–145 (2015). 26187412 10.1016/j.immuni.2015.06.016 PMC4680845

[83] T. S. Aaron, D. R. Fooksman, Dynamic organization of the bone marrow plasma cell niche. FEBS J. 289, 4228–4239 (2022). 35114061 10.1111/febs.16385

[84] F. Wang, T. Yasuhara, T. Shingo, M. Kameda, N. Tajiri, W. J. Yuan, A. Kondo, T. Kadota, T. Baba, J. T. Tayra, Y. Kikuchi, Y. Miyoshi, I. Date, Intravenous administration of mesenchymal stem cells exerts therapeutic effects on parkinsonian model of rats: Focusing on neuroprotective effects of stromal cell-derived factor-1α. BMC Neurosci. 11, 52 (2010). 20420688 10.1186/1471-2202-11-52 PMC2873592

[85] G. Su, S. A. Blaine, D. Qiao, A. Friedl, Shedding of syndecan-1 by stromal fibroblasts stimulates human breast cancer cell proliferation via FGF2 activation. J. Biol. Chem. 282, 14906–14915 (2007). 17344212 10.1074/jbc.M611739200

[86] Q. Liu, Z. Li, J. L. Gao, W. Wan, S. Ganesan, D. H. McDermott, P. M. Murphy, CXCR4 antagonist AMD3100 redistributes leukocytes from primary immune organs to secondary immune organs, lung, and blood in mice. Eur. J. Immunol. 45, 1855–1867 (2015). 25801950 10.1002/eji.201445245 PMC4461468

[87] A. K. Azab, J. M. Runnels, C. Pitsillides, A. S. Moreau, F. Azab, X. Leleu, X. Jia, R. Wright, B. Ospina, A. L. Carlson, C. Alt, N. Burwick, A. M. Roccaro, H. T. Ngo, M. Farag, M. R. Melhem, A. Sacco, N. C. Munshi, T. Hideshima, B. J. Rollins, K. C. Anderson, A. L. Kung, C. P. Lin, I. M. Ghobrial, CXCR4 inhibitor AMD3100 disrupts the interaction of multiple myeloma cells with the bone marrow microenvironment and enhances their sensitivity to therapy. Blood 113, 4341–4351 (2009). 19139079 10.1182/blood-2008-10-186668 PMC2676090

[88] R. Park, Z. Benet, Z. Jing, J. Enright, D. R. Fooksman, CD138 and APRIL regulate plasma cell survival, competition, and retention in the bone marrow niche. Cell Rep. 44, 116123 (2025). 40768334 10.1016/j.celrep.2025.116123 PMC12461736

[89] E. Belnoue, M. Pihlgren, T. L. McGaha, C. Tougne, A.-F. Rochat, C. Bossen, P. Schneider, B. Huard, P.-H. Lambert, C.-A. Siegrist, APRIL is critical for plasmablast survival in the bone marrow and poorly expressed by early-life bone marrow stromal cells. Blood 111, 2755–2764 (2008). 18180376 10.1182/blood-2007-09-110858

[90] K. Ingold, A. Zumsteg, A. Tardivel, B. Huard, Q.-G. Steiner, T. G. Cachero, F. Qiang, L. Gorelik, S. L. Kalled, H. Acha-Orbea, P. D. Rennert, J. Tschopp, P. Schneider, Identification of proteoglycans as the APRIL-specific binding partners. J. Exp. Med. 201, 1375–1383 (2005). 15851487 10.1084/jem.20042309 PMC2213192

[91] D. C. Nguyen, M. Duan, M. Ali, A. Ley, I. Sanz, F. E. H. Lee, Plasma cell survival: The intrinsic drivers, migratory signals, and extrinsic regulators. Immunol. Rev. 303, 138–153 (2021). 34337772 10.1111/imr.13013 PMC8387437

[92] R. Ringquist, E. Bhatia, P. Chatterjee, D. Maniar, Z. Fang, P. Franz, L. Kramer, D. Ghoshal, N. Sonthi, E. Downey, J. Canlas, A. Ochal, S. Agarwal, V. Cuellar, G. Harrigan, A. F. Coskun, A. Singh, K. Roy, An immune-competent lung-on-a-chip for modelling the human severe influenza infection response. *Nat*. Biomed. Eng. 10, 897–919 (2026). 10.1038/s41551-025-01491-9 PMC12969995 40987954

[93] W. Wu, J. Wu, J. H. Kim, N. Y. Lee, Instantaneous room temperature bonding of a wide range of non-silicon substrates with poly(dimethylsiloxane) (PDMS) elastomer mediated by a mercaptosilane. Lab Chip 15, 2819–2825 (2015). 26014886 10.1039/c5lc00285k

[94] J. S. Jeon, S. Bersini, M. Gilardi, G. Dubini, J. L. Charest, M. Moretti, R. D. Kamm, Human 3D vascularized organotypic microfluidic assays to study breast cancer cell extravasation. Proc. Natl. Acad. Sci. U.S.A. 112, 214–219 (2015). 25524628 10.1073/pnas.1417115112 PMC4291627

[95] J. S. Jeon, S. Bersini, J. A. Whisler, M. B. Chen, G. Dubini, J. L. Charest, M. Moretti, R. D. Kamm, Generation of 3D functional microvascular networks with human mesenchymal stem cells in microfluidic systems. Integr. Biol. 6, 555–563 (2014). 10.1039/c3ib40267c PMC4307755 24676392

[96] Y. F. Tian, H. Ahn, R. S. Schneider, S. N. Yang, L. Roman-Gonzalez, A. M. Melnick, L. Cerchietti, A. Singh, Integrin-specific hydrogels as adaptable tumor organoids for malignant B and T cells. Biomaterials 73, 110–119 (2015). 26406451 10.1016/j.biomaterials.2015.09.007 PMC4623826

