# Supplementary material for: Ex vivo bone marrow subniches influence the fate of human antibody-secreting cells
Source: Sci Adv. 2026 Sep 11;12(37):eadz3976. doi: 10.1126/sciadv.adz3976 (PMC13564807; doi:10.1126/sciadv.adz3976)
Supplement: Supplementary file 1 [file sciadv.adz3976_sm.pdf]

Science Advances
AAAS

Supplementary Materials for

Ex vivo bone marrow subniches influence the fate of human antibody-secreting cells

Liana Kramer *et al.*

Corresponding author: Ankur Singh, ankur.singh@gatech.edu; Krishnendu Roy, krish.roy@vanderbilt.edu

Sci. Adv. **12**, eadz3976 (2026)
DOI: 10.1126/sciadv.adz3976

The PDF file includes:

Figs. S1 to S4
Legend for data file S1
All data R code

Other Supplementary Material for this manuscript includes the following:

Data file S1

Supplemental Figure S1: (A) Representative Z-plot profiles of signal from ASCs (blue) and CXCL12 (purple) or VCAM1 (purple), with dotted lines denoting peak slices for each signal. A black dotted line denotes a shared peak channel. (B) Quantification of the distance between z plane peaks for ASCs and CXCL12 and ASCs and VCAM1. Average is denoted by red line, with a $\Delta z<1$ indicating spatial overlap within the axial resolution of the microscope.

Supplemental Figure S2: (A) Representative gating strategy for B cells. CD45+ cells are then gated as ASCs and CD138+ PC as shown in Figures 3 and 4. (B) ASC and CD138+ PC counts after 4 days of culture in the BMOC, normalized to the amount introduced into the BMOC.

Supplemental Figure S3: (A) Representative images of CXCL12 staining in the BMOC devices with the Full Niche, No MSC, No OB, and HUVEC Only conditions.

Supplemental Figure S4: Representative images and clustering analysis plots from (A) devices treated with AMD3100 or (b) untreated devices, with “cluster 0” corresponding t6o all non-clustered cells.

Supplemental Figure S1

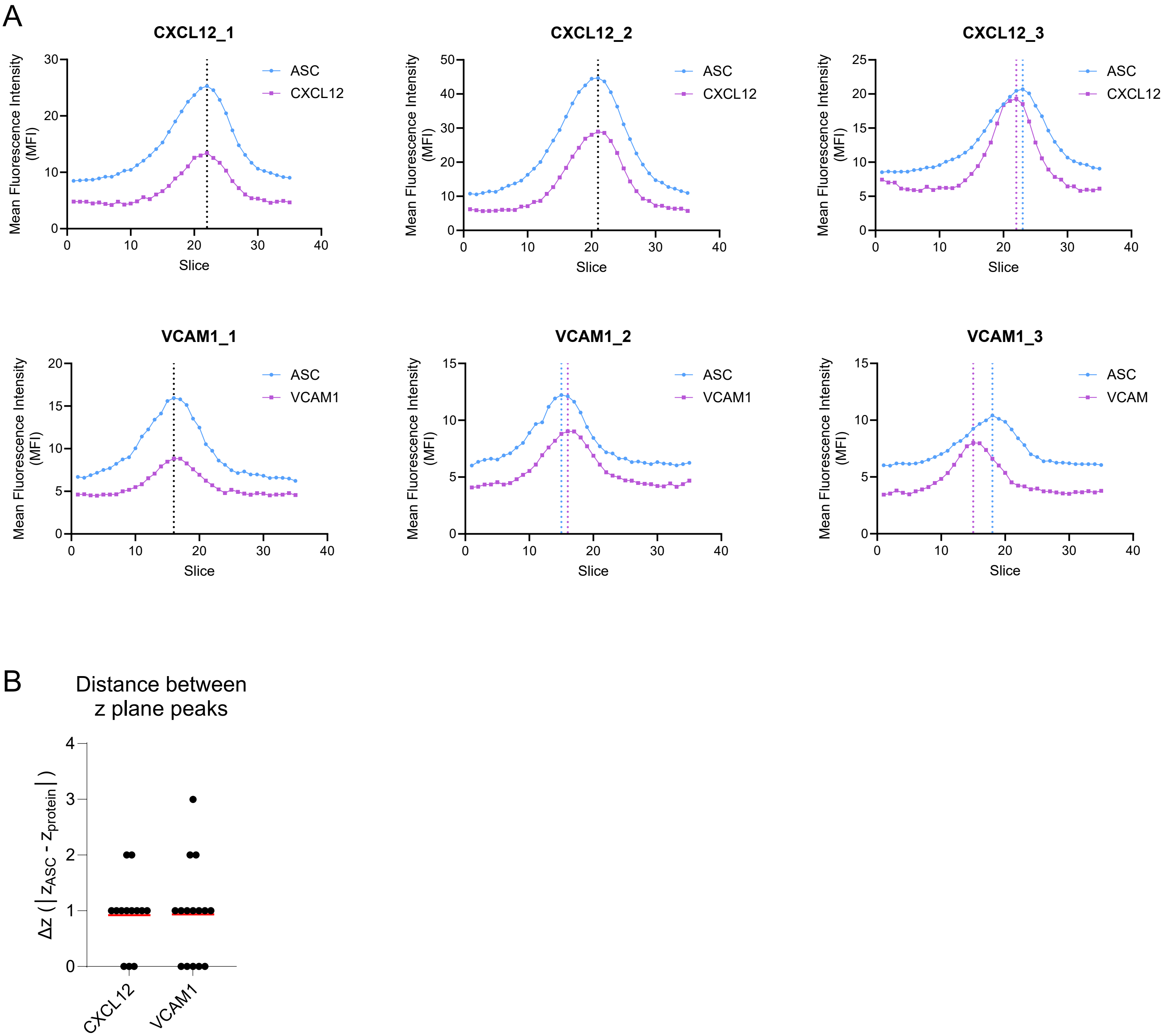

Supplemental Figure S2

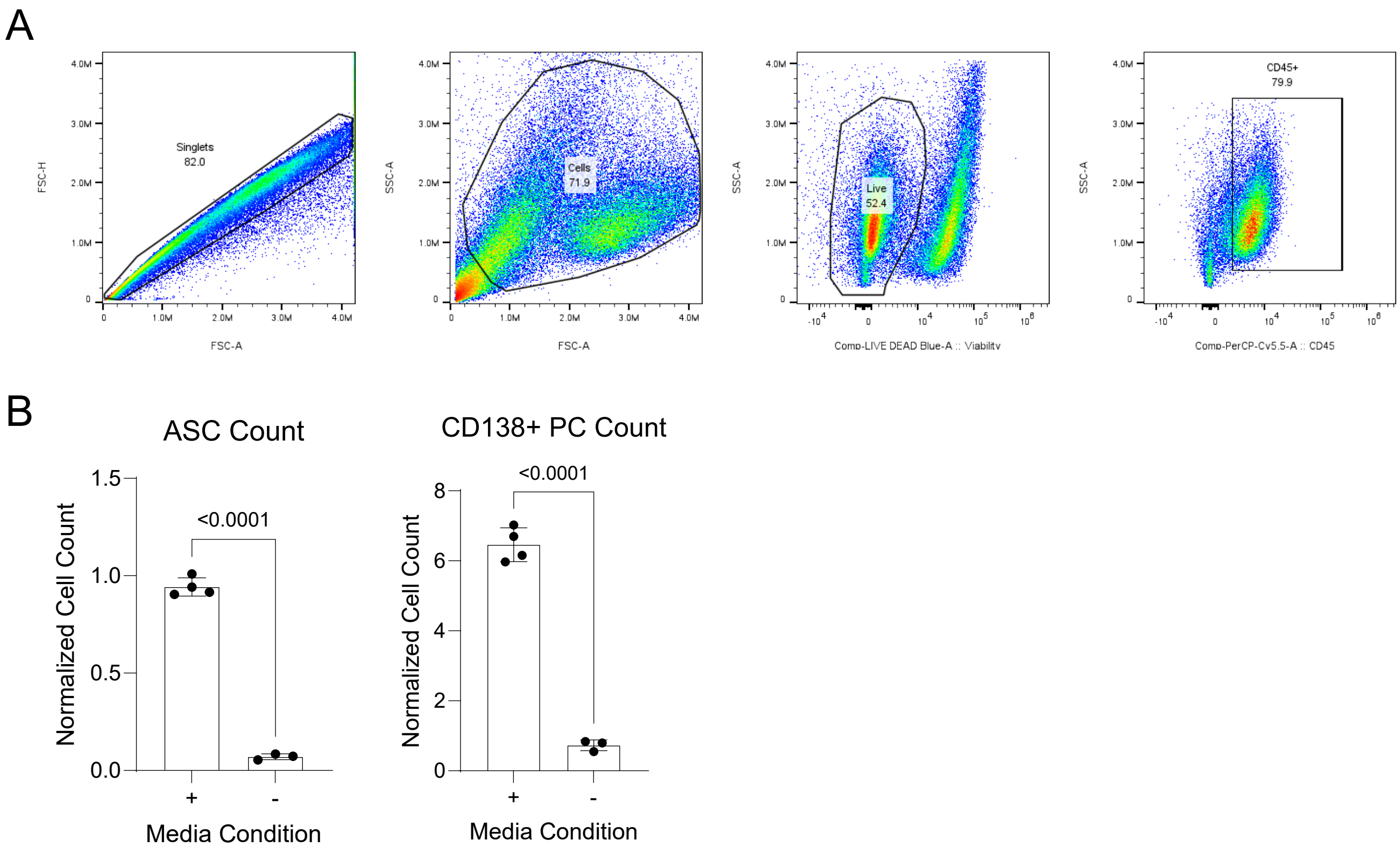

Supplemental Figure S3

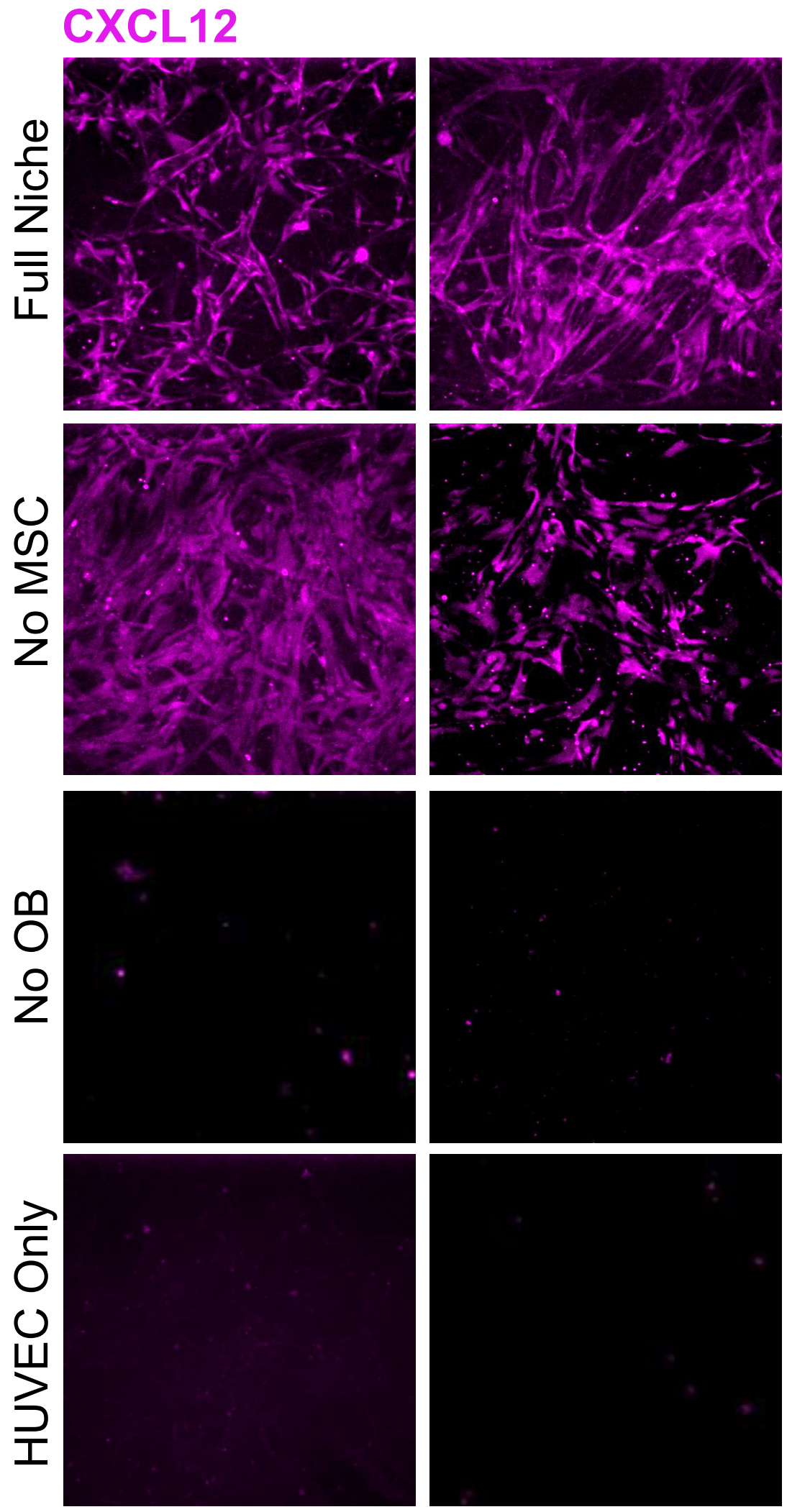

Supplemental Figure S4

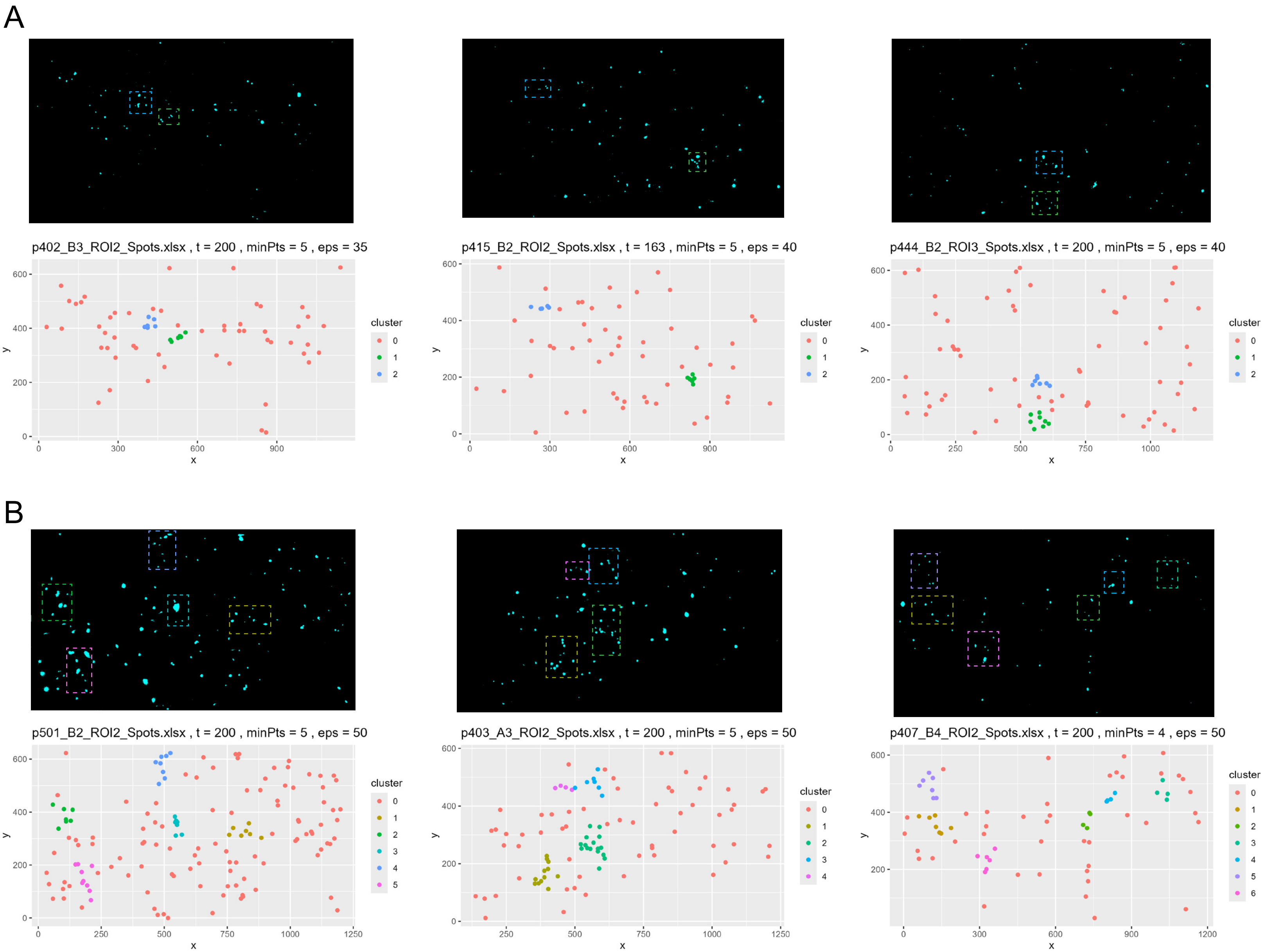

Other Supplementary Material

Data Fig 1, 2, 3, 4, 5, 6, S1 and S2

ALL DATA R CODE

```
#loading cleaning and filtering trackmate data
library(dplyr)
library(tidyverse)
library(readxl)
library(magrittr)
library(ggpmisc)
library(plotly)
library(fmsb)
library(ggradar)
library(reticulate)
library(plotrix)


#### Setup ####
key <- read_xlsx("Condition Key.xlsx")


#### read_Trackmate_Files function ####
read_Trackmate_Files <- function(nm) {
  #read in tracks file and rename columns
  rawTrack<-read_xlsx(paste(nm, "_Tracks.xlsx", sep = ""), col_names =
FALSE,
                      .name_repair = "unique_quiet")
  colnames(rawTrack)<- c("label", "index", "track ID", "N spots", "N
gaps", "N splits",
                         "N merges", "N complex", "lgst gap", "duration",
"track start",
                         "track stop", "track disp", "track x",
                         "track y", "track z", "mean sp", "max speed",
"min speed",
                         "med speed", "std speed", "mean q", "total dist",
"max dist",
                         "cfn ratio", "mn v line", "fwd progr", "mn y
rate")

  #read in spots file and rename columns
  rawSpots <- read_xlsx(paste(nm, "_Spots.xlsx", sep = ""), col_names =
FALSE,
                        .name_repair = "unique_quiet")
  colnames(rawSpots) <- c("label", "spot ID", "track ID", "quality", "x",
"y", "z",
                          "t", "frame", "r", "visibility", "spot color",
"mean ch1",
                          "median ch1", "min ch1", "max ch1", "sum ch1",
"std ch1",
                          "ctrst ch1", "snr ch1")

  # #read in edges file and rename columns
  # rawEdges <- read_xlsx(paste(nm, "_Edges.xlsx", sep = ""), col_names =
FALSE,
  #                       .name_repair = "unique_quiet")
```

```
  # colnames(rawEdges) <- c("label", "track ID", "source ID", "target ID",
"cost",
  #                          "y rate", "speed", "disp", "edge t", "edge x",
"edge y",
  #                          "edge z")

  #keeping only rows with more than 60 spots (frames)
  shortFilterTrack <- filter(rawTrack, `N spots` > 60)
  #matching spots from filtered tracks
  shortFilterSpots <- filter(rawSpots, `track ID` %in%
shortFilterTrack$`track ID`)
  # #matching edges from filtered tracks
  # shortFilterEdges <- filter(rawEdges, `track ID` %in%
shortFilterTrack$`track ID`)

  # making groups by track displacement
  # lowDispTrack <- filter(shortFilterTrack, `track disp` < 10)
  # medDispTrack <- filter(shortFilterTrack, `track disp` >= 10 & `track
disp` < 20)
  highDispTrack <- filter(shortFilterTrack, `track disp` >= 20)

  #matching spots from track disp groups
  # lowDispSpots <- filter(rawSpots, `track ID` %in% lowDispTrack$`track
ID`)
  # medDispSpots <- filter(rawSpots, `track ID` %in% medDispTrack$`track
ID`)
  highDispSpots <- filter(rawSpots, `track ID` %in% highDispTrack$`track
ID`)

  # #matching edges from track disp groups
  # # lowDispEdges <- filter(rawEdges, `track ID` %in% lowDispTrack$`track
ID`)
  # # medDispEdges <- filter(rawEdges, `track ID` %in% medDispTrack$`track
ID`)
  # highDispEdges <- filter(rawEdges, `track ID` %in% highDispTrack$`track
ID`)


  #start cell traces at origin
  highDispSpots <- mutate(highDispSpots, .by=`track ID`, normx = x-
x[which.min(t)][1L])
  highDispSpots <- mutate(highDispSpots, .by=`track ID`, normy = y-
y[which.min(t)][1L])

  #sort data by time (ascending) by track
  highDispSpots_sorted <- highDispSpots %>% group_by(`track ID`) %>%
arrange(t, .by_group = T)

  #adding a column with the name of the track
  highDispSpots_sorted <- mutate(highDispSpots_sorted, filename = nm)

  # endpoint x and y values to highDispTrack from highDispSpots
  highDispSpots <- mutate(highDispSpots, .by=`track ID`, max_x =
normx[which.max(t)][1L])
```

```
  highDispSpots <- mutate(highDispSpots, .by=`track ID`, max_y =
normy[which.max(t)][1L])
  disthDS <- distinct(highDispSpots[c("track ID", "max_x", "max_y")],
`track ID`, .keep_all = TRUE)
  # keep unique values
  highDispTrack_end <- merge(highDispTrack, disthDS, by="track ID")

  #calculate and add theta values
  highDispTrack_end <- mutate(highDispTrack_end, theta = atan2(max_y,
max_x)*180/pi)

  #convert to intermediate theta values for star chart
  highDispTrack_end <- mutate(highDispTrack_end, int_theta = theta + 180)
  #add sector information
  highDispTrack_end <- mutate(highDispTrack_end, sector =
floor(int_theta/30))
  #force anything in sector 12 (int_theta==360) to sector 0
  highDispTrack_end$sector[highDispTrack_end$sector == 12] <- 0

  #add file name
  highDispTrack_end <- mutate(highDispTrack_end, name = nm)

  return(highDispTrack_end)
}

#### Apply function to all files and combine ####
allData <- data.frame()

for (i in 1:nrow(key)) {
  allData <- rbind(allData, read_Trackmate_Files(key$full[i]))
}

allData <- left_join(allData, key, by = c("name" = "full"))
allData$Condition <- as.factor(allData$Condition)

#### Star/Radar Plot for all conditions ####
#create table for star plot
  #count and mean displacement for each sector
starChart <- summarize(allData, .by = `sector`, count = n(),
                       mean = mean(`track disp`))

#order by sector ID
starChart <- arrange(starChart, sector)
#set NA to 0
starChart[is.na(starChart)] <- 0
starChart$realSector <- c(9, 8, 7, 6, 5, 4, 3, 2, 1, 12, 11, 10)
starPlot <- starChart[c("realSector", "count", "mean")]
starPlot <- arrange(starPlot, realSector)

## legend for later: real sector
#sector 1 = 60-90 degrees
#sector 2 = 30-60 degrees
#sector 3 = 0-30 degrees
#sector 4 = 330-360 degrees
```

```
#sector 5 = 300-330 degrees
#sector 6 = 270-300 degrees
#sector 7 = 240-270 degrees
#sector 8 = 210-240 degrees
#sector 9 = 180-210 degrees
#sector 10 = 150-180 degrees
#sector 11 = 120-150 degrees
#sector 12 = 90-120 degrees

#transpose and make sectors the column titles
starPlot <- setNames(as.data.frame(t(starPlot[-1])), starPlot[,1])
starPlot <- add_column(starPlot, group = c("count", "mean"), .before = 1)

# make star/radar chart for frequency
ggradar(
  starPlot[1,],
  values.radar = c("0", "50", "100"),
  grid.min = 0, grid.mid = 50, grid.max = 100,
  group.line.width = 1.2,
  group.point.size = 3.5,
  group.colours = "#1834a3",
  background.circle.colour = "white",
  gridline.mid.colour = "grey",
  plot.title = "Frequency"
)
ggsave("allData_frequency radar.png")

# make star/radar chart for avg displacement
ggradar(
  starPlot[2,],
  values.radar = c("0", "40", "75"),
  grid.min = 0, grid.mid = 40, grid.max = 75,
  group.line.width = 1.2,
  group.point.size = 3.5,
  group.colours = "#1834a3",
  background.circle.colour = "white",
  gridline.mid.colour = "grey",
  plot.title = "Average Displacement"
)
ggsave("allData_displacement radar.png")

#### Star/Radar Plot by condition ####
starChart_fullNiche <- filter(allData, Condition == "Full Niche")
starChart_CXCL12 <- filter(allData, Condition == "CXCL12")
starChart_noOB <- filter(allData, Condition == "No OB")


## Data Preparation for full niche condition
star_fullNiche <- summarize(starChart_fullNiche, .by = `sector`, count =
n(),
                       mean = mean(`track disp`))
#order by sector ID
star_fullNiche <- arrange(star_fullNiche, sector)
#set NA to 0
```

```
star_fullNiche[is.na(star_fullNiche)] <- 0
star_fullNiche$realSector <- c(9, 8, 7, 6, 5, 4, 3, 2, 1, 12, 11, 10)
starPlot_fullNiche <- star_fullNiche[c("realSector", "count", "mean")]
starPlot_fullNiche <- arrange(starPlot_fullNiche, realSector)
#transpose and make sectors the column titles
starPlot_fullNiche <- setNames(as.data.frame(t(starPlot_fullNiche[-1])),
starPlot_fullNiche[,1])


## Data Preparation for CXCL12 condition
star_CXCL12 <- summarize(starChart_CXCL12, .by = `sector`, count = n(),
                            mean = mean(`track disp`))
#order by sector ID
star_CXCL12 <- arrange(star_CXCL12, sector)
#set NA to 0
star_CXCL12[is.na(star_CXCL12)] <- 0
star_CXCL12$realSector <- c(9, 8, 7, 6, 5, 4, 3, 2, 1, 12, 11, 10)
starPlot_CXCL12 <- star_CXCL12[c("realSector", "count", "mean")]
starPlot_CXCL12 <- arrange(starPlot_CXCL12, realSector)
#transpose and make sectors the column titles
starPlot_CXCL12 <- setNames(as.data.frame(t(starPlot_CXCL12[-1])),
starPlot_CXCL12[,1])

## Data Preparation for noOB condition
star_noOB <- summarize(starChart_noOB, .by = `sector`, count = n(),
                        mean = mean(`track disp`))
#order by sector ID
star_noOB <- arrange(star_noOB, sector)
#set NA to 0
star_noOB[is.na(star_noOB)] <- 0
star_noOB$realSector <- c(9, 8, 7, 6, 5, 4, 3, 2, 1, 12, 11, 10)
starPlot_noOB <- star_noOB[c("realSector", "count", "mean")]
starPlot_noOB <- arrange(starPlot_noOB, realSector)
#transpose and make sectors the column titles
starPlot_noOB <- setNames(as.data.frame(t(starPlot_noOB[-1])),
starPlot_noOB[,1])

#compile all conditions to plot - frequency
starFreq <- rbind(starPlot_fullNiche[1,], starPlot_CXCL12[1,],
starPlot_noOB[1,])
row.names(starFreq) <- c("Full Niche", "CXCL12", "No OB")

# convert freq to %
starCount <- rowSums(starFreq)
starPerc <- (starFreq/starCount)*100

starFreq <- add_column(starFreq, group = c("Full Niche", "CXCL12", "No
OB"), .before = 1)
starPerc <- add_column(starPerc, group = c("Full Niche", "CXCL12", "No
OB"), .before = 1)

#set color scheme
colors_star <- c("#E69F00", "#56B4E9", "#009E73")
```

```
ggradar(
  starFreq,
  values.radar = c("0", "25", "50"),
  grid.min = 0, grid.mid = 25, grid.max = 50,
  group.line.width = 1.2,
  group.point.size = 3.5,
  background.circle.colour = "white",
  gridline.mid.colour = "grey",
  group.colours = colors_star,
  plot.title = "Frequency"
)

ggradar(
  starPerc,
  values.radar = c("0", "5", "15"),
  grid.min = 0, grid.mid = 5, grid.max = 15,
  group.line.width = 1.2,
  group.point.size = 3.5,
  background.circle.colour = "white",
  gridline.mid.colour = "grey",
  group.colours = colors_star,
  plot.title = "Frequency (%)"
)




#compile all conditions to plot - displacement
starDisp <- rbind(starPlot_fullNiche[2,], starPlot_CXCL12[2,],
starPlot_noOB[2,])
starDisp <- add_column(starDisp, group = c("Full Niche", "CXCL12", "No
OB"), .before = 1)
starDisp[,2:13] <- starDisp[,2:13]*1.612

ggradar(
  starDisp,
  values.radar = c("0", "60", "120"),
  grid.min = 0, grid.mid = 60, grid.max = 120,
  group.line.width = 1.2,
  group.point.size = 3.5,
  background.circle.colour = "white",
  gridline.mid.colour = "grey",
  group.colours = colors_star,
  plot.title = "Avg Displacement"
)

names(starDisp) <- c("group", "90", "60", "30", "0","330", "300", "270",
"240", "210", "180", "150", "120")

# to graph only Full Niche (first in the list)
ggradar(
  starDisp[1,],
  values.radar = c("0 um", "60 um", "120 um"),
  grid.min = 0, grid.mid = 60, grid.max = 120,
```

```
  group.line.width = 1.2,
  group.point.size = 0,
  background.circle.colour = "white",
  gridline.mid.colour = "grey",
  grid.label.size = 4,
  axis.label.size = 6
)
```

Description of Code Functions

1. AnalyzeData2 took the csv files from ImageJ and converted them into a combined xlsx file
2. CompileData combined all the data for all devices in a condition
3. SortAndFilter removed data points with nSpots < 60 and reorganized the data layout in the sheets to make copying and pasting into GraphPad easier
4. TrackDispFilter organized data into the low medium and high displacement groups for data points with nSpots < 60
5. Percentages calculated % of cells in each motility group for each condition

Analyze Data 2 MATLAB Code

```
function [Data] = analyzeData2(projectdir)
%must have a FOLDER of the .csv files for the experiment group in the
%CURRENT FOLDER in MATLAB
%must also have an excel file names 'ImageJ Count Analysis Summary.xlsx"
in
%the CURRENT FOLDER in MATLAB
%when running the code, type the exact name of the file with the data you
%want to analyze in it

allFiles = dir(projectdir); %ST with all the files
allFiles(1:2) = []; %removes the first 2 rows of the ST because they do
not contain data
L1 = length(allFiles); %number of files in folder
plateCA = {'Device ID'}; %CA of plates and device IDs so far

%writing the titiles "Device ID" and "Time Point" to each sheet in the
spreadsheet
writecell(plateCA,'ImageJ Count Analysis Summary.xlsx','Sheet','avg
count','Range','A1');
writecell(plateCA,'ImageJ Count Analysis Summary.xlsx','Sheet','avg
area','Range','A1');
writecell(plateCA,'ImageJ Count Analysis Summary.xlsx','Sheet','sum
area','Range','A1');
writecell(plateCA,'ImageJ Count Analysis Summary.xlsx','Sheet','avg
intensity (mean)','Range','A1');
writecell(plateCA,'ImageJ Count Analysis Summary.xlsx','Sheet','avg
intensity (median)','Range','A1');

writecell({'Time Point'},'ImageJ Count Analysis Summary.xlsx','Sheet','avg
count','Range','A3');
```

```
writecell({'Time Point'},'ImageJ Count Analysis Summary.xlsx','Sheet','avg
area','Range','A3');
writecell({'Time Point'},'ImageJ Count Analysis Summary.xlsx','Sheet','sum
area','Range','A3');
writecell({'Time Point'},'ImageJ Count Analysis Summary.xlsx','Sheet','avg
intensity (mean)','Range','A3');
writecell({'Time Point'},'ImageJ Count Analysis Summary.xlsx','Sheet','avg
intensity (median)','Range','A3');

%looping though the entire list of filenames
for z = 1:L1

    FN = allFiles(z).name; %get the specific file name as a string
    plate = strfind(FN,'p'); %locate plate and device ID in filename
    conds = strfind(FN,'_'); %vec of locations of different information in
the filename
    infoAmount = length(conds); %how much info is in filename

    plate = FN(plate:plate+6); %isolate plate ID and device ID (hardcoded
for plate IDs 3 digits long)

    L3 = length(plateCA); %how long CA of device names is so far
    if ~strcmp(plate,plateCA{L3}); %if it doesn't find device ID, it will
add it to a new column (hardcoded for numerical order)

         D = char(65+L3); %coulmn to be added to
         locB = [D '1']; %column and row position to be added to
(hardcoded to be added to the first row always)
         plate = {plate};

         writecell(plate,'ImageJ Count Analysis Summary.xlsx','Sheet','avg
count','Range',locB);
         writecell(plate,'ImageJ Count Analysis Summary.xlsx','Sheet','avg
area','Range',locB);
         writecell(plate,'ImageJ Count Analysis Summary.xlsx','Sheet','sum
area','Range',locB);
         writecell(plate,'ImageJ Count Analysis Summary.xlsx','Sheet','avg
intensity (mean)','Range',locB);
         writecell(plate,'ImageJ Count Analysis Summary.xlsx','Sheet','avg
intensity (median)','Range',locB);

         plateCA = [plateCA plate]; %updated plate CA with new plate to
update L3 as well

    end


    if infoAmount > 3 %pull and add the testing condition of the device
from the filename if they are in it

        conds1 = FN(conds(3)+1);
        conds2 = FN(conds(4)-1);
        conds = [conds1 conds2];
        conds = {conds};
```

```
        locC = [D '2'];

        writecell(conds,'ImageJ Count Analysis Summary.xlsx','Sheet','avg
count','Range',locC);
        writecell(conds,'ImageJ Count Analysis Summary.xlsx','Sheet','avg
area','Range',locC);
        writecell(conds,'ImageJ Count Analysis Summary.xlsx','Sheet','sum
area','Range',locC);
        writecell(conds,'ImageJ Count Analysis Summary.xlsx','Sheet','avg
intensity (mean)','Range',locC);
        writecell(conds,'ImageJ Count Analysis Summary.xlsx','Sheet','avg
intensity (median)','Range',locC);

    end

    z = z+1;

end

plateCA(1) = []; %delete title "Device ID" from CA of device IDs
L4 = length(plateCA); %how many different devices are in the file
zro = num2cell(zeros(1,L4));
days = [plateCA; zro]; %initializing counter for how many days of imaging
were done per device

%counting how many days of imagine were done for each device
for y = 1:L1;

    FN = allFiles(y).name; %get the specific file name
    plate2 = strfind(FN,'p'); %locate plate and deveice ID in filename
    plate2 = FN(plate2:plate2+6); %isolate plate ID and device ID

    for w = 1:L4;

        if strcmp(plate2,plateCA{w});
            days {2,w} = days{2,w} + 1; %increase count of how many days
of imagine were done
        end

        w = w+1;

    end

    y = y+1;

end

days = days;

for x = 1:L1; %for each file one at a time

    FN = allFiles(x).name; %get the specific file name
    file = fullfile(projectdir,FN); %open specific file
    ca = readtable(file); %read the file and make it a table
```

```
    ca = table2cell(ca); %turn table into a CA with columns of data
    ID = file; %just file name to get time from
    ca(:,1:2) = []; %remove number and label
    [r,~] = size(ca); %AVG COUNT VALUE
    num = cell2mat(ca); %array of data for the open file

    %removing data with area value > 1000 (threshold can be changed)
    areaValues = num(:,1); %taking just the area values as a vector
    mask = areaValues > 1000; %creating a mask of all the places the area
value is greather than the threshold
    %remove '%' below to print the area values of the removed data
    %removedValues = num(mask)
    num(mask,:) = []; %using mask to remove the data for all counts with
areas above threshold

    [r2,~] = size(num);
    newCA = {};
    newCA{1} = r2;

    avgs = mean (num); %array of column data averages
    avgs(3:6) = []; %delete averages  we don't input
    sumArea = sum(num(:,1)); %SUM AREA VALUE
    newCA = [newCA avgs(1) sumArea avgs(2) avgs(3)]; %CA of [avg count,
avg area, sum area, ...] that we want to print to the spreadsheet

    loc = strfind(ID,'t0');
    time = ID(loc+1:loc+3);
    time = str2num(time); %timepoint taken at (hardcoded for 3 digit time
measurements)
    plate3 = strfind(FN,'p'); %locate plate and deveice ID in filename
    plate3 = FN(plate3:plate3+6); %isolate plate ID and device

    for h=1:L4 %for the number of different devices

        if strcmp(plate3,plateCA{h})

            d = days{2,h}; %how many days of imiging were done for the
deivice
            locD = h; %were along the CA this device is so we know what
column to print data into

        end

        h = h+1;

    end

    A = 65 + locD; %location of device column for open file
    B = char(A);
    AA = num2str(time + 3); %location of device and timepoint of openfile
    cellLoc = [B AA];
    writecell(newCA(1),'ImageJ Count Analysis Summary.xlsx','Sheet','avg
count','Range',cellLoc);
```

```
    writecell(newCA(2),'ImageJ Count Analysis Summary.xlsx','Sheet','avg
area','Range',cellLoc);
    writecell(newCA(3),'ImageJ Count Analysis Summary.xlsx','Sheet','sum
area','Range',cellLoc);
    writecell(newCA(4),'ImageJ Count Analysis Summary.xlsx','Sheet','avg
intensity (mean)','Range',cellLoc);
    writecell(newCA(5),'ImageJ Count Analysis Summary.xlsx','Sheet','avg
intensity (median)','Range',cellLoc);


%     if time == 1 %time in filename
%
%         A = 65 + locD; %location of device column for open file
%         B = char(A);
%         cellLoc = [B '4']; %location of device and timepoint of openfile
%
%         %printing avg and sum values into the spreadsheat at specific
%         %location
%         writecell(newCA(1),'ImageJ Count Analysis
Summary.xlsx','Sheet','avg count','Range',cellLoc);
%         writecell(newCA(2),'ImageJ Count Analysis
Summary.xlsx','Sheet','avg area','Range',cellLoc);
%         writecell(newCA(3),'ImageJ Count Analysis
Summary.xlsx','Sheet','sum area','Range',cellLoc);
%         writecell(newCA(4),'ImageJ Count Analysis
Summary.xlsx','Sheet','avg intensity (mean)','Range',cellLoc);
%         writecell(newCA(5),'ImageJ Count Analysis
Summary.xlsx','Sheet','avg intensity (median)','Range',cellLoc);
%
%     elseif time == 2;
%
%         A = 65 + locD;
%         B = char(A);
%         cellLoc = [B '5'];
%
%         writecell(newCA(1),'ImageJ Count Analysis
Summary.xlsx','Sheet','avg count','Range',cellLoc);
%         writecell(newCA(2),'ImageJ Count Analysis
Summary.xlsx','Sheet','avg area','Range',cellLoc);
%         writecell(newCA(3),'ImageJ Count Analysis
Summary.xlsx','Sheet','sum area','Range',cellLoc);
%         writecell(newCA(4),'ImageJ Count Analysis
Summary.xlsx','Sheet','avg intensity (mean)','Range',cellLoc);
%         writecell(newCA(5),'ImageJ Count Analysis
Summary.xlsx','Sheet','avg intensity (median)','Range',cellLoc);
%
%     end

    x = x+1;

end

table1 = table2cell(readtable("ImageJ Count Analysis
Summary.xlsx","Sheet","avg count"));
```

```
table2 = table2cell(readtable("ImageJ Count Analysis
Summary.xlsx","Sheet","avg area"));
table3 = table2cell(readtable("ImageJ Count Analysis
Summary.xlsx","Sheet","sum area"));
table4 = table2cell(readtable("ImageJ Count Analysis
Summary.xlsx","Sheet","avg intensity (mean)"));
table5 = table2cell(readtable("ImageJ Count Analysis
Summary.xlsx","Sheet","avg intensity (median)"));


table1(1,:) = []; %may have to be 1:2,: depending on how the table is read
table1(:,1) = [];
table2(1,:) = [];
table2(:,1) = [];
table3(1,:) = [];
table3(:,1) = [];
table4(1,:) = [];
table4(:,1) = [];
table5(1,:) = [];
table5(:,1) = [];
% %taking the data and making ca of each page without headers

K = max([days{2,:}]); %max number of days of imagining done

%adding times to the sheet for the normalized values below the first set
of data
for v = 1:K

    E = num2str(K + v + 4);
    F = num2str(v + 3);
    cellLoc2 = ['A' E];
    cellLoc4 = ['A' F];

    writecell({v},'ImageJ Count Analysis Summary.xlsx','Sheet','avg
count','Range',cellLoc4);
    writecell({v},'ImageJ Count Analysis Summary.xlsx','Sheet','avg
area','Range',cellLoc4);
    writecell({v},'ImageJ Count Analysis Summary.xlsx','Sheet','sum
area','Range',cellLoc4);
    writecell({v},'ImageJ Count Analysis Summary.xlsx','Sheet','avg
intensity (mean)','Range',cellLoc4);
    writecell({v},'ImageJ Count Analysis Summary.xlsx','Sheet','avg
intensity (median)','Range',cellLoc4);


    writecell({v},'ImageJ Count Analysis Summary.xlsx','Sheet','avg
count','Range',cellLoc2);
    writecell({v},'ImageJ Count Analysis Summary.xlsx','Sheet','avg
area','Range',cellLoc2);
    writecell({v},'ImageJ Count Analysis Summary.xlsx','Sheet','sum
area','Range',cellLoc2);
    writecell({v},'ImageJ Count Analysis Summary.xlsx','Sheet','avg
intensity (mean)','Range',cellLoc2);
```

```
    writecell({v},'ImageJ Count Analysis Summary.xlsx','Sheet','avg
intensity (median)','Range',cellLoc2);

    v = v + 1;

end

[r2,c2] = size(table1); %size of the data

locH = ['A' num2str(r2+4)];
writecell({'Normalized Values'},'ImageJ Count Analysis
Summary.xlsx','Sheet','avg count','Range',locH);
writecell({'Normalized Values'},'ImageJ Count Analysis
Summary.xlsx','Sheet','avg area','Range',locH);
writecell({'Normalized Values'},'ImageJ Count Analysis
Summary.xlsx','Sheet','sum area','Range',locH);
writecell({'Normalized Values'},'ImageJ Count Analysis
Summary.xlsx','Sheet','avg intensity (mean)','Range',locH);
writecell({'Normalized Values'},'ImageJ Count Analysis
Summary.xlsx','Sheet','avg intensity (median)','Range',locH);


x4 = 1;
while x4 <= c2

    normValues1 = [table1{:,x4}]./table1{1,x4};
    normValues1 = num2cell(normValues1);
    normValues2 = [table2{:,x4}]./table2{1,x4};
    normValues2 = num2cell(normValues2);
    normValues3 = [table3{:,x4}]./table3{1,x4};
    normValues3 = num2cell(normValues3);
    normValues4 = [table4{:,x4}]./table4{1,x4};
    normValues4 = num2cell(normValues4);
    normValues5 = [table5{:,x4}]./table5{1,x4};
    normValues5 = num2cell(normValues5);
    LocE = char(65+x4);

    x5 = 1;
    while x5 <= r2

        locG = [LocE num2str(x5+r2+4)];
        writecell(normValues1(x5),'ImageJ Count Analysis
Summary.xlsx','Sheet','avg count','Range',locG);
        writecell(normValues2(x5),'ImageJ Count Analysis
Summary.xlsx','Sheet','avg area','Range',locG);
        writecell(normValues3(x5),'ImageJ Count Analysis
Summary.xlsx','Sheet','sum area','Range',locG);
        writecell(normValues4(x5),'ImageJ Count Analysis
Summary.xlsx','Sheet','avg intensity (mean)','Range',locG);
        writecell(normValues5(x5),'ImageJ Count Analysis
Summary.xlsx','Sheet','avg intensity (median)','Range',locG);

        x5 = x5+1;
```

```
    end

    x4 = x4+1;

end

disp('Done!')

end
```

Compile Data MATLAB Code

```
function [Data] = compileData(projectdir)
%must have a FOLDER of the .xlsx files for the data to compile in the
%CURRENT FOLDER in MATLAB
%must also have an excel file named '(Folder condition)_Compiled.xlsx" in
%the CURRENT FOLDER in MATLAB
%when running the code, type the exact name of the file with the data you
%want to analyze in it

allFiles = dir(projectdir); %ST with all the files
allFiles(1:2) = []; %removes the first 2 rows of the ST because they do
not contain data

DataCA = {}; %create cell aray of data to write to the xlsx file
plates = {};
L1 = length(allFiles); % # of devices in the condition

for x1 = 1:L1

    FN = allFiles(x1).name; %get the specific file name as a string
    FN = FN(1:end-5);
    DataCA = {FN};
    L2 = length(plates);

    Page1Col = char(65+L2*2);
    Page1Col2 = char(66+L2*2);
    loc1 = [Page1Col '1'];
    loc2 = [Page1Col '2'];
    loc3 = [Page1Col2 '2'];

    writecell(DataCA,'Compiled.xlsx','Sheet','Track Disp','Range',loc1);
    writecell({'N spots'},'Compiled.xlsx','Sheet','Track
Disp','Range',loc2);
    writecell({'Track Disp'},'Compiled.xlsx','Sheet','Track
Disp','Range',loc3);

    Page2Col = (65+L2*3);
    Page2Col2 = (66+L2*3);
    Page2Col3 = (67+L2*3);

    if Page2Col > 90
        D = char(Page2Col-90+64);
```

```
        loc5 = ['A' D '2'];
        loc4 = ['A' D '1'];
    else
        loc4 = [Page2Col '1'];
        loc5 = [Page2Col '2'];
    end

    if Page2Col2 > 90
        C = char(Page2Col2-90+64);
        loc6 = ['A' C '2'];
    else
         loc6 = [Page2Col2 '2'];
    end

    if Page2Col3 > 90
        A = char(Page2Col3-90+64);
        loc7 = ['A' A '2'];
    else
        loc7 = [Page2Col3 '2'];
    end

    writecell(DataCA,'Compiled.xlsx','Sheet','Mean Sp','Range',loc4);
    writecell({'N spots'},'Compiled.xlsx','Sheet','Mean Sp','Range',loc5);
    writecell({'Track Disp'},'Compiled.xlsx','Sheet','Mean
Sp','Range',loc6);
    writecell({'Data'},'Compiled.xlsx','Sheet','Mean Sp','Range',loc7);

    writecell(DataCA,'Compiled.xlsx','Sheet','Median Sp','Range',loc4);
    writecell({'N spots'},'Compiled.xlsx','Sheet','Median
Sp','Range',loc5);
    writecell({'Track Disp'},'Compiled.xlsx','Sheet','Median
Sp','Range',loc6);
    writecell({'Data'},'Compiled.xlsx','Sheet','Median Sp','Range',loc7);

    writecell(DataCA,'Compiled.xlsx','Sheet','Total Dist','Range',loc4);
    writecell({'N spots'},'Compiled.xlsx','Sheet','Total
Dist','Range',loc5);
    writecell({'Track Disp'},'Compiled.xlsx','Sheet','Total
Dist','Range',loc6);
    writecell({'Data'},'Compiled.xlsx','Sheet','Total Dist','Range',loc7);

    writecell(DataCA,'Compiled.xlsx','Sheet','Max Dist','Range',loc4);
    writecell({'N spots'},'Compiled.xlsx','Sheet','Max
Dist','Range',loc5);
    writecell({'Track Disp'},'Compiled.xlsx','Sheet','Max
Dist','Range',loc6);
    writecell({'Data'},'Compiled.xlsx','Sheet','Max Dist','Range',loc7);

    writecell(DataCA,'Compiled.xlsx','Sheet','Confin Ratio','Range',loc4);
    writecell({'N spots'},'Compiled.xlsx','Sheet','Confin
Ratio','Range',loc5);
    writecell({'Track Disp'},'Compiled.xlsx','Sheet','Confin
Ratio','Range',loc6);
```

```
    writecell({'Data'},'Compiled.xlsx','Sheet','Confin
Ratio','Range',loc7);

    writecell(DataCA,'Compiled.xlsx','Sheet','Mean Straightline
Speed','Range',loc4);
    writecell({'N spots'},'Compiled.xlsx','Sheet','Mean Straightline
Speed','Range',loc5);
    writecell({'Track Disp'},'Compiled.xlsx','Sheet','Mean Straightline
Speed','Range',loc6);
    writecell({'Data'},'Compiled.xlsx','Sheet','Mean Straightline
Speed','Range',loc7);

    writecell(DataCA,'Compiled.xlsx','Sheet','Fwd Prog','Range',loc4);
    writecell({'N spots'},'Compiled.xlsx','Sheet','Fwd
Prog','Range',loc5);
    writecell({'Track Disp'},'Compiled.xlsx','Sheet','Fwd
Prog','Range',loc6);
    writecell({'Data'},'Compiled.xlsx','Sheet','Fwd Prog','Range',loc7);

    writecell(DataCA,'Compiled.xlsx','Sheet','Mean Y Rate','Range',loc4);
    writecell({'N spots'},'Compiled.xlsx','Sheet','Mean Y
Rate','Range',loc5);
    writecell({'Track Disp'},'Compiled.xlsx','Sheet','Mean Y
Rate','Range',loc6);
    writecell({'Data'},'Compiled.xlsx','Sheet','Mean Y
Rate','Range',loc7);

    plates = [plates DataCA];

    FN = allFiles(x1).name; %get the specific file name
    file = fullfile(projectdir,FN); %open specific file
    ca = readtable(file); %read the file and make it a table
    ca = table2cell(ca); %turn table into a CA with columns of data
    nSpots = ca(:,4);
    [L2,~] = size(nSpots);
    B = num2str(L2+3);
    loc8 = [Page1Col '3:' Page1Col B];

    writecell(nSpots,'Compiled.xlsx','Sheet','Track Disp','Range',loc8);

    if Page2Col > 90
        C = char(Page2Col-90+64);
        loc9 = ['A' C '3:' 'A' C B];
    else
        loc9 = [Page2Col '3:' Page2Col B];
    end

    writecell(nSpots,'Compiled.xlsx','Sheet','Mean Sp','Range',loc9);
    writecell(nSpots,'Compiled.xlsx','Sheet','Median Sp','Range',loc9);
    writecell(nSpots,'Compiled.xlsx','Sheet','Total Dist','Range',loc9);
    writecell(nSpots,'Compiled.xlsx','Sheet','Max Dist','Range',loc9);
    writecell(nSpots,'Compiled.xlsx','Sheet','Confin Ratio','Range',loc9);
    writecell(nSpots,'Compiled.xlsx','Sheet','Mean Straightline
Speed','Range',loc9);
```

```
    writecell(nSpots,'Compiled.xlsx','Sheet','Fwd Prog','Range',loc9);
    writecell(nSpots,'Compiled.xlsx','Sheet','Mean Y Rate','Range',loc9);

    trackDisp = ca(:,13);
    if Page1Col2 > 90
        E = char(Page1Col2-90+64);
        loc10 = ['A' E '3:' 'A' E B];
    else
        loc10 = [Page1Col2 '3:' Page1Col2 B];
    end

    writecell(trackDisp,'Compiled.xlsx','Sheet','Track
Disp','Range',loc10);

    if Page2Col2 > 90
        F = char(Page2Col2-90+64);
        loc11 = ['A' F '3:' 'A' F B];
    else
        loc11 = [Page2Col2 '3:' Page2Col2 B];
    end

    writecell(trackDisp,'Compiled.xlsx','Sheet','Mean Sp','Range',loc11);
    writecell(trackDisp,'Compiled.xlsx','Sheet','Median
Sp','Range',loc11);
    writecell(trackDisp,'Compiled.xlsx','Sheet','Total
Dist','Range',loc11);
    writecell(trackDisp,'Compiled.xlsx','Sheet','Max Dist','Range',loc11);
    writecell(trackDisp,'Compiled.xlsx','Sheet','Confin
Ratio','Range',loc11);
    writecell(trackDisp,'Compiled.xlsx','Sheet','Mean Straightline
Speed','Range',loc11);
    writecell(trackDisp,'Compiled.xlsx','Sheet','Fwd Prog','Range',loc11);
    writecell(trackDisp,'Compiled.xlsx','Sheet','Mean Y
Rate','Range',loc11);

    if Page2Col3 > 90;
        A = char(Page2Col3-90+64);
        loc12 = [A 'A'];
        loc12 = [loc12 '3:' loc12 B];
    else
        loc12 = [Page2Col3 '3:' Page2Col3 B];
    end

    meanSp = ca(:,17);
    writecell(meanSp,'Compiled.xlsx','Sheet','Mean Sp','Range',loc12);

    medianSp = ca(:,20);
    writecell(medianSp,'Compiled.xlsx','Sheet','Median Sp','Range',loc12);

    totalDist = ca(:,23);
    writecell(totalDist,'Compiled.xlsx','Sheet','Total
Dist','Range',loc12);

    maxDist = ca(:,24);
```

```
    writecell(maxDist,'Compiled.xlsx','Sheet','Max Dist','Range',loc12);

    confinRatio = ca(:,25);
    writecell(confinRatio,'Compiled.xlsx','Sheet','Confin
Ratio','Range',loc12);

    MSS = ca(:,26);
    writecell(MSS,'Compiled.xlsx','Sheet','Mean Straightline
Speed','Range',loc12);

    fwdProg = ca(:,27);
    writecell(fwdProg,'Compiled.xlsx','Sheet','Fwd Prog','Range',loc12);

    MYR = ca(:,28);
    writecell(MYR,'Compiled.xlsx','Sheet','Mean Y Rate','Range',loc12);

end

disp('Done')

end
```

Sort and Filter MATLAB Code ____

```
function [Data] = SortAndFilter(projectdir)

allFiles = dir(projectdir); %ST with all the files
allFiles(1:2) = []; %removes the first 2 rows of the ST because they do
not contain data

L1 = length(allFiles); % # of devices in the condition

DataCA = {}; %create cell aray of data to write to the xlsx file
plates = {};
totalLengths = [0];

    writecell({'N spots'},'Sorted.xlsx','Sheet','Track
Disp','Range','A1');
    writecell({'N spots'},'Sorted.xlsx','Sheet','Mean Sp','Range','A1');
    writecell({'N spots'},'Sorted.xlsx','Sheet','Median Sp','Range','A1');
    writecell({'N spots'},'Sorted.xlsx','Sheet','Total
Dist','Range','A1');
    writecell({'N spots'},'Sorted.xlsx','Sheet','Max Dist','Range','A1');
    writecell({'N spots'},'Sorted.xlsx','Sheet','Confin
Ratio','Range','A1');
    writecell({'N spots'},'Sorted.xlsx','Sheet','Mean Straightline
Speed','Range','A1');
    writecell({'N spots'},'Sorted.xlsx','Sheet','Fwd Prog','Range','A1');
    writecell({'N spots'},'Sorted.xlsx','Sheet','Mean Y
Rate','Range','A1');

    writecell({'Track Disp'},'Sorted.xlsx','Sheet','Track
Disp','Range','B1');
```

```
    writecell({'Track Disp'},'Sorted.xlsx','Sheet','Mean
Sp','Range','B1');
    writecell({'Track Disp'},'Sorted.xlsx','Sheet','Median
Sp','Range','B1');
    writecell({'Track Disp'},'Sorted.xlsx','Sheet','Total
Dist','Range','B1');
    writecell({'Track Disp'},'Sorted.xlsx','Sheet','Max
Dist','Range','B1');
    writecell({'Track Disp'},'Sorted.xlsx','Sheet','Confin
Ratio','Range','B1');
    writecell({'Track Disp'},'Sorted.xlsx','Sheet','Mean Straightline
Speed','Range','B1');
    writecell({'Track Disp'},'Sorted.xlsx','Sheet','Fwd
Prog','Range','B1');
    writecell({'Track Disp'},'Sorted.xlsx','Sheet','Mean Y
Rate','Range','B1');

    writecell({'Data'},'Sorted.xlsx','Sheet','Mean Sp','Range','C1');
    writecell({'Data'},'Sorted.xlsx','Sheet','Median Sp','Range','C1');
    writecell({'Data'},'Sorted.xlsx','Sheet','Total Dist','Range','C1');
    writecell({'Data'},'Sorted.xlsx','Sheet','Max Dist','Range','C1');
    writecell({'Data'},'Sorted.xlsx','Sheet','Confin Ratio','Range','C1');
    writecell({'Data'},'Sorted.xlsx','Sheet','Mean Straightline
Speed','Range','C1');
    writecell({'Data'},'Sorted.xlsx','Sheet','Fwd Prog','Range','C1');
    writecell({'Data'},'Sorted.xlsx','Sheet','Mean Y Rate','Range','C1');

for x1 = 1:L1

    FN = allFiles(x1).name; %get the specific file name as a string
    PlateName = FN(1:end-5); %Get plate ID
    DataCA = {PlateName};
    L2 = length(plates) + 1; %number of plates

    file = fullfile(projectdir,FN); %open specific file
    ca = readtable(file); %read the file and make it a table
    ca = table2cell(ca); %turn table into a CA with columns of data
    nSpots = ca(:,4);
    trackDisp = ca(:,13);
    meanSp = ca(:,17);
    medianSp = ca(:,20);
    totalDist = ca(:,23);
    maxDist = ca(:,24);
    confinRatio = ca(:,25);
    MSS = ca(:,26);
    fwdProg = ca(:,27);
    MYR = ca(:,28);
    allData = [nSpots trackDisp meanSp medianSp totalDist maxDist
confinRatio MSS fwdProg MYR];
    [L3,~] = size(nSpots); %number of datapoints for the plate

    for x2 = L3:-1:1

        if nSpots{x2} < 59
```

```
            allData(x2,:) = [];
        end

    end

    [L4,~] = size(allData);

    start = num2str(sum(totalLengths)+2);
    totalLengths = [totalLengths L4];

    %loc1 = num2str(totalLengths(L2) + 2)
    PlateIDLoc = ['D' start];
    nSpotsLoc = ['A' start];
    TDLoc = ['B' start];
    dataLoc = ['C' start];

    nSpots = allData(:,1);
    trackDisp = allData(:,2);
    meanSp = allData(:,3);
    medianSp = allData(:,4);
    totalDist = allData(:,5);
    maxDist = allData(:,6);
    confinRatio = allData(:,7);
    MSS = allData(:,8);
    fwdProg = allData(:,9);
    MYR = allData(:,10);

    writecell(DataCA,'Sorted.xlsx','Sheet','Track
Disp','Range',PlateIDLoc);
    writecell(DataCA,'Sorted.xlsx','Sheet','Mean Sp','Range',PlateIDLoc);
    writecell(DataCA,'Sorted.xlsx','Sheet','Median
Sp','Range',PlateIDLoc);
    writecell(DataCA,'Sorted.xlsx','Sheet','Total
Dist','Range',PlateIDLoc);
    writecell(DataCA,'Sorted.xlsx','Sheet','Max Dist','Range',PlateIDLoc);
    writecell(DataCA,'Sorted.xlsx','Sheet','Confin
Ratio','Range',PlateIDLoc);
    writecell(DataCA,'Sorted.xlsx','Sheet','Mean Straightline
Speed','Range',PlateIDLoc);
    writecell(DataCA,'Sorted.xlsx','Sheet','Fwd Prog','Range',PlateIDLoc);
    writecell(DataCA,'Sorted.xlsx','Sheet','Mean Y
Rate','Range',PlateIDLoc);

    writecell(nSpots,'Sorted.xlsx','Sheet','Track
Disp','Range',nSpotsLoc);
    writecell(nSpots,'Sorted.xlsx','Sheet','Mean Sp','Range',nSpotsLoc);
    writecell(nSpots,'Sorted.xlsx','Sheet','Median Sp','Range',nSpotsLoc);
    writecell(nSpots,'Sorted.xlsx','Sheet','Total
Dist','Range',nSpotsLoc);
    writecell(nSpots,'Sorted.xlsx','Sheet','Max Dist','Range',nSpotsLoc);
    writecell(nSpots,'Sorted.xlsx','Sheet','Confin
Ratio','Range',nSpotsLoc);
    writecell(nSpots,'Sorted.xlsx','Sheet','Mean Straightline
Speed','Range',nSpotsLoc);
```

```
    writecell(nSpots,'Sorted.xlsx','Sheet','Fwd Prog','Range',nSpotsLoc);
    writecell(nSpots,'Sorted.xlsx','Sheet','Mean Y
Rate','Range',nSpotsLoc);

    writecell(trackDisp,'Sorted.xlsx','Sheet','Track Disp','Range',TDLoc);
    writecell(trackDisp,'Sorted.xlsx','Sheet','Mean Sp','Range',TDLoc);
    writecell(trackDisp,'Sorted.xlsx','Sheet','Median Sp','Range',TDLoc);
    writecell(trackDisp,'Sorted.xlsx','Sheet','Total Dist','Range',TDLoc);
    writecell(trackDisp,'Sorted.xlsx','Sheet','Max Dist','Range',TDLoc);
    writecell(trackDisp,'Sorted.xlsx','Sheet','Confin
Ratio','Range',TDLoc);
    writecell(trackDisp,'Sorted.xlsx','Sheet','Mean Straightline
Speed','Range',TDLoc);
    writecell(trackDisp,'Sorted.xlsx','Sheet','Fwd Prog','Range',TDLoc);
    writecell(trackDisp,'Sorted.xlsx','Sheet','Mean Y
Rate','Range',TDLoc);

    writecell(meanSp,'Sorted.xlsx','Sheet','Mean Sp','Range',dataLoc);
    writecell(medianSp,'Sorted.xlsx','Sheet','Median Sp','Range',dataLoc);
    writecell(totalDist,'Sorted.xlsx','Sheet','Total
Dist','Range',dataLoc);
    writecell(maxDist,'Sorted.xlsx','Sheet','Max Dist','Range',dataLoc);
    writecell(confinRatio,'Sorted.xlsx','Sheet','Confin
Ratio','Range',dataLoc);
    writecell(MSS,'Sorted.xlsx','Sheet','Mean Straightline
Speed','Range',dataLoc);
    writecell(fwdProg,'Sorted.xlsx','Sheet','Fwd Prog','Range',dataLoc);
    writecell(MYR,'Sorted.xlsx','Sheet','Mean Y Rate','Range',dataLoc);

    plates = [plates DataCA];

end

disp('Done')

end
```

Track Disp Filter MATLAB Code

```
function [Data] = TrackDispFilter(projectdir)

allFiles = dir(projectdir); %ST with all the files
allFiles(1:3) = []; %removes the first 2 rows of the ST because they do
not contain data

L1 = length(allFiles); % # of devices in the condition

DataCA = {}; %create cell aray of data to write to the xlsx file
plates = {};
totalLengths = [0];

writecell({'low'},'Sorted.xlsx','Sheet','Track Disp','Range','A1');
writecell({'low'},'Sorted.xlsx','Sheet','Mean Sp','Range','A1');
writecell({'low'},'Sorted.xlsx','Sheet','Max Sp','Range','A1');
```

```
writecell({'low'},'Sorted.xlsx','Sheet','Total Dist','Range','A1');
writecell({'low'},'Sorted.xlsx','Sheet','Max Dist','Range','A1');
writecell({'low'},'Sorted.xlsx','Sheet','Confin Ratio','Range','A1');
writecell({'low'},'Sorted.xlsx','Sheet','Mean Straightline
Speed','Range','A1');
writecell({'low'},'Sorted.xlsx','Sheet','Fwd Prog','Range','A1');
writecell({'low'},'Sorted.xlsx','Sheet','Mean Y Rate','Range','A1');

writecell({'medium'},'Sorted.xlsx','Sheet','Track Disp','Range','F1');
writecell({'medium'},'Sorted.xlsx','Sheet','Mean Sp','Range','F1');
writecell({'medium'},'Sorted.xlsx','Sheet','Max Sp','Range','F1');
writecell({'medium'},'Sorted.xlsx','Sheet','Total Dist','Range','F1');
writecell({'medium'},'Sorted.xlsx','Sheet','Max Dist','Range','F1');
writecell({'medium'},'Sorted.xlsx','Sheet','Confin Ratio','Range','F1');
writecell({'medium'},'Sorted.xlsx','Sheet','Mean Straightline
Speed','Range','F1');
writecell({'medium'},'Sorted.xlsx','Sheet','Fwd Prog','Range','F1');
writecell({'medium'},'Sorted.xlsx','Sheet','Mean Y Rate','Range','F1');

writecell({'high'},'Sorted.xlsx','Sheet','Track Disp','Range','K1');
writecell({'high'},'Sorted.xlsx','Sheet','Mean Sp','Range','K1');
writecell({'high'},'Sorted.xlsx','Sheet','Max Sp','Range','K1');
writecell({'high'},'Sorted.xlsx','Sheet','Total Dist','Range','K1');
writecell({'high'},'Sorted.xlsx','Sheet','Max Dist','Range','K1');
writecell({'high'},'Sorted.xlsx','Sheet','Confin Ratio','Range','K1');
writecell({'high'},'Sorted.xlsx','Sheet','Mean Straightline
Speed','Range','K1');
writecell({'high'},'Sorted.xlsx','Sheet','Fwd Prog','Range','K1');
writecell({'high'},'Sorted.xlsx','Sheet','Mean Y Rate','Range','K1');

writecell({'all'},'Sorted.xlsx','Sheet','Track Disp','Range','P1');
writecell({'all'},'Sorted.xlsx','Sheet','Mean Sp','Range','P1');
writecell({'all'},'Sorted.xlsx','Sheet','Max Sp','Range','P1');
writecell({'all'},'Sorted.xlsx','Sheet','Total Dist','Range','P1');
writecell({'all'},'Sorted.xlsx','Sheet','Max Dist','Range','P1');
writecell({'all'},'Sorted.xlsx','Sheet','Confin Ratio','Range','P1');
writecell({'all'},'Sorted.xlsx','Sheet','Mean Straightline
Speed','Range','P1');
writecell({'all'},'Sorted.xlsx','Sheet','Fwd Prog','Range','P1');
writecell({'all'},'Sorted.xlsx','Sheet','Mean Y Rate','Range','P1');


% x = 1; %initialize x
% while x < 10
%
%     A = char(x+64);
%     B = char(x+65);
%     C = char(x+66);
%     loc1 = [A '2'];
%     loc2 = [B '2'];
%     loc3 = [C '2'];
%
%     writecell({'N spots'},'Sorted.xlsx','Sheet','Track
Disp','Range',loc1);
```

```
%     writecell({'N spots'},'Sorted.xlsx','Sheet','Mean Sp','Range',loc1);
%     writecell({'N spots'},'Sorted.xlsx','Sheet','Max Sp','Range',loc1);
%     writecell({'N spots'},'Sorted.xlsx','Sheet','Total
Dist','Range',loc1);
%     writecell({'N spots'},'Sorted.xlsx','Sheet','Max
Dist','Range',loc1);
%     writecell({'N spots'},'Sorted.xlsx','Sheet','Confin
Ratio','Range',loc1);
%     writecell({'N spots'},'Sorted.xlsx','Sheet','Mean Straightline
Speed','Range',loc1);
%     writecell({'N spots'},'Sorted.xlsx','Sheet','Fwd
Prog','Range',loc1);
%     writecell({'N spots'},'Sorted.xlsx','Sheet','Mean Y
Rate','Range',loc1);
%
%     writecell({'Track Disp'},'Sorted.xlsx','Sheet','Track
Disp','Range',loc2);
%     writecell({'Track Disp'},'Sorted.xlsx','Sheet','Mean
Sp','Range',loc2);
%     writecell({'Track Disp'},'Sorted.xlsx','Sheet','Max
Sp','Range',loc2);
%     writecell({'Track Disp'},'Sorted.xlsx','Sheet','Total
Dist','Range',loc2);
%     writecell({'Track Disp'},'Sorted.xlsx','Sheet','Max
Dist','Range',loc2);
%     writecell({'Track Disp'},'Sorted.xlsx','Sheet','Confin
Ratio','Range',loc2);
%     writecell({'Track Disp'},'Sorted.xlsx','Sheet','Mean Straightline
Speed','Range',loc2);
%     writecell({'Track Disp'},'Sorted.xlsx','Sheet','Fwd
Prog','Range',loc2);
%     writecell({'Track Disp'},'Sorted.xlsx','Sheet','Mean Y
Rate','Range',loc2);
%
%     writecell({'Data'},'Sorted.xlsx','Sheet','Mean Sp','Range',loc3);
%     writecell({'Data'},'Sorted.xlsx','Sheet','Max Sp','Range',loc3);
%     writecell({'Data'},'Sorted.xlsx','Sheet','Total Dist','Range',loc3);
%     writecell({'Data'},'Sorted.xlsx','Sheet','Max Dist','Range',loc3);
%     writecell({'Data'},'Sorted.xlsx','Sheet','Confin
Ratio','Range',loc3);
%     writecell({'Data'},'Sorted.xlsx','Sheet','Mean Straightline
Speed','Range',loc3);
%     writecell({'Data'},'Sorted.xlsx','Sheet','Fwd Prog','Range',loc3);
%     writecell({'Data'},'Sorted.xlsx','Sheet','Mean Y
Rate','Range',loc3);
%
%     x = x+4; %update x
%
% end

lowCA = {};
medCA = {};
highCA = {};
total = 0;
```

```
for x1 = 1:L1

    FN = allFiles(x1).name; %get the specific file name as a string
    PlateName = FN(1:end-5); %Get plate ID
    DataCA = {PlateName};
    plates = [plates PlateName];
    L2 = length(plates); %number of plates

    file = fullfile(projectdir,FN); %open specific file
    ca = readtable(file); %read the file and make it a table
    ca = table2cell(ca); %turn table into a CA with columns of data
    nSpots = ca(:,4);
    trackDisp = ca(:,13);
    meanSp = ca(:,17);
    maxSp = ca(:,18);
    totalDist = ca(:,23);
    maxDist = ca(:,24);
    confinRatio = ca(:,25);
    MSS = ca(:,26);
    fwdProg = ca(:,27);
    MYR = ca(:,28);
    allData = [nSpots trackDisp meanSp maxSp totalDist maxDist confinRatio
MSS fwdProg MYR];
    [L3,~] = size(nSpots); %number of datapoints for the plate

    for x2 = L3:-1:1

        if nSpots{x2} < 60
            allData(x2,:) = [];
        end

    end

    [L4,~] = size(allData);

    plateID = {};
    for x4 = 1:L4
        plateID = [plateID; PlateName];
    end

    allData = [allData plateID];
    trackDisp = allData(:,2);

    for x3 = 1:L4
        E = trackDisp{x3};
        row = allData(x3,:);
        if E < 10
            lowCA = [lowCA; row];
        elseif E > 10 & E < 20
            medCA = [medCA; row];
        else
            highCA = [highCA; row];
        end
```

```
    end

    [r1,~] = size(lowCA);
    lowPct = r1/L4*100;
    [r2,~] = size(medCA);
    medPct = r2/L4*100;
    [r3,~] = size(highCA);
    highPct = r3/L4*100;


    total = total+L4;

end

[r,~] = size(lowCA);
if r > 0
    lowTrackDisp = [lowCA(:,1:2) lowCA(:,11)];
    r = num2str(r);
    loc4 = ['B1:D' r];
    lowMeanSP = [lowCA(:,1:3) lowCA(:,11)];
    lowMedianSP = [lowCA(:,1:2) lowCA(:,4) lowCA(:,11)];
    lowTotDist = [lowCA(:,1:2) lowCA(:,5) lowCA(:,11)];
    lowMaxDist = [lowCA(:,1:2) lowCA(:,6) lowCA(:,11)];
    lowConfinRatio = [lowCA(:,1:2) lowCA(:,7) lowCA(:,11)];
    lowMSS = [lowCA(:,1:2) lowCA(:,8) lowCA(:,11)];
    lowfwdProg = [lowCA(:,1:2) lowCA(:,9) lowCA(:,11)];
    lowMYR = [lowCA(:,1:2) lowCA(:,10:11)];
    loc5 = ['B1:E' r];
else
    lowTrackDisp = {0 0};
    lowMeanSP = {0 0 0};
    lowMedianSP = {0 0 0 0};
    lowTotDist = {0 0 0 0};
    lowMaxDist = {0 0 0 0};
    lowConfinRatio = {0 0 0 0};
    lowMSS = {0 0 0 0};
    lowfwdProg = {0 0 0 0};
    lowMYR = {0 0 0 0};
    loc4 = ['B1:D1'];
    loc5 = ['B1:E1'];
end

    writecell(lowTrackDisp,'Sorted.xlsx','Sheet','Track
Disp','Range',loc4);
    writecell(lowMeanSP,'Sorted.xlsx','Sheet','Mean Sp','Range',loc5);
    writecell(lowMedianSP,'Sorted.xlsx','Sheet','Max Sp','Range',loc5);
    writecell(lowTotDist,'Sorted.xlsx','Sheet','Total Dist','Range',loc5);
    writecell(lowMaxDist,'Sorted.xlsx','Sheet','Max Dist','Range',loc5);
    writecell(lowConfinRatio,'Sorted.xlsx','Sheet','Confin
Ratio','Range',loc5);
    writecell(lowMSS,'Sorted.xlsx','Sheet','Mean Straightline
Speed','Range',loc5);
    writecell(lowfwdProg,'Sorted.xlsx','Sheet','Fwd Prog','Range',loc5);
```

```
    writecell(lowMYR,'Sorted.xlsx','Sheet','Mean Y Rate','Range',loc5);


% [r,~] = size(lowCA);
%     ROK1 = 0;
%     ROI2 = 0;
%     ROI3 = 0;
%     for x4 = 1:r
%         plateID = lowCA{x4,11};
%         plateID = str2num(plateID(end));
%         if plateID == 1
%             ROK1 = ROK1 + 1;
%         elseif plateID == 2
%             ROI2 = ROI2 + 1;
%         else
%             ROI3 = ROI3 + 1;
%         end
%     end
%     ROK1 = ROK1/total*100;
%     ROI2 = ROI2/total*100;
%     ROI3 = ROI3/total*100;
%     lowPct = {ROK1, ROI2, ROI3};
%     writecell(lowPct,'Sorted.xlsx','Sheet','Pcts','Range','A1:C1');

[r,~] = size(medCA);
if r > 0
    medTrackDisp = [medCA(:,1:2) medCA(:,11)];
    r = num2str(r+3);
    medMeanSP = [medCA(:,1:3) medCA(:,11)];
    medMedianSP = [medCA(:,1:2) medCA(:,4) medCA(:,11)];
    medTotDist = [medCA(:,1:2) medCA(:,5) medCA(:,11)];
    medMaxDist = [medCA(:,1:2) medCA(:,6) medCA(:,11)];
    medConfinRatio = [medCA(:,1:2) medCA(:,7) medCA(:,11)];
    medMSS = [medCA(:,1:2) medCA(:,8) medCA(:,11)];
    medfwdProg = [medCA(:,1:2) medCA(:,9) medCA(:,11)];
    medMYR = [medCA(:,1:2) medCA(:,10:11)];
    loc4 = ['G1:I' r];
    loc5 = ['G1:J' r];
else
    medTrackDisp = {0 0};
    medMeanSP = {0 0 0};
    medMedianSP = {0 0 0 0};
    medTotDist = {0 0 0 0};
    medMaxDist = {0 0 0 0};
    medConfinRatio = {0 0 0 0};
    medMSS = {0 0 0 0};
    medfwdProg = {0 0 0 0};
    medMYR = {0 0 0 0};
    loc4 = ['G1:I1'];
    loc5 = ['G1:J1'];
end
```

```
    writecell(medTrackDisp,'Sorted.xlsx','Sheet','Track
Disp','Range',loc4);
    writecell(medMeanSP,'Sorted.xlsx','Sheet','Mean Sp','Range',loc5);
    writecell(medMedianSP,'Sorted.xlsx','Sheet','Max Sp','Range',loc5);
    writecell(medTotDist,'Sorted.xlsx','Sheet','Total Dist','Range',loc5);
    writecell(medMaxDist,'Sorted.xlsx','Sheet','Max Dist','Range',loc5);
    writecell(medConfinRatio,'Sorted.xlsx','Sheet','Confin
Ratio','Range',loc5);
    writecell(medMSS,'Sorted.xlsx','Sheet','Mean Straightline
Speed','Range',loc5);
    writecell(medfwdProg,'Sorted.xlsx','Sheet','Fwd Prog','Range',loc5);
    writecell(medMYR,'Sorted.xlsx','Sheet','Mean Y Rate','Range',loc5);



%     [r,~] = size(medCA);
%     ROK1 = 0;
%     ROI2 = 0;
%     ROI3 = 0;
%     for x4 = 1:r
%         plateID = medCA{x4,11};
%         plateID = str2num(plateID(end));
%         if plateID == 1
%             ROK1 = ROK1 + 1;
%         elseif plateID == 2
%             ROI2 = ROI2 + 1;
%         else
%             ROI3 = ROI3 + 1;
%         end
%     end
%     ROK1 = ROK1/total*100;
%     ROI2 = ROI2/total*100;
%     ROI3 = ROI3/total*100;
%     medPct = {ROK1, ROI2, ROI3};
%     writecell(medPct,'Sorted.xlsx','Sheet','Pcts','Range','A2:C2');

[r,~] = size(highCA);
if r>0
    highTrackDisp = [highCA(:,1:2) highCA(:,11)];
    r = num2str(r+3);
    highMeanSP = [highCA(:,1:3) highCA(:,11)];
    highMedianSP = [highCA(:,1:2) highCA(:,4) highCA(:,11)];
    highTotDist = [highCA(:,1:2) highCA(:,5) highCA(:,11)];
    highMaxDist = [highCA(:,1:2) highCA(:,6) highCA(:,11)];
    highConfinRatio = [highCA(:,1:2) highCA(:,7) highCA(:,11)];
    highMSS = [highCA(:,1:2) highCA(:,8) highCA(:,11)];
    highfwdProg = [highCA(:,1:2) highCA(:,9) highCA(:,11)];
    highMYR = [highCA(:,1:2) highCA(:,10:11)];
    loc4 = ['L1:N' r] ;
    loc5 = ['L1:O' r];
else
    highTrackDisp = {0 0};
    highMeanSP = {0 0 0};
    highMedianSP = {0 0 0 0};
```

```
    highTotDist = {0 0 0 0};
    highMaxDist = {0 0 0 0};
    highConfinRatio = {0 0 0 0};
    highMSS = {0 0 0 0};
    highfwdProg = {0 0 0 0};
    highMYR = {0 0 0 0};
    loc4 = ['L1:N1'];
    loc5 = ['L1:O1'];
end

    writecell(highTrackDisp,'Sorted.xlsx','Sheet','Track
Disp','Range',loc4);
    writecell(highMeanSP,'Sorted.xlsx','Sheet','Mean Sp','Range',loc5);
    writecell(highMedianSP,'Sorted.xlsx','Sheet','Max Sp','Range',loc5);
    writecell(highTotDist,'Sorted.xlsx','Sheet','Total
Dist','Range',loc5);
    writecell(highMaxDist,'Sorted.xlsx','Sheet','Max Dist','Range',loc5);
    writecell(highConfinRatio,'Sorted.xlsx','Sheet','Confin
Ratio','Range',loc5);
    writecell(highMSS,'Sorted.xlsx','Sheet','Mean Straightline
Speed','Range',loc5);
    writecell(highfwdProg,'Sorted.xlsx','Sheet','Fwd Prog','Range',loc5);
    writecell(highMYR,'Sorted.xlsx','Sheet','Mean Y Rate','Range',loc5);


allTrackDisp = [lowTrackDisp(:,2); medTrackDisp(:,2); highTrackDisp(:,2)];
allMeanSP = [lowMeanSP(:,3); medMeanSP(:,3); highMeanSP(:,3)];
allMedianSP = [lowMedianSP(:,3); medMedianSP(:,3); highMedianSP(:,3)];
allTotDist = [lowTotDist(:,3); medTotDist(:,3); highTotDist(:,3)];
allMaxDist = [lowMaxDist(:,3); medMaxDist(:,3); highMaxDist(:,3)];
allConfinRatio = [lowConfinRatio(:,3); medConfinRatio(:,3);
highConfinRatio(:,3)];
allMSS = [lowMSS(:,3); medMSS(:,3); highMSS(:,3)];
allfwdProg = [lowfwdProg(:,3); medfwdProg(:,3); highfwdProg(:,3)];
allMYR = [lowMYR(:,3); medMYR(:,3); highMYR(:,3)];

[a,~] = size(allTrackDisp);
loc6 = ['Q1:Q' num2str(a)];
    writecell(allTrackDisp,'Sorted.xlsx','Sheet','Track
Disp','Range',loc6);

[a,~] = size(allMeanSP);
loc6 = ['Q1:Q' num2str(a)];
    writecell(allMeanSP,'Sorted.xlsx','Sheet','Mean Sp','Range',loc6);

[a,~] = size(allMedianSP);
loc6 = ['Q1:Q' num2str(a)];
    writecell(allMedianSP,'Sorted.xlsx','Sheet','Max Sp','Range',loc6);

[a,~] = size(allTotDist);
loc6 = ['Q1:Q' num2str(a)];
    writecell(allTotDist,'Sorted.xlsx','Sheet','Total Dist','Range',loc6);

[a,~] = size(allMaxDist);
```

```
loc6 = ['Q1:Q' num2str(a)];
    writecell(allMaxDist,'Sorted.xlsx','Sheet','Max Dist','Range',loc6);

[a,~] = size(allConfinRatio);
loc6 = ['Q1:Q' num2str(a)];
    writecell(allConfinRatio,'Sorted.xlsx','Sheet','Confin
Ratio','Range',loc6);

[a,~] = size(allMSS);
loc6 = ['Q1:Q' num2str(a)];
    writecell(allMSS,'Sorted.xlsx','Sheet','Mean Straightline
Speed','Range',loc6);

[a,~] = size(allfwdProg);
loc6 = ['Q1:Q' num2str(a)];
    writecell(allfwdProg,'Sorted.xlsx','Sheet','Fwd Prog','Range',loc6);

[a,~] = size(allMYR);
loc6 = ['Q1:Q' num2str(a)];
    writecell(allMYR,'Sorted.xlsx','Sheet','Mean Y Rate','Range',loc6);



%    [r,~] = size(highCA);
%     ROK1 = 0;
%     ROI2 = 0;
%     ROI3 = 0;
%     for x4 = 1:r
%         plateID = highCA{x4,11};
%         plateID = str2num(plateID(end));
%         if plateID == 1
%             ROK1 = ROK1 + 1;
%         elseif plateID == 2
%             ROI2 = ROI2 + 1;
%         else
%             ROI3 = ROI3 + 1;
%         end
%     end
%     ROK1 = ROK1/total*100;
%     ROI2 = ROI2/total*100;
%     ROI3 = ROI3/total*100;
%     highPct = {ROK1, ROI2, ROI3};
%     writecell(highPct,'Sorted.xlsx','Sheet','Pcts','Range','A3:C3');

disp('Done')

end
```

Percentages MATLAB Code

```
function [Data] = Percentages(projectdir)

allFiles = dir(projectdir); %ST with all the files
```

```
allFiles(1:2) = []; %removes the first 2 rows of the ST because they do
not contain data

L1 = length(allFiles); % # of devices in the condition

DataCA = {}; %create cell aray of data to write to the xlsx file
plates = {};
totalLengths = [0];
total = 0;
percentages = {'PlateID' 'total count' 'low count' 'mid count' 'high
count' 'Low%' 'Mid%' 'High%' '%Sum'};

for x1 = 1:L1

    FN = allFiles(x1).name; %get the specific file name as a string
    PlateName = FN(1:end-5); %Get plate ID
    DataCA = {PlateName};
    plates = [plates PlateName];
    L2 = length(plates); %number of plates

    file = fullfile(projectdir,FN); %open specific file
    ca = readtable(file); %read the file and make it a table
    ca = table2cell(ca); %turn table into a CA with columns of data
    nSpots = ca(:,4);
    trackDisp = ca(:,13);
    meanSp = ca(:,17);
    medianSp = ca(:,20);
    totalDist = ca(:,23);
    maxDist = ca(:,24);
    confinRatio = ca(:,25);
    MSS = ca(:,26);
    fwdProg = ca(:,27);
    MYR = ca(:,28);
    allData = [nSpots trackDisp meanSp medianSp totalDist maxDist
confinRatio MSS fwdProg MYR];
    [L3,~] = size(nSpots); %number of datapoints for the plate

    for x2 = L3:-1:1

        if nSpots{x2} < 21
            allData(x2,:) = [];
        end

    end

    [L4,~] = size(allData);

    plateID = {};
    for x4 = 1:L4
        plateID = [plateID; PlateName];
    end

    allData = [allData plateID];
    trackDisp = allData(:,2);
```

```
    lowCA = {};
    medCA = {};
    highCA = {};

    for x3 = 1:L4
        E = trackDisp{x3};
        row = allData(x3,:);
        if E < 10
            lowCA = [lowCA; row];
        elseif E > 10 & E < 20
            medCA = [medCA; row];
        else
            highCA = [highCA; row];
        end

    end

    [r1,~] = size(lowCA);
    lowPct = r1/L4*100;
    [r2,~] = size(medCA);
    medPct = r2/L4*100;
    [r3,~] = size(highCA);
    highPct = r3/L4*100;
    sum = lowPct + medPct + highPct;

    row = {PlateName L4 r1 r2 r3 lowPct medPct highPct sum};
    percentages = [percentages; row];

    total = total+L4;

end

Sheetname = projectdir;
writecell(percentages,'Percentages.xlsx','Sheet',Sheetname)

disp('Done')

end
```
